# Crystallization-Induced Coordination Diversity of Cu(I)-Pyridine Halide Complexes Resulting in Optical Tunability

**DOI:** 10.1021/acs.inorgchem.5c05554

**Published:** 2026-03-03

**Authors:** Mariia Beliaeva, Ondřej Mrózek, Igor O. Koshevoy, Andreas Steffen, Andrey Belyaev

**Affiliations:** † Department of Chemistry and Sustainable Technology, 163043 University of Eastern Finland, Joensuu 80101, Finland; ‡ Department of Chemistry and Chemical Biology, 14311 TU Dortmund University, Dortmund 44227, Germany; § Institute of Inorganic Chemistry of the Czech Academy of Sciences, Husinec-Řež 250 68, Czech Republic

## Abstract

Copper­(I) derivatives have emerged as a versatile class of luminescent and photoactive materials, combining earth abundance, structural adaptability, and rich excited-state dynamics that enable their application in luminescent devices, photocatalysis, and sensing technologies. Herein, we report a family of copper­(I) pyridine halide complexes supported by a 4-(*N,N*-dimethylamino)­pyridine (DMAP) ligand, featuring crystallization-induced diversity of coordination motifs. The variation of halides and stoichiometry of [Cu­(NCMe)_4_]­BF_4_/CuX (X = Cl, Br, I) precursors enabled the selective isolation of a series of cationic/neutral mono- and multinuclear hybrid species, namely, [(DMAP)_2_Cu]­BF_4_, [(DMAP)­CuCl], [(DMAP)_4_Cu_2_(μ_2_–X)]­BF_4_ (X = Cl, Br), [(DMAP)_4_Cu_4_(μ_2_-Br)_2_(μ_3_-Br)_2_]­[(DMAP)_2_Cu]_2_(BF_4_)_2_, and [(DMAP)_2_Cu_2_(μ_2_–I)_2_]_2_[(DMAP)_2_Cu]_3_(BF_4_)_3_. Single-crystal X-ray diffraction revealed that the bridging mode of halides is decisive in governing packing topology, nuclearity, and structural arrangement of metal/cluster centers. Photophysical studies demonstrated tunable solid-state phosphorescence spanning from sky blue (478 nm) to deep red (640 nm), with quantum yields and radiative rates reaching 0.41 and 7.3 × 10^4^ s^–1^, respectively. Advanced photophysical studies combined with DFT/TD-DFT calculations facilitated the untangling of structural characteristics responsible for control over photophysical properties such as triplet formation and its (non)­radiative decay or the nature of luminescent excited states, defining multiple structure–property relationships. These results establish an effective strategy to unlock new coordination motifs in copper­(I) halide chemistry and to achieve broadband optical tunability in earth-abundant photoactive materials by means of crystallization tools.

## Graphical abstract

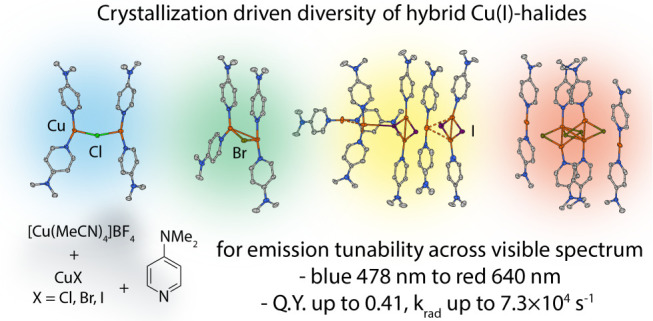

## Introduction

Over the past few decades, the development of photofunctional materials for the fields of high-end optical technologies has been of primary importance. Their utilization comprises the construction of efficient daily life electronics,^1,2^ memory devices and optical switches,^3^ molecular bioprobes for targeted monitoring^4–6^ and a variety of responsive sensing systems.^7–11^ Such a strong boost of research in the above-mentioned areas has been driven to a noticeable extent by attractive photophysical features of the luminescent coordination complexes. For instance, heavy metal ions in organometallic and metal–organic emissive entities are known to promote efficient spin–orbit coupling (SOC), which facilitates spin-forbidden singlet-to-triplet (S_1_-T_n_) intersystem crossing, and consequently permits fast radiative relaxation of the triplet state.^12,13^ In electroluminescent devices, this beneficial feature is manifested by efficient harvesting of up to 100% of excitons after charge recombination, which allows for reaching internal quantum efficiencies close to unity.^14–17^

Among photoemissive transition metal complexes, copper­(I) derivatives with d^10^ electronic configuration are found to be the most prospective replacement for traditionally used phosphorescent species containing costly noble metals, e.g., iridium­(III), platinum­(II) gold­(I/III) or ruthenium­(II), owing to the low cost, earth abundance, and comparatively lower toxicity of copper.^18–20^ Despite its relatively small SOC constant in comparison to 5d elements, which prohibits equally efficient phosphorescence, fast radiative decay involving triplet excited states can be realized indirectly by thermally activated delayed fluorescence (TADF). As a prerequisite, the ligand sphere around the copper­(I) center requires sophisticated design, aiming at a small energy gap ΔE­(S_1_-T_1_) between the lowest singlet (S_1_) and triplet (T_1_) excited states to allow for Boltzmann equilibrium between them at room temperature.^21,22^ Such a strategy has delivered a selection of copper­(I)-based materials demonstrating TADF, which is a valuable property to improve the efficiency of solid-state-lighting devices.^19,21,23–25^

The diversity of known luminescent copper­(I) complexes as well as routes to their tunability rapidly expands day to day. A rational selection of stabilizing ligands allows adjusting the energies of the lowest unoccupied and highest occupied molecular orbitals (LUMO and HOMO) and tuning the optical band gap. For instance, families of emitters constructed of *N*-heterocyclic carbenes and cyclic alkyl/aryl-amino carbenes provide several benefits including straightforward synthesis, fast relaxation mechanisms, and wide tunability of the emission, which covers the entire visible range extending to the near IR region.^26–29^ Stabilizing ligands such as halides and pseudohalides (Cl, Br, I, CN, SCN) can further increase SOC via the heavy atom effect in addition to the copper atom. Such entities have proven to be of general interest for (photo)­catalysis and photonic applications. In turn, copper halides in combination with *N*-heteroaromatic^30–33^ or soft base ligands (i.e., phosphines, sulfides or their mixed bridged phosphine-sulfides),^34–36^ offer rich structural and photophysical diversity. Among these structures, the most common species are (i) CuX­(L)_n_ (L = organic ligand) monometallic compounds; (ii) Cu_2_X_2_L_n_ butterfly shaped rhomboid dimers; (iii) Cu_4_(μ_3_-X)_4_L_4_ cubane tetramers; and (iv) (CuXL)_∞_ staircase polymeric structures.^37–42^ Araki et al. demonstrated that wide changes in the emission color from deep blue to red (450–707 nm) can be achieved for isostructural binuclear copper­(I) halide complexes [CuX­(PPh_3_)­L]_2_ by rational selection of terminal *N*-heteroaromatic ligands (L = pyrazine, pyrimidine, piperazine, etc.).^43^ In another example, simple alteration of the linking group from phenyl to pyridine or pyrazine in the diphosphine moiety shifts the wavelength maximum from blue to green and ultimately to orange-red, assigned to significant stabilization of the LUMO participating in the charge-transfer (CT) excited state.^44^ Structural diversity was exemplified in the seminal work of S. Wang et al.^39^ Their approach highlighted (iso)­quinoline/piperidine copper­(I) iodides, including 1D chains, rhomboid dimers, cubane, and octahedral tetramers, exhibiting emissions attributed to a combination of halide/metal-to-ligand charge transfer (X/MLCT) and cluster-centered (CC) transitions. However, structural variability not only alters the luminescence wavelength, but also induces interesting photophysical phenomena, including nonlinear optical properties,^45,46^ white light emission,^41,47^ vapo-,^48–50^ thermo- and mechanochromism.^51–55^ For instance, Thompson and co-workers reported dual emission of octahedrally shaped copper iodide clusters Cu_4_I_4_(R_2_PCH_2_py)_2_, where bulky substituents (R = phenyl, cyclohexyl) control the population and radiative decay of a second excited state of CC origin.^38^ B. Huitorel et al. in 2017 investigated a rare example of mechanically induced solid-state isomerization of Cu_4_I_4_(PPh_3_)_4_ cluster from the staircase to cubane-type motif.^56^ The observed structural transition causes a highly contrasting 100 nm bathochromic shift and an increase of quantum efficiency from a nearly nonemissive state with Φ ≤ 0.01 to an appreciable intensity of Φ = 0.13.

Taking into account the attractiveness of cuprous emitters with a general formula Cu_*x*_X_*y*_L_n_,^19^ herein we attempted to study the coordination behavior and structure–property relationships of the complexes obtained from mixed [Cu­(NCMe)_4_]­BF_4_/CuX salts (X = Cl, Br, I) supported by a monodentate 4-(*N*,*N*-dimethylamino)­pyridine ligand (DMAP). Via halide variation, we demonstrate that the nature of the bridging halides plays a decisive role in the formation of molecular motifs and packing. This strategy delivered the yet unknown family of mononuclear [(DMAP)_2_Cu]­(BF_4_) and [(DMAP)­CuCl], and multinuclear [(DMAP)_4_Cu_2_(μ_2_-X)]­(BF_4_) (X = Cl, Br), [(DMAP)_4_Cu_4_(μ_2_-Br)_2_(μ_3_-Br)_2_]­[(DMAP)_2_Cu]_2_(BF_4_)_2_ and [(DMAP)_2_Cu_2_(μ_2_–I)_2_]_2_[(DMAP)_2_Cu]_3_(BF_4_)_3_ copper­(I) halide derivatives with tunable phosphorescence spanning from sky-blue (475 nm) to red (640 nm) and Φ reaching 0.41 at room temperature.

## Experimental Section

### General Comments

All reactions and manipulations were performed under a nitrogen atmosphere in a Glovebox Systemtechnik MEGA E-Line and standard Schlenk techniques.^57^ All glassware was heatgun- or oven-dried overnight at 120 °C prior to use. Anhydrous solvents (tetrahydrofuran (THF), diethyl ether (Et_2_O), acetonitrile, pentane, dichloromethane (DCM) and cyclohexane (CHX)) were obtained from solvent purification system PureSolv MD 7 and further degassed by freeze–pump–thaw cycle technique. Deuterated DCM was distilled over CaH_2_, followed by vacuum-transfer and stored under vacuum-high-temperature-activated 4 Å molecular sieves. DMAP, and copper­(I) iodide (CuI) were purchased from commercial suppliers and used without additional purification. Copper­(I) chloride (CuCl), copper­(I) bromide (CuBr) and tetrakis­(acetonitrile)­copper­(I) tetrafluoroborate ([Cu­(MeCN)_4_]­BF_4_) were prepared by the published methods.^58,59^ The solution 1D ^1^H, ^11^B, ^13^C, ^19^F and 2D ^1^H–^15^N HMBC NMR spectra were recorded on Bruker 400 Avance, Bruker Avance III HD NanoBay, Bruker Avance NEO and Agilent DD2 spectrometers. Microanalyses were carried out in the analytical laboratories of the University of Eastern Finland and TU Dortmund University. IR spectra of the crystalline material were collected with a PerkinElmer FT/IR spectrometer Spectrum 3 equipped with an inert mantle compartment. After obtaining crystalline material, all samples were subjected to high-vacuum drying (10^–2^–10^–3^ mbar) for 12 h at room temperature.

### Synthesis

#### [(DMAP)_2_Cu]­(BF_4_) (**1**)

To a solution of DMAP (244 mg, 2.0 mmol, 2 equiv) in THF (20 mL), [Cu­(MeCN)_4_]­BF_4_ (315 mg, 1.0 mmol, 1 equiv) was added in one portion under stirring at room temperature. A white precipitate formed within several minutes and the mixture was stirred for an additional 4 h. The resulting suspension was cooled to −20 °C and the product was collected by filtration. The obtained solid was washed with cold THF (3 × 5 mL), Et_2_O (3 × 15 mL) and dried under vacuum. Recrystallization by layering of a DCM solution (10 mL) with CHX (15 mL) at room temperature gave white crystalline material within 2 days. Yield: 250 mg (66%). ^1^H NMR (400 MHz, CD_2_Cl_2_, 298 K, δ/ppm): 8.06 (br s, 2H, H_1,4_-DMAP), 6.57 (br s, 2H, H_2,3_-DMAP), 3.05 (s, 6H, -NMe_2_). ^1^H NMR (400 MHz, CD_2_Cl_2_, 193 K, δ/ppm): 8.08 (br s, 2H, H_1,4_-DMAP), 6.57 (br s, 2H, H_2,3_-DMAP), 3.05 (s, 6H, -NMe_2_). ^13^C­{^1^H} NMR (150 MHz, CD_2_Cl_2_, 298 K, δ/ppm): 154.7 (s, C_3_), 149.5 (s, C_1,5_), 106.9 (s, C_2,4_), 39.0 (s, CH_3_). ^11^B­{^1^H} NMR (128 MHz, CD_2_Cl_2_, 298 K, δ/ppm): −1.1 (BF_4_). ^19^F NMR (376 MHz, CD_2_Cl_2_, 298 K, δ/ppm): −153.1 (BF_4_). ^15^N NMR (60 MHz, CD_2_Cl_2_, 298 K, δ/ppm): 65.8 (s, -NMe_2_). ESI^+^-MS (*m*/*z*): [M]^+^ 307.10 (calcd 307.09), [M+(DMAP)_2_]^+^ 551.27 (calcd 551.27). FTIR (neat powder, 298 K, ν/cm^–1^): 2911 (w), 1616 (s), 1535 (s), 1444 (m), 1392 (s), 1354 (m), 1283 (w), 1233 (s), 1047 (s), 1017 (s), 947 (s), 835 (s), 810 (s), 529 (s). Anal. Calc. for C_14_H_20_N_4_CuBF_4_ (%): C 42.60; H 5.11; N 14.20. Found: C 42.32; H 4.84; N 14.47.

#### [(DMAP)­CuCl] (**2**)

A suspension of CuCl (227 mg, 2.3 mmol, 1 equiv) and DMAP (280 mg, 2.3 mmol, 1 equiv) in THF (10 mL) was stirred for 4 h at room temperature. A white precipitate was collected, washed with THF (3 × 5 mL), Et_2_O (3 × 10 mL), pentane (3 × 10 mL), and dried under vacuum. Recrystallization by layering of a DCM solution of **2** with CHX at room temperature gave colorless needles within 3 days. Crystals were filtered off, washed with Et_2_O (3 × 10 mL) and dried under vacuum. Yield 455 mg (95%). ^1^H NMR (600 MHz, CD_2_Cl_2_, 298 K, δ/ppm): 8.18 (br s, 2H, H_1,4_-DMAP), 6.55 (br d, *J*_HH_ 5 Hz 2H, H_2,3_-DMAP), 3.05 (s, 6H, -NMe_2_). ^13^C­{^1^H} NMR (150 MHz, CD_2_Cl_2_, 298 K, δ/ppm): 154.7 (s, C_3_), 149.5 (s, C_1,5_), 106.9 (s, C_2,4_), 39.0 (s, CH_3_). ^11^B­{^1^H} NMR (128 MHz, CD_2_Cl_2_, 298 K, δ/ppm): −1.1 (BF_4_). ^19^F NMR (376 MHz, CD_2_Cl_2_, 298 K, δ/ppm): −153.2 (BF_4_) ^15^N NMR (60 MHz, CD_2_Cl_2_, 298 K, δ/ppm): 65.5 (s, -NMe_2_). FTIR (neat powder, 298 K, ν/cm^–1^): 3105 (w), 3066 (w), 2573 (w), 1610 (s), 1540 (s), 1529 (s), 1458 (s), 1438 (s), 1392 (s), 1343 (s), 1286 (s), 1227 (s), 1189 (m), 1114 (m), 1070 (s), 1020 (s), 949 (s), 840 (w), 818 (s), 800 (s), 764 (w), 661 (m), 532 (s), 487 (s). Anal. Calc. for C_7_H_10_N_2_CuCl (%): C 38.02; H 4.56; N 12.67. Found: C 38.21; H 4.44; N 12.70.

#### [(DMAP)_4_Cu_2_(μ_2_-Cl)]­(BF_4_) (**3**)

*Route a*. A suspension of CuCl (50 mg, 0.5 mmol, 1 equiv), DMAP (244 mg, 2.0 mmol, 4 equiv) and [Cu­(MeCN)_4_]­BF_4_ (158 mg, 0.5 mmol, 1 equiv) in THF (20 mL) was stirred for 24 h. A white precipitate was collected, washed with THF (3 × 5 mL), Et_2_O (3 × 10 mL), pentane (3 × 10 mL), and dried under vacuum. Recrystallization by a gas-phase diffusion of Et_2_O (20 mL) into an acetonitrile solution (10 mL) of **3** at room temperature gave transparent block crystals within 4 days. Crystals were separated from the mother liquor, washed with Et_2_O (3 × 10 mL), and dried under vacuum. Yield: 307 mg (83%).

*Route b*. Alternatively, **1** (197 mg, 0.5 mmol, 1 equiv) and DMAP (122 mg, 1.0 mmol, 2 equiv) were added in one portion to a suspension of CuCl (50 mg, 0.5 mmol, 1 equiv) in DCM (10 mL). The reaction mixture was stirred for 2 h resulting in a clear solution, which was filtered through a glass fiber filter and evaporated to dryness under vacuum. White powder was washed with Et_2_O (3 × 15 mL). Recrystallization by a gas diffusion of Et_2_O into an acetonitrile (10 mL) solution of **3** at room temperature gave transparent block crystals within 4 days. Crystals were separated from the mother liquor, washed with Et_2_O (3 × 10 mL), and dried under vacuum. Yield 260 mg (71%). ^1^H NMR (600 MHz, CD_2_Cl_2_, 298 K, δ/ppm): 8.05 (br s, 2H, H_1,4_-DMAP), 6.47 (br d, *J*_HH_ 5 Hz 2H, H_2,3_-DMAP), 3.01 (s, 6H, -NMe_2_). ^1^H NMR (600 MHz, CD_2_Cl_2_, 193 K, δ/ppm): 8.05 (d, *J*_HH_ 6.7 Hz, 2H, H_1,4_-DMAP), 6.38 (d, *J*_HH_ 6.7 Hz, 2H, H_2,3_-DMAP), 2.97 (s, 6H, -NMe_2_). ^13^C­{^1^H} NMR (150 MHz, CD_2_Cl_2_, 298 K, δ/ppm): 155.0 (s, C_3_), 149.5 (s, C_1,5_), 107.0 (s, C_2,4_), 39.1 (s, CH_3_). ^11^B NMR (160 MHz, CD_2_Cl_2_, 298 K, δ/ppm): −1.1 (BF_4_). ^19^F NMR (471 MHz, CD_2_Cl_2_, 298 K, δ/ppm): −153.3 (BF_4_). ^15^N NMR (60 MHz, CD_2_Cl_2_, 298 K, δ/ppm): 69.0 (s, -NMe_2_). FTIR (neat powder, 298 K, ν/cm^–1^): 2918 (w), 1617 (s), 1536 (s), 1448 (m), 1388 (s), 1282 (w), 1231 (s), 1183 (w), 1096 (m), 1050 (s), 1019 (s), 946 (m), 801 (s), 523 (s), 483 (m). Anal. Calc. for C_28_H_40_N_8_Cu_2_ClBF_4_ (%): C 45.57; H 5.46; N 15.18. Found: C 45.81; H 5.34; N 15.03.

#### [(DMAP)_4_Cu_2_(μ_2_-Br)]­(BF_4_) (**4**)

Synthesized analogously to **3** from DMAP (244 mg, 2.0 mmol, 4 equiv), CuBr (72 mg, 0.5 mmol, 1 equiv) and [Cu­(MeCN)_4_]­BF_4_ (158 mg, 0.5 mmol, 1 equiv) in THF (20 mL) to give white crystalline material. Recrystallization by layering of a DCM solution (30 mL) of 4 with CHX (50 mL) at room temperature gave colorless transparent blocks within 3 days. Crystals were separated from the mother liquor, washed with Et_2_O (3 × 10 mL), and dried in a vacuum. Yield: 202 mg (52%). ^1^H NMR (600 MHz, CD_2_Cl_2_, 298 K, δ/ppm): 8.12 (br s, 2H, H_1,4_-DMAP), 6.55 (br s, 2H, H_2,3_-DMAP), 3.04 (s, 6H, -NMe_2_). ^1^H NMR (600 MHz, CD_2_Cl_2_, 193 K, δ/ppm): 8.08 (d, *J*_HH_ 6.5 Hz, 2H, H_1,4_-DMAP), 6.32 (d, *J*_HH_ 6.5 Hz, 2H, H_2,3_-DMAP), 2.95 (s, 6H, -NMe_2_). ^13^C­{^1^H} NMR (150 MHz, CD_2_Cl_2_, 298 K, δ/ppm): 155.0 (s, C_3_), 149.5 (s, C_1,5_), 107.0 (s, C_2,4_), 39.1 (s, CH_3_). ^11^B NMR (160 MHz, CD_2_Cl_2_, 298 K, δ/ppm): −1.1 (BF_4_). ^19^F NMR (471 MHz, CD_2_Cl_2_, 298 K, δ/ppm): −153.3 (BF_4_). ^15^N NMR (60 MHz, CD_2_Cl_2_, 298 K, δ/ppm): 66.0 (s, -NMe_2_). FTIR (neat powder, 298 K, ν/cm^–1^): 2914 (w), 2829 (w), 1612 (s), 1536 (s), 1449 (s), 1391 (s), 1344 (m), 1284 (w), 1227 (s), 1092 (s), 1051 (s), 1012 (s), 946 (s), 830 (s), 806 (s), 522 (s). Anal. Calc. for C_28_H_40_N_8_Cu_2_BrBF_4_ (%): C 42.98; H 5.15; N 14.32. Found: C 43.22; H 5.19; N 14.08.

#### [(DMAP)_4_Cu_4_(μ_2_-Br)_2_(μ_3_-Br)_2_]­[(DMAP)_2_Cu]_2_(BF_4_)_2_ (**5**)

Synthesized analogously to **3** (*Route a*) from DMAP (244 mg, 2.0 mmol, 4 equiv), CuBr (143 mg, 1.0 mmol, 2 equiv) and [Cu­(MeCN)_4_]­BF_4_ (158 mg, 0.5 mmol, 1 equiv) in THF (20 mL). Recrystallization by a gas-phase diffusion of Et_2_O (20 mL) into a DCM solution (10 mL) of **5** at room temperature gave transparent plate-type crystals within 2 days. Crystals were separated from the mother liquor, washed with Et_2_O (3 × 10 mL), and dried under vacuum. Yield: 410 mg (89%). *Route b*. Alternatively, a suspension of CuBr (14 mg, 0.1 mmol, 1 equiv) and 4 (80 mg, 0.1 mmol, 1 equiv) in DCM (5 mL) was stirred for 4 h resulting in a clear solution, which was filtered through a glass fiber filter and evaporated to dryness under vacuum. Recrystallization by a gas-phase diffusion of Et_2_O (10 mL) into a DCM solution (2 mL) of **5** at room temperature gave transparent plate-type crystals within 2 days. Crystals were separated from mother liquor, washed with Et_2_O (3 × 10 mL) and dried under vacuum. Yield: 87 mg (93%). ^1^H NMR (600 MHz, CD_2_Cl_2_, 298 K, δ/ppm): 8.09 (br d, *J*_HH_ 6.3 Hz, 2H, H_1,4_-DMAP), 6.59 (d, *J*_HH_ 6.3 Hz, 2H, H_2,3_-DMAP), 3.08 (s, 6H, -NMe_2_). ^1^H NMR (600 MHz, CD_2_Cl_2_, 193 K, δ/ppm): 8.04 (d, *J*_HH_ 6.9 Hz, 2H, H_1,4_-DMAP), 6.45 (d, *J*_HH_ 6.9 Hz, 2H, H_2,3_-DMAP), 3.00 (s, 6H, -NMe_2_). ^13^C­{^1^H} NMR (150 MHz, CD_2_Cl_2_, 298 K, δ/ppm): 155.0 (s, C_3_), 149.5 (s, C_1,5_), 106.9 (s, C_2,4_), 39.1 (s, CH_3_). ^11^B­{^1^H} NMR (128 MHz, CD_2_Cl_2_, 298 K, δ/ppm): −1.1 (BF_4_). ^19^F NMR (564 MHz, CD_2_Cl_2_, 298 K, δ/ppm): −153.1 (BF_4_). ^15^N NMR (60 MHz, CD_2_Cl_2_, 298 K, δ/ppm): 65.5 (s, -NMe_2_). FTIR (neat powder, 298 K, ν/cm^–1^): 2925 (s), 1611 (s), 1536 (s), 1439 (s), 1380 (s), 1351 (s), 1291 (m), 1225 (s), 1048 (s), 938 (s), 812 (s), 518 (s). Anal. Calc. for C_56_H_80_N_16_Cu_6_Br_4_B_2_F_8_ (%): C 36.32; H 4.35; N 12.10. Found: C 36.13; H 4.22; N 11.87.

#### [(DMAP)_2_Cu_2_I_2_]_2_[(DMAP)_2_Cu]_3_(BF_4_)_3_ (**6**)

Synthesized analogously to **3** from DMAP (255 mg, 2.1 mmol, 10 equiv), CuI (160 mg, 0.8 mmol, 4 equiv) and [Cu­(MeCN)_4_]­BF_4_ (198 mg, 0.6 mmol, 3 equiv) in THF (20 mL). Recrystallization by layering of a DCM/toluene (2/1, v/v) solution of **6** with pentane at room temperature gave a white crystalline material within 2 days. Crystals were separated from the mother liquor, washed with Et_2_O (3 × 10 mL), and dried under vacuum. Yield: 340 mg (67%). ^1^H NMR (600 MHz, CD_2_Cl_2_, 298 K, δ/ppm): 8.20 (br s, 2H, H_1,4_-DMAP), 6.56 (br s, *J*_HH_ 6.1 Hz, 2H, H_2,3_-DMAP), 3.06 (s, 6H, -NMe_2_). ^1^H NMR (600 MHz, CD_2_Cl_2_, 193 K, δ/ppm): 8.13 (br s, 2H, H_1,4_-DMAP), 6.48 (br d, *J*_HH_ 5.8 Hz, 2H, H_2,3_-DMAP), 3.01 (s, 6H, -NMe_2_). ^13^C­{^1^H} NMR (150 MHz, CD_2_Cl_2_, 298 K, δ/ppm): 155.0 (s, C_3_), 149.7 (s, C_1,5_), 107.0 (s, C_2,4_), 39.1 (s, CH_3_). ^11^B­{^1^H} NMR (128 MHz, CD_2_Cl_2_, 298 K, δ/ppm): −1.1 (BF_4_). ^19^F NMR (564 MHz, CD_2_Cl_2_, 298 K, δ/ppm): −153.1 (BF_4_). ^15^N NMR (60 MHz, CD_2_Cl_2_, 298 K, δ/ppm): 67.6 (s, -NMe_2_). FTIR (neat powder, 298 K, ν/cm^–1^): 2928 (w), 2819 (w), 1624 (s), 1544 (s), 1443 (m), 1397 (s), 1343 (m), 1287 (w), 1229 (s), 1094 (s), 1052 (s), 1022 (s), 946 (s), 845 (s), 800 (s), 740 (w), 522 (s), 484 (w). Anal. Calc. for C_70_H_100_N_20_Cu_7_I_4_B_3_F_12_ (%): C 35.53; H 4.14; N 11.51. Found: C 35.66; H 4.04; N 11.23.

### Single-Crystal X-ray Diffraction (scXRD) Analysis

The crystals of **1**–**6** were immersed in a film of NVH CODE 658 extra high viscosity oil (21000 centistokes at 23 °C, Cargille Laboratories, Inc.), mounted on a polyimide microloop (MicroMounts of MiTeGen), transferred to a stream of cold nitrogen (Bruker Kryoflex2), and measured at a temperature of 100–120 K. The X-ray diffraction data were collected on a Bruker D8 Venture diffractometer with a CMOS Photon 100 and multilayer optics monochromated MoKα (0.71073 Å) radiation (INCOATEC microfocus sealed tube). The frames were integrated with the Bruker SAINT software package using a narrow-frame algorithm. A semiempirical absorption correction (SADABS) was applied to all data. The APEX3 v2018.7–0 program package was used for cell refinements and data reductions. The structure was solved using the intrinsic phasing method,^60,61^ refined and visualized with the OLEX2–1.3 ^62^ and Diamond-4.6.4 programs.

All non-hydrogen atoms were refined anisotropically. Hydrogen atoms were included in structure factors calculations. All hydrogen atoms were assigned to idealized geometric positions riding on their parent atoms. The BF_4_^–^ counterions in **1** were disordered over two positions and were refined with occupancies of 0.56/0.44 and 0.53/0.47, respectively. The displacement parameters of the fluorine atoms in both components were constrained to be equal and were restrained so that their U_ij_ components approximate isotropic behavior. The unit cell of the **6** contains disordered solvent molecules of dichloromethane, which have been treated as a diffuse contribution to the overall scattering without specific atom positions by SQUEEZE/PLATON.^63^ Both single crystals of **4** and **6** were twinned. Thus, twin components **4** (0.85/0.15) and **6** (0.91/0.09) were resolved using the TWIN/BASF merohedral/pseudomerohedral twinning utility implemented in OLEX2. The crystallographic details are summarized in Table S1. Deposition numbers CCDC 2416137–2416142 contain the supplementary crystallographic data for this paper.

### Photophysical Studies

Both excitation and emission spectra of the solid samples were recorded on an Edinburgh Instruments FLS1000 spectrometer, equipped with a 450 W ozone-free Xenon arc lamp, double monochromators for the excitation and emission pathways, and a red-sensitive photomultiplier (PMT-980, 200–980 nm) as detector. The excitation and emission spectra were corrected using the standard corrections supplied by the manufacturer for the spectral power of the excitation source and the sensitivity of the detector. The quantum yields at 297 and 77 K were measured with an integrating cryosphere coupled with the FLS1000 spectrometer. The luminescence lifetimes were measured using a pulsed 60 W xenon microsecond flashlamp, with a repetition rate of 100 Hz, or LED pulsed laser diode (320 nm) with a multichannel scaling module (MCS). The emission was collected at right angles to the excitation source with the emission wavelength selected using a double-grated monochromator and detected by the respective PMT. Steady-state low-temperature measurements were performed utilizing an Oxford Optistat DN cryostat. Fluoracle and FAST spectrometer operating software and Origin Pro 2019 9.6.0 were used for data analysis and processing.

### Computational Details

DFT calculations were performed with the ORCA 6.1.0 software package.^64^ Geometry optimizations with tight convergence criteria were carried out using PBE0 functional^65,66^ with def2-TZVP basis set^67^ and Grimme-D3BJ empirical dispersion correction.^68,69^ To accelerate calculations, the SARC/J^70^ auxiliary basis set was used together with RI approximation. Solvents effects (THF, ε = 7.25) were accounted for by the implicit solvent model CPCM.^71^ Relativistic effects were accounted for by employing the ZORA method.^72^ The same level of theory was used for TD-DFT calculation of the first 20 singlet and triplet excited states. In the case of **3** and **4**, counteranions were omitted, while for **5** and **6**, only neutral complex clusters were modeled since linear compound **1**, found cocrystallized in structures of **5** and **6**, is nonemissive. The dimer-like species **2** ^**d**^ was optimized with constrained Cu and Cl atoms to reflect the structure found by single-crystal Xray diffraction analysis. Likewise, Cl–Cu–Cl angle and Cl–Cl distance for **3** were kept fixed within the optimization process. Electronic density differences at isovalues of 0.003 were prepared using the orca_plot module as implemented in ORCA 6.1.0. software and ChimeraX graphical software.^73^ The values of SOCMÊTOTAL listed in Table S16 are root-mean-square values of SOC matrix elements obtained by TD-DFT calculation with ORCA 6.1.0 using the “DOSOC true” keyword in the %TDDFT block.

## Results and Discussion

### Synthesis

Monometallic copper­(I) complexes **1** (66%) and 2 (95%) have been prepared by mixing DMAP with 0.5 and 1 equiv of the corresponding copper­(I) salt in THF, respectively, followed by subsequent recrystallization of the crude precipitates from DCM/CHX solvent combinations (Scheme 1). The addition of one equivalent of DMAP to an equimolar mixture of **1** and **2** resulted in a white powder of **3** in 83% yield. Alternatively, dinuclear species **3** can also be obtained by simple mixing of DMAP, [Cu­(NCMe)_4_]­BF_4_, and CuCl in THF. However, this route results in a slightly lower yield of 71%. In a similar fashion, utilizing CuBr as a starting precursor produces complex **4** only in 52% yield. Variation of the DMAP/[Cu­(NCMe)_4_]­BF_4_/CuCl ratio did not change the product composition. This is in contrast to the behavior of heavier halides CuX (X = Br, I), for which manipulating the stoichiometry of the reagents led to significant coordination diversity. Thus, it is important to notice that only recrystallization by a layering of a diluted DCM solution of **4** with an excess of CHX resulted in a selective formation of phase-pure compound depicted in Scheme 1, whereas other crystallization conditions gave mixtures of products. Changing the stoichiometry of DMAP/[Cu­(NCMe)_4_]­BF_4_/CuBr reagents to a 4:1:2 and a gas-phase diffusion of Et_2_O into a DCM solution as crystallization method selectively afforded **5** in high yield (93%), which consists of the staircase-like cluster [(DMAP)_4_Cu_4_(μ_2_-Br)_2_(μ_3_-Br)_2_ surrounded by two [(DMAP)_2_Cu]^+^ fragments and two BF_4_^–^. Finally, hybrid compound **6** (67%) of general composition [(DMAP)_2_Cu_2_(μ_2_-I_2_)]_2_[(DMAP)_2_Cu]_3_(BF_4_)_3_ was obtained starting from 4 equiv. CuI, 10 equiv of DMAP and 3 equiv of Cu­(NCMe)_4_BF_4_ salt. Layering of a DCM/toluene solution with pentane led to the precipitation of phase-pure material. All attempts to replicate iodide structural analogues of molecules **3**–**5** have failed and ended up with mixtures of the staircase hexamer (DMAP)_6_Cu_6_(μ_2_-I)_2_(μ_3_-I)_4_^42^ and **6** after recrystallization.

**Scheme 1:**
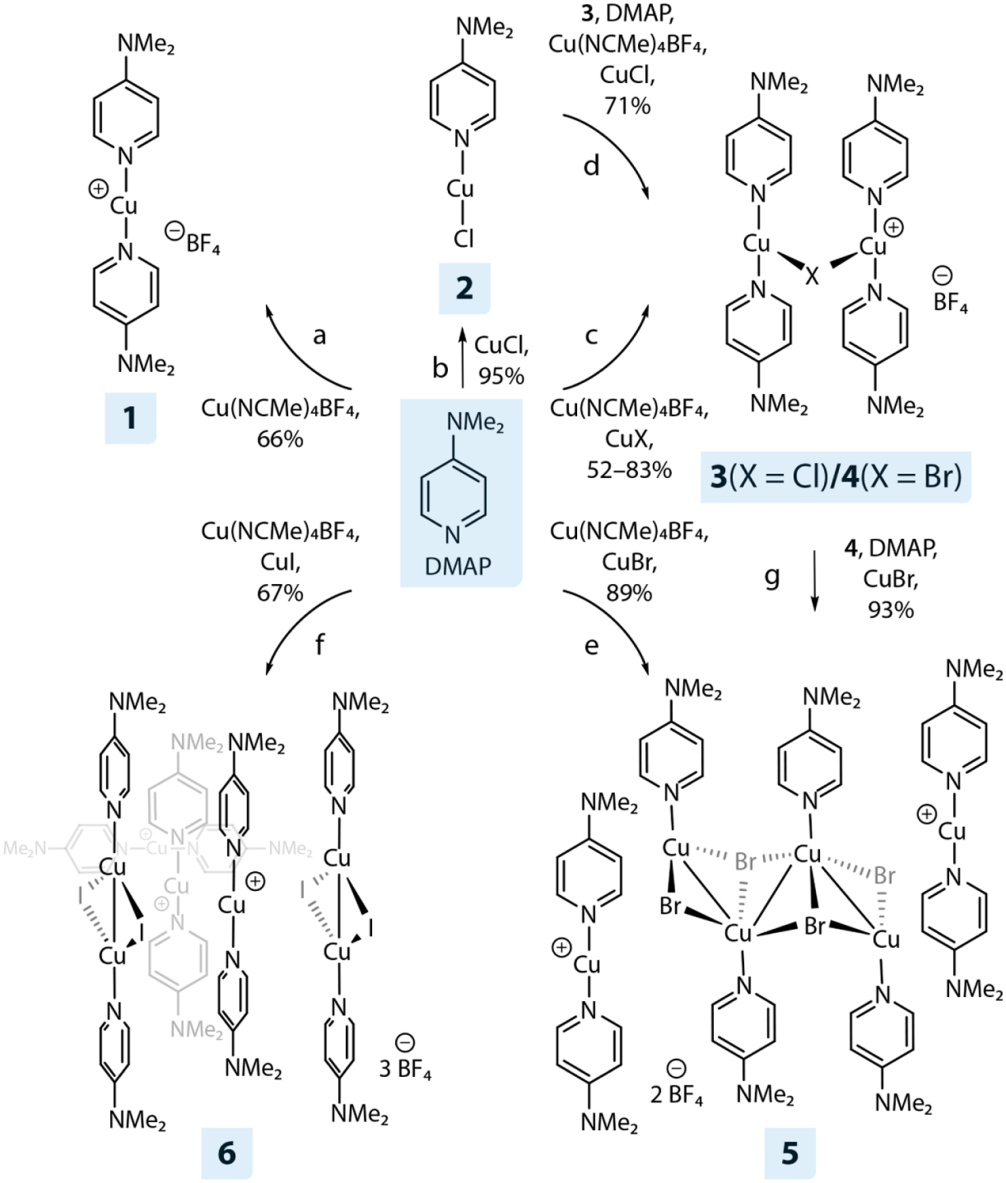
Synthetic Procedure to Mono- and Polynuclear DMAP-Cu­(I) Complexes **1**–**6** ^a^

a (a): 2 equiv of DMAP, 1 equiv of [Cu­(NCMe)_4_]­BF_4_, THF, DCM/CHX layering; (b): 1 equiv of DMAP, 1 equiv of CuCl, THF, DCM/CHX layering; (c): 4 equiv of DMAP, 1 equiv of CuX (X = Cl, Br), 1 equiv of [Cu­(NCMe)_4_]­BF_4_, THF, MeCN/Et_2_O gas-phase diffusion; (d): 1 equiv of **1** and **2**, 1 equiv of DMAP, DCM, MeCN/Et_2_O gas-phase diffusion; (e): 4 equiv of DMAP, 2 equiv of CuBr, 1 equiv of [Cu­(NCMe)_4_]­BF_4_, THF, DCM/Et_2_O, gas-phase diffusion (f): 10 equiv of DMAP, 4 equiv of CuI, 3 equiv of [Cu­(NCMe)_4_]­BF_4_, THF, DCM+toluene/pentane layering.

### Single-Crystal X-ray Diffraction (scXRD) Analysis

Crystalline complexes **1**–**6** were isolated as colorless or slightly yellow materials. Crystallographic data, refinement parameters, and selected structural bond lengths and angles of the molecular structures determined by scXRD analysis are summarized in Table S1 and Figures 1–3 and S7–11.

**Figure 1:**
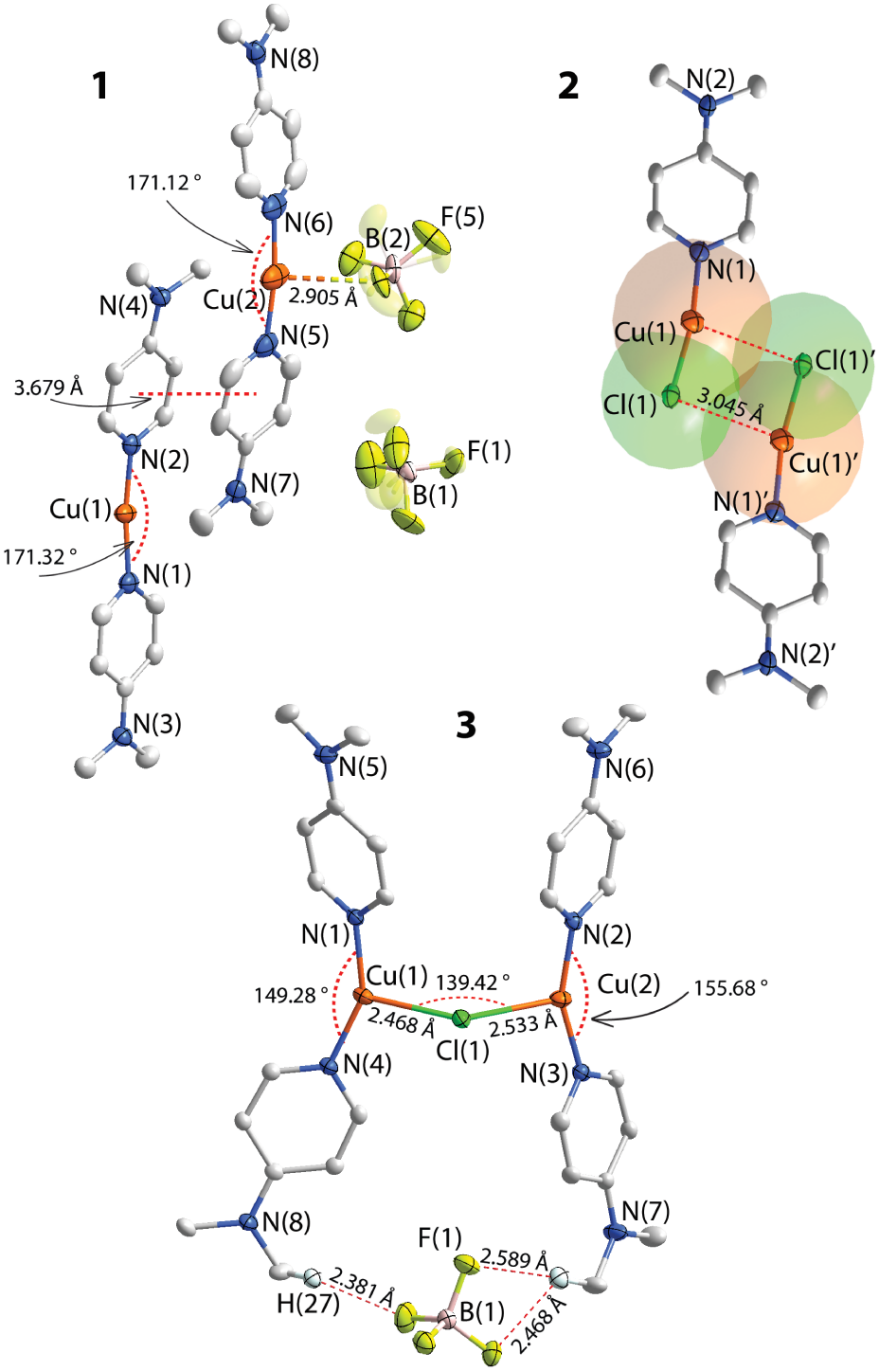
Molecular views of **1**–**3** (two independent molecules are depicted for **1**; the ellipsoids of Cu and Cl atoms are superimposed with space-filling spheres in **2**). Displacement ellipsoids are shown at the 50% probability level, hydrogen atoms are omitted for clarity.

The homoleptic complex **1** crystallizes in the space group *C*2/*c* with the asymmetric unit consisting of two [DMAP_2_Cu]­(BF_4_) units (Figure 1). In both independent cations, the metal atom holds two pyridine ligands and adopts a slightly distorted linear geometry with alike N–Cu–N angles of 171.12(16) and 171.32(14)°. DMAP ligands are arranged along one vertical axis and are barely tilted with the torsion angles of 4.09–11.21°. The flat planar “sticks” in the unit cell were found to form dimers presumably via very weak metallophilic Cu···Cu (3.049(10) Å) and π–π interactions, which also operate between these dimers, resulting in a center-to-center distance of 3.679(4) Å involving two dimethylaminopyridine fragments (Figures 1, S7). The structures with three- and four-coordinating modes of copper center among pyridine-copper­(I) adducts are more frequent representatives found in the CCDC database; however, examples of copper­(I) complexes with linear two-coordination geometries of transition metal are rare, yet some were reported back in the 1980–1990s.^74–77^ Although both counterions in **1** are orientationally disordered, the interaction network of the copper ions and the fluorine atoms are clearly established (Cu···F 2.561(92)–2.905(108) Å), as they are comparable or significantly shorter than the sum of the van der Waals radii for these atoms (vdW­(Cu+F) = 2.87 Å). In turn, the heteroleptic chloride species **2** in the solid state do not feature metallophilic contacts but are arranged in a dimer-like fashion as a result of highly asymmetric bridging coordination of the chlorides, leading to the weak intermolecular interactions indicated by Cu···Cl distances of 3.045(6) Å. The diagonal Cu···Cu separation is 3.577(4) Å, suggesting that there is no appreciable metal–metal bonding. These interactions evidently introduce additional strain as the N(1)–Cu(1)–Cl(1) angle (170.48(51)°) deviates from ideal linear geometry. The bond distances Cu–N in **1** and **2**, falling in the range of 1.878(30)–1.895(44) Å, are notably shorter than those found for tetracoordinated copper-halide complexes decorated with *N*-heteroaromatic ligands^42,78^ and thus indicate stronger interactions.

Within an asymmetric unit of cationic complex **3**, the two copper ions of the [DMAP_2_Cu]^+^ fragments are linked together by one bridging μ_2_-Cl ligand, forming the angle Cu(1)–Cl(1)–Cu(2) equal to 139.4(19)° (Figure 1). The significant change of the linear coordination environment of copper in **1** to a distorted trigonal geometry in **3** prevents the alignment of the dimethylaminopyridine ligands along one vertical axis. As depicted in Figure 1, the position of the BF_4_^–^ counterion participating in a network of C–H···F–B interactions of 2.38–2.59 Å (vdW­(H···F) = 2.67 Å), could be a key factor inducing the significant stretching/tightening of the molecule (the distance separation between the atoms N(5) and N(6) is 6.482(19) Å, whereas between the atoms N(7) and N(8) found to be as far as 8.695(19) Å). This asymmetry is clearly reflected in both the distances and the angles within the coordination sphere of each copper. For instance, the Cu(1)–Cl(1) bond is shorter than Cu(2)–Cl(1), 2.409(4) Å vs 2.533(4) Å, whereas Cu(1)–N­(1/4) contacts (1.929(14)–1.935(15) Å) are visibly longer than analogous distances for the second metallocenter Cu(2)–N­(2/3) (1.906(15)–1.911(14) Å). The deviation from the trigonal coordination geometry is more pronounced for Cu(2), which shows a more obtuse coordination angle N(2)–Cu(2)–N(3) of 155.68(59)° if compared to 149.28(58)° for the cognate angle around Cu(1). The tendency of copper­(I) to reach a tetrahedral coordination environment probably plays a non-negligible role in establishing extensive intermolecular contacts in the unit cell. The extensive C–H···π (DMAP) interactions and π···π stacking between DMAP moieties, further evidence that they likely act as driving forces in arranging two [DMAP_4_Cu_2_(μ_2_–Cl)]^+^ molecules into a dimer with a head-to-tail orientation and relatively long intermetallic Cu···Cu distances of 3.324(4) Å (Figure S7). Nevertheless, the counterion plays a crucial role in the observed packing mode: exchange of the tetrafluoroborate counterion to the chloride/nitrate and the use of *p*-aminopyridine instead of DMAP strongly impact the topology.^79,80^ The [^*p*‑amino^py_2_Cu^+^]­[ ^*p*‑amino^py_2_CuCl]­(Cl)/[^*p*‑amino^py_2_CuCl]­[^*p*‑amino^py_2_NO_3_] molecules, depicted on Figure S8, are cocrystallized stacked species with no obvious μ_2_-Cl bonding but instead a supportive network of C–H···Cl/NO_3_.^79,80^ (CSD entries: BEWHOD/NAXTAK)

The structural topology of [DMAP_4_Cu_2_(μ_2_–Br)]­BF_4_ (**4**) is similar to that of **3** but with a significantly smaller Cu(1)–Br(1)–Cu(2) angle of 69.5(45)° and a shorter metal–metal Cu(1)–Cu(2) distance of 3.038(17) Å (Figure 2). Two copper centers are nonequivalent and appear in a distorted trigonal geometry. In the same manner as in **3**, molecular asymmetry is driven by extensive C–H···F–B interactions. They additionally support two species in the solid-state forming dimers, which feature distinct π···π intra- and intermolecular contacts among three pyridine moieties (Figure 2, S9).

**Figure 2:**
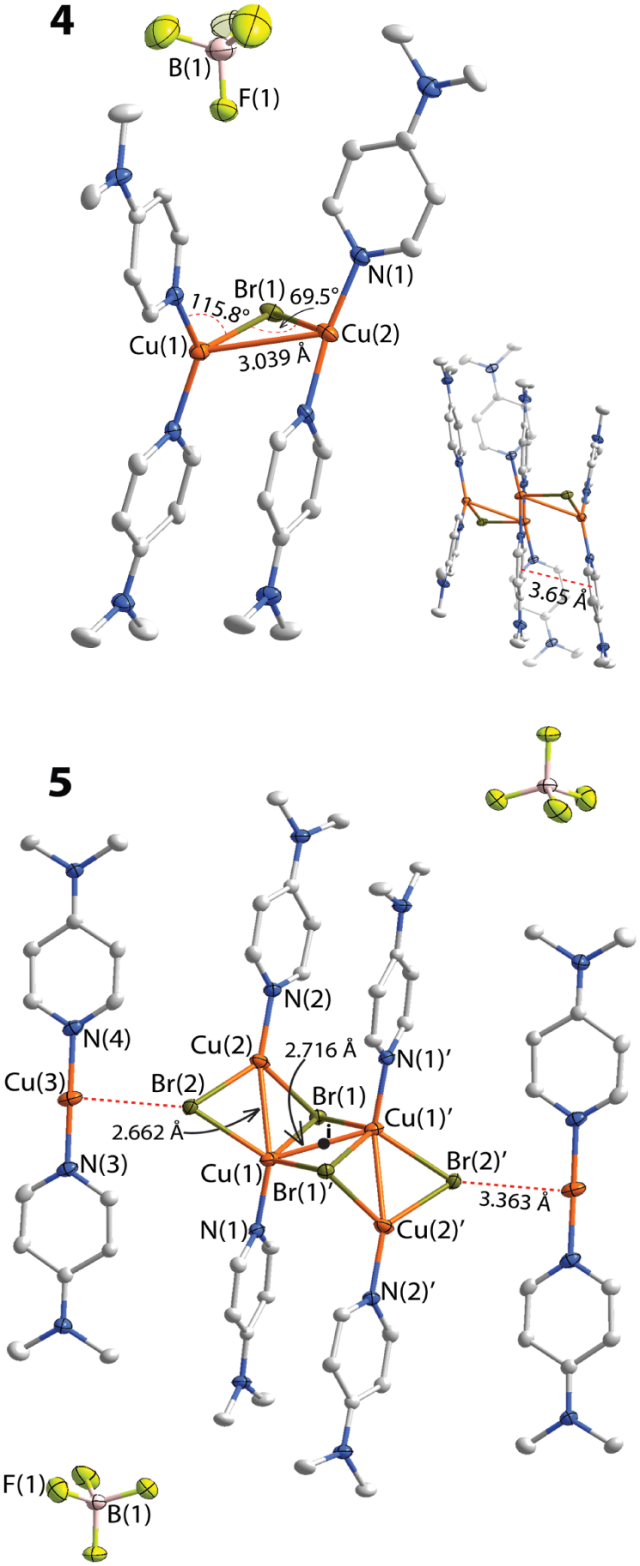
Molecular view of **4** and **5** (*i* – inversion center). The inset shows a dimer molecule of **4**. Displacement ellipsoids are shown at the 50% probability level, hydrogen atoms are omitted for clarity.

Similar bridging μ_2_-halide mode of Cu–X–Cu fragment, as observed in complexes **3** and **4**, has been reported in various 1D coordination polymers, metallacycles, and assemblies featuring a tetrahedral or slightly distorted tetrahedral geometry of the copper coordination environment.^81–86^ In particular, in dpmp-supported bi- and trinuclear cores (dpmp = bis­(diphenylphosphinomethyl)­phenylphosphine), removal or coordination of terminal ligands (e.g., MeCN, DCM or N-donors) was found to alter Cu···Cu distances and Cu–X–Cu angles, switching between coordination geometries and affecting metallophilic interactions. Complexes with chelating polyphosphines impose more rigid geometries, whereas assemblies built with monodentate N-donors display greater structural flexibility and wider angular distortions at the halide bridge. In this context, the bent Cu–X–Cu angle and asymmetric Cu–X bond metrics observed in **3** and **4** fall within the range reported for halide-bridged Cu­(I) units lacking strong geometric constraints, where secondary interactions and counterions further influence the coordination geometry. Interestingly, similar to **3** and **4**, the nuclearity control and dimerization in related systems have also been associated with π···π interactions, such as the intramolecular aromatic stacking (centroid–centroid distance ≈ 3.60 Å) reported for the symmetric [Cu_2_(dmp)_2_(PPh_3_)_2_(μ_2_-I)]^+^ (dmp = dimethylphenantroline) complex.^83^

As illustrated in Figures 2 and S10, the structure of the complex **5** is represented by a neutral tetrameric oligomer having [DMAP_4_Cu_4_(μ_2_-Br)_2_(μ_3_-Br)_2_] composition, which is cocrystallized with two linear cationic units of complex **1**. The aggregate is symmetrical due to the presence of an inversion center positioned in the midpoint of the “cut stair” motif (Figure 2). Cu–Br bond lengths with μ_2_-bridging bromide (2.351(4) Å, 2.467(4) Å) within the Cu_4_Br_4_ framework unit are systematically shorter than those with μ_3_-Br-ligand forming distorted pyramidal fragments Cu_3_Br (2.502­(4) Å, 2.552(3) Å and 2.584(3) Å). The intermetal Cu–Cu contacts (2.662(4) and 2.716(4) Å) are shorter than the sum of the van der Waals radii, *r*(vdW)­[Cu, Cu] = 2.80 Å). Other intramolecular parameters such as Br–Cu–Br, Cu–Br–Cu angles are comparable to these in congener (CuBrL)_4_ tetrameric stairlike species.^87–89^ Despite the distance between Cu(3) and Br(2) atoms (3.363(4) Å) is considerably longer than the corresponding *r*(vdW)­[Cu, Br] = 3.250 Å, the connectivity of the neighboring fragments is established through a network of noncovalent interactions. Analysis of the unit cell reveals that similarly to cationic species **1** and **3**, the [DMAP_2_Cu]^+^ fragments in **5** are arranged into a dimer (Figure S10). Furthermore, infinite translation of the structure demonstrates that dimeric species are forming a continuous 1D-sheet, which runs out along the *a*-axis of the unit cell and alternates with the layers of the neutral oligomeric DMAP_4_Cu_4_(μ_2_-Br)_2_(μ_3_-Br)_2_ “stairs” (Figure S10). To the best of our knowledge, such an unusual bilayer packing pattern of alternating cationic with neutral layers mainly induced by weak intermolecular interactions has not been described among copper­(I) complexes.

Multicomponent cocrystallization in the case of the **6** is even more complicated, and the general motif of the adduct is visualized in Figure 3. It was found in the *Pn* space group and consists of two types of constituents: cations of **1** and the neutral fragment DMAP_2_Cu_2_(μ_2_-I)_2_, in a 3:2 ratio. One linear [DMAP_2_Cu]^+^ “stick” (**1**) is clamped between two parallel rhomb-shaped dicopper species, which form a “sandwich” with intermolecular distances between the closest carbon atoms falling in the range 3.32–3.38 Å. The interfragment Cu–Cu contacts within this entity vary from 3.19 to 3.22 Å, likely indicating the absence of perceptible metallophilic bonding. Observed structural data, such as Cu–I, and Cu–Cu intramolecular distances and angles for the two rhomboidal Cu_2_(μ_2_-I)_2_ cores, are not exceptional and agree with the previously reported values for copper iodide relatives.^88,90^ Three central constituting fragments, despite having parallel orientation, feature significant deviation from linearity (159.6–167.4° ∠N^NMe2^–Cu–N^NMe2^) with respect to the DMAP-(M)-DMAP (M = Cu, Cu_2_I_2_) direction. This curving can be partially associated with the interactions between the neutral fragment [DMAP_2_Cu_2_I_2_] and the dimeric [DMAP_2_Cu]_2_^2+^ moiety, which comprise the I(4)–Cu(7) bonding distance (3.321(1) Å) and a set of noncovalent C–H···π and π···π contacts. Unlike **5**, weakly bonded [DMAP_2_Cu]^+^ fragments in **6** are oriented nearly perpendicular to each other, with torsion angles reaching 83.4–97.4°. Further analysis of the crystal packing reveals an ordered multilayered topology similar to that of **5** (Figure S11). Stacking of [DMAP_2_Cu_2_(μ_2_-I)_2_]­[DMAP_2_Cu]^+^ blocks via π···π forces (average 3.39 Å) forms a compact layer, which alternates with another layer composed of dicationic [DMAP_2_Cu]_2_^2+^ species (Figure S11). In turn, BF_4_^–^ counterions are located in the voids between the propeller-like [DMAP_2_Cu]_2_^2+^ fragments, holding these 1D layers via the network of C–H···F–B interactions.

**Figure 3:**
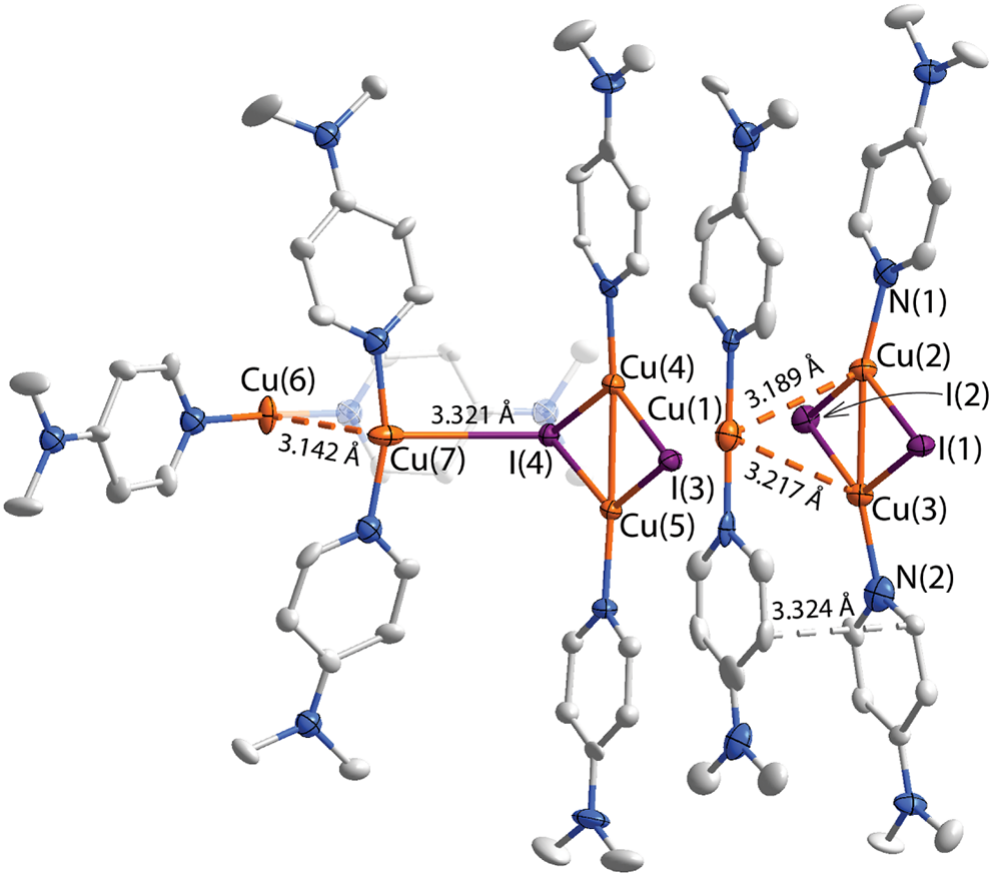
A molecular view of the aggregate **6**. Displacement ellipsoids are shown at the 50% probability level, hydrogen atoms and counterions are omitted for clarity.

The solution ^1^H NMR spectra of these compounds at ambient conditions feature two broad signals in the low field, which correspond to the aromatic DMAP protons and point to structural flexibility. Monitoring **1**–**6** at a low temperature (193 K) improves the sharpness of the resonance for **3**–**5**. However, for **1**-**2** and **6** the spectroscopic patterns suggest the presence of several type of species, indicating that dynamic processes are incompletely frozen (see ESI). Those processes likely refer to fluxional behavior arising from the labile coordination environment of Cu­(I) and likely include hindered rotation of the DMAP ligand, equilibria between mononuclear and weakly associated di- and polynuclear species, and coordination–decoordination of DMAP leading to fluctuations in the copper coordination number (e.g., transient DMAP_n_Cu^+^-type species, where n = 2–4). As dynamic behavior is not fully suppressed under the experimental conditions, the system represents an ensemble of interconverting structures, which can influence the observed broadness of the signals at low temperatures. It is plausible that the arrangement of compounds **5** and **6** in solution differs from that elucidated by scXRD and is represented by mixtures of DMAPCuX and [DMAP_2_Cu]­BF_4_ salts. Despite this fact, the shifted positions of the signals compared to free DMAP (δ = 8.21, 6.47 ppm), together with the NMR data obtained for other nuclei (^13^C, ^11^B, ^19^F and ^15^N) confirm coordination of the pyridine ligand to the copper ion.

Crystalline **1**–**6** samples are unstable toward moisture and oxygen, and their degradation in air occurs within several minutes to hours. The analytical purity of samples **1**–**6** was in complete agreement with calculated data and confirmed the crystallographically determined composition.

### Solid-State Photophysical Properties and TD-DFT Calculations

The complexes **1**–**6** exhibit no detectable photoluminescence in dichloromethane solution. This observation aligns with the NMR findings, indicating that these compounds undergo significant structural rearrangements, which favor nonradiative decay. Consequently, we focused on the analysis of the photophysical properties in the solid state. However, the very weak emission and low stability of **1** precluded a detailed investigation of that compound. While **2** features detectable luminescence, it was impossible to record the lifetimes of the excited state due to low emission intensity. The results of the steady-state and time-resolved luminescence studies, including photoluminescence quantum yield determination for **3**–**6** in the solid state, and theoretical calculations are summarized in Tables 1, S2–30 and depicted in Figures 4, 7, 8, S12–17.

**Table 1:** Selected Photophysical Properties of Crystalline **2**–**6** at 297 and 77 K

	T, K	λ_Exc_, nm	λ_Em_, nm	τ (A_*i*_), μs	τ_av_,^a^ μs	Φ	*k*_r_, 10^4^ s^–1^	*k*_nr_, 10^4^ s^–1^
**2** ^b^	297	287, 323	570	n.d.		<0.01		
	77	320	555	n.d.				
**3**	297	355	478	0.9 (39), 3.5 (48), 8.5 (13)	4.9	0.21	4.3	16.0
	77	335	475	12.9 (10), 23.5 (88), 48.9 (2)	23.7	0.94	4.0	0.3
**4**	297	325	530	10.5 (80), 16.5 (20)	12.2	0.13	1.1	7.1
	77	325	520	15.5 (17), 60.9 (73), 105.4 (10)	66.8	0.46	0.7	0.8
**5**	297	330	640	35.2 (26), 47.0 (74)	44.5	0.41	0.9	1.3
	77	325	530/640	23.9 (6), 124.7 (94)/127.0 (20), 589.6 (80)	123.4/581.1	0.43^c^		
**6**	297	330	572	1.4 (31), 3.6 (69)/2.8 (52), 11.3 (38), 61.0 (10)^d^	3.2/12.5^d^	0.24	7.3	23.0
	77	280, 315, 335	560	6.7 (90), 16.3 (10)	8.8	0.44	5.0	6.4

a Monitored at emission maxima unless specified; amplitude-weighted τ_av_ determined by equation ΣA_*i*_τ_*i*_/ΣA_*i*_, in which A_*i*_ = weight of component *i*; the *k*_r_ and *k*_nr_ values were estimated by Φ/τ_av_ and (1-Φ)/τ_av_, respectively.

b Poorly emissive at room temperature.

c Overall quantum efficiency at excitation 350 nm.

d Monitored at 750 nm; n.d. = not determined.

**Figure 4:**
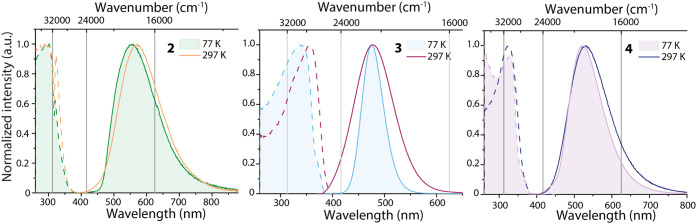
Normalized solid state excitation (dashed line) and emission (solid line) spectra of **2**–**4** at 297 and 77 K (filled); λ_exc_ = 330 nm (**2**), 350 nm (**3**–**4**); excitation spectra were monitored at the emission maxima.

Under photoexcitation (λ_exc_ = 320 nm), **2** exhibits yellow emission with a maximum at 570 nm and Φ < 0.01 (Figure 4). Upon cooling to 77 K, the broad unstructured band is slightly blue-shifted by ca. 480 cm^–1^ (15 nm), presumably due to enhanced environmental rigidity or minor differences in the intermolecular interactions at low temperature, as the emission onset and the excitation spectrum are also slightly shifted. The similarity of the full width at half-maximum (fwhm) at 297 and 77 K points to temperature-independent structural reorganization of the excited state. In comparison to the fluorescence of DMAP in the solid state (λ_fl_ = 334 nm),^91^ the emission maximum of **2** is significantly bathochromically shifted by ca. 12400 cm^–1^, which, in combination with the large apparent Stokes shift, suggests radiative decay from the triplet excited state T_1_; however, due to the low intensity of luminescence, no time-resolved data could be recorded. Since single-crystals of **2** comprise arrangements of (DMAP)­CuCl molecules in a dimer-like fashion, we performed TD-DFT calculations (Tables S6–S9) using the dimeric system **2** ^**d**^ shown in Figure 5. The calculations revealed the predominant MLCT character mixed with XLCT for the S_1_ excited state (Figure 5A), leading to a strong SOC (Table S20) mediated by the copper­(I) center, which enables efficient intersystem crossing (ISC) to the triplet manifold. The population of T_1_ is accompanied by noticeable molecular reorganization (Figure 5C), leading to asymmetric distortion of one of the (DMAP)­CuCl units by deviation of the dimethylamine group from the pyridine plane. Subsequently, the T_1_ state has a different orbital character than the S_1_ state. The lowest lying triplet is represented by Me_2_N (n)→pyridine π* intraligand (IL) CT with minor MLCT admixture (Figure 5B). Due to the distinct character of the T_1_ and S_1_ states, the singlet–triplet gap ΔE­(S_1_–T_1_) has a relatively large value of 580 meV (predicted at the optimized T_1_ state geometry), possibly ruling out a TADF emission channel. Although the spin–orbit coupling matrix element (SOCME) between T_1_–S_1_ states has a high value of 143.1 cm^–1^ (calculated at optimized T_1_ state geometry, see Table S21), the low S_1_ state oscillator strength f_osc_ (Table S8) likely suppresses phosphorescence rate *k*_r_(phosphorescence). Moreover, the coupling of T_1_ with singlet states is limited by substantial energy gaps. This effect is most pronounced for the S_2_ state, which possesses a considerable f_osc_, yet the calculated ΔE­(S_2_–T_1_) of 1.08 eV prevents its contribution to *k*_r_(phosphorescence), consistent with the observed low quantum yield of **2**. It is important to note that with the given methodological constraints, the performed calculations provide valuable qualitative insight. The results should be interpreted with caution, as the computed energies and state assignments are best regarded as indicative trends rather than definitive quantitative values due to applied restrains.

**Figure 5:**
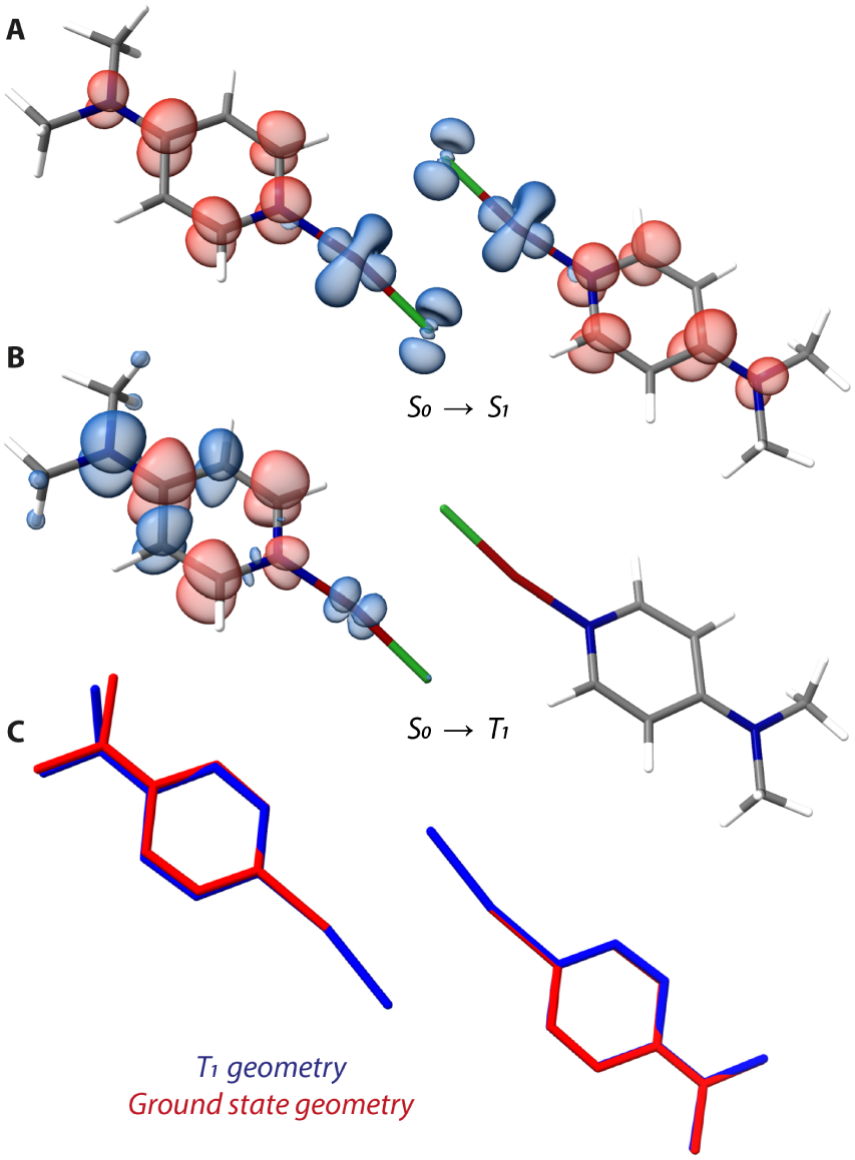
(A) Plots of TD-DFT calculated electron density differences for the S_0_→S_1_ and (B) S_0_→T_1_ transitions of **2^d^** at the ground state geometry (areas losing/gaining electron density are shown in blue/red). (C) Superposition of optimized ground (GS) and T_1_ state geometries of **2^d^**.

The room-temperature photoluminescence spectrum of crystalline **3** shows a broad structureless, but highly symmetrical profile maximized at 478 nm (λ_exc_ = 350 nm, Figure 4), which upon cooling to 77 K evolves an insignificant blue shift of 3 nm (λ_em_ = 475 nm). The emission profile of **3** narrowed by 47% (fwhm: 3960 cm^–1^ at 297 K vs 2105 cm^–1^ at 77 K) points to structural changes and enhanced rigidity of the molecular skeleton upon cooling. The emission spectra are highly symmetrical at both temperatures, which is rarely encountered for pure MLCT states. Instead, it suggests the T_1_ state has a mixed charge transfer character. The excited state is characterized by a short multiexponential decay with an average lifetime τ_av_ = 4.9 μs at 297 K and τ_av_ = 23.7 μs at 77 K (Table 1). In comparison to weakly luminescent **2**, the emission of halide-bridged **3** is considerably brighter (Φ = 0.21) and the quantum efficiency shows ∼4.5-fold increase almost to unity at cryogenic temperatures (Φ = 0.94 at 77 K). Thus, the radiative rate constants at 297 and 77 K were found to be nearly identical, *k*_r_ = 4.3 × 10^4^ s^–1^ and 4.0 × 10^4^ s^–1^, respectively, while nonradiative transitions are significantly suppressed upon cooling, *k*_nr_ = 16.0 × 10^4^ s^–1^ (297 K) and 0.3 × 10^4^ s^–1^ (77 K). This behavior indicates a pure phosphorescence mechanism for the excited-state radiative relaxation (T_1_→S_0_). The TD-DFT performed on an isolated molecule of **3**, with constraints on the Cu–Cl–Cu angle and the Cu–Cu distance, supports these conclusions. The S_1_ state has pronounced MLCT character with some XLCT admixture (Figures 6, S14), resulting in strongly SOC-coupled singlet and triplet excited states, and efficient ISC (Table S20). In contrast to **2**, considerable metal involvement and XLCT character are also maintained in the T_1_ state. The latter aligns well with the observed symmetrical emission profile, while the former likely accounts for the increased f_osc_ of the close-lying S_1_ (Table S12), which in turn enhances *k*_r_(phosphorescence) due to the large SOCME value up to 71.7 cm^–1^ (Table S21), thereby enabling the observed phosphorescence.

**Figure 6:**
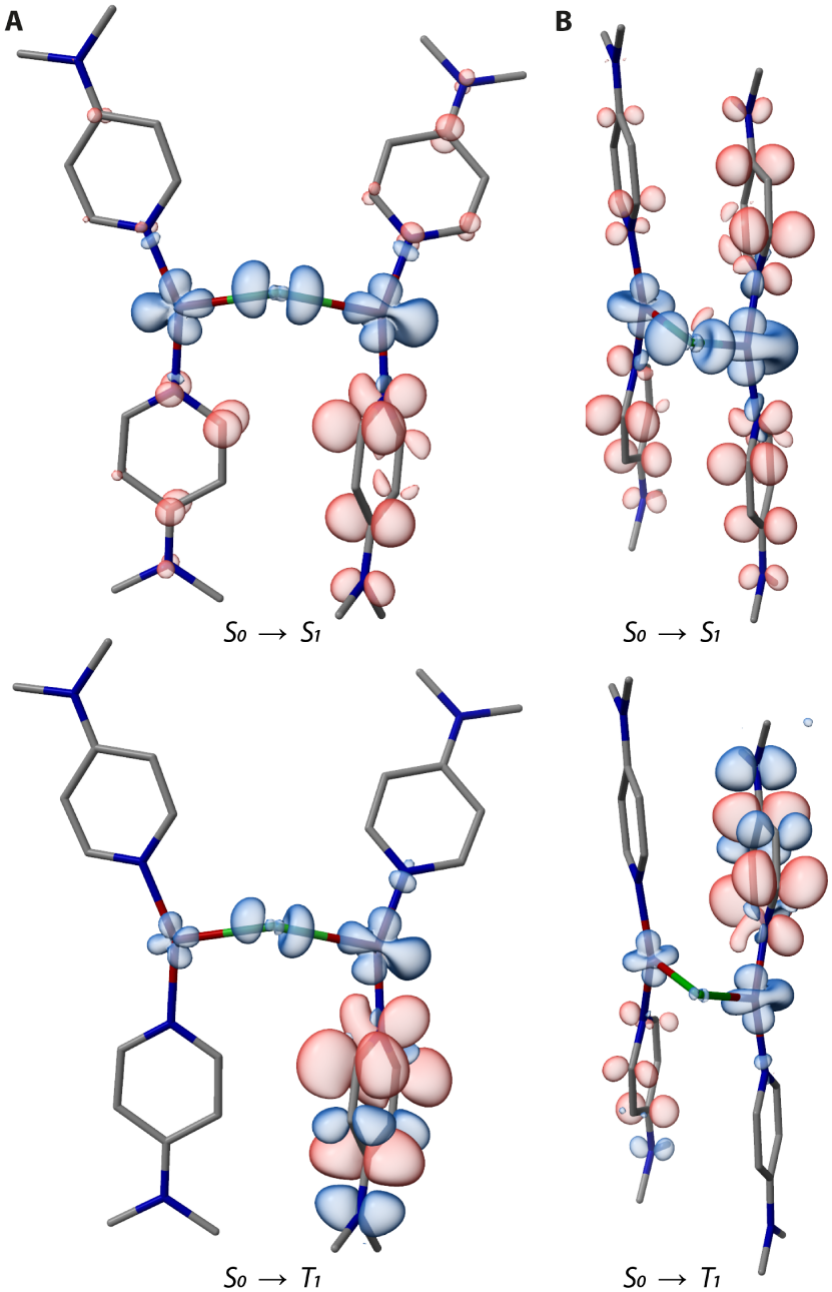
Plots of TD-DFT calculated electron density differences for the S_0_→S_1_ and S_0_→T_1_ transitions of **3** (A) and **4** (B) at the ground state geometry (areas losing/gaining electron density are shown in blue/red).

The emission of bromide-bridged **4** appears at lower energies (λ_em_ = 530 nm) than that of **3**, demonstrating that halide-dependent structural topologies and packing also significantly alter the photophysical characteristics (Figure 4). Unlike **3**, complex **4** features intramolecular metallophilic interactions within the Cu_2_Br core; hence, a bathochromic shift of the emission maximum would be expected.^92–94^ While the phosphorescence mechanism is maintained (τ_av_ = 12.2 μs, Φ = 0.12, *k*_r_ = 1.1 × 10^4^ s^–1^), the higher structural rigidity of **4** compared to **3**, arising from a more compact Cu_2_Br core due to the short separation between copper ions (Figures 2 and S9), suppresses vibrational deactivation, visibly reducing the nonradiative rate constant (*k*_nr_ = 16.0 × 10^4^ s^–1^ for **3** and 7.1 × 10^4^ s^–1^ for **4**). The fwhm values show minor changes (4264 cm^–1^ at 297 K vs 3765 cm^–1^ at 77 K), which support the above-mentioned rigidity argument. Cooling to 77 K induces a slight hypsochromic shift of 10 nm, and reduces *k*_nr_ by approximately 1 order of magnitude, while the phosphorescence rate remains in the same region. The S_1_ state has identical character to **3**; however, T_1_ is represented by MLCT with ILCT admixture, with negligible XLCT contribution (Figure 6). Since bromide is easier to oxidize compared to chloride, this surprising result probably originates from the close Cu–Cu contact, which shifts the metal orbitals energetically above those of Br. Such an insight into the structure–property relationship is important for controlled manipulation of the character of the excited states and related tuning of the photophysical behavior of CuX-based luminophores.^95,96^

At room temperature, **5** exhibits a broad emission profile with a maximum at 640 nm (Figure 7A), a quantum yield of 0.41, and an excited state lifetime of 44.5 μs, yielding a phosphorescence rate of up to 0.9 × 10^4^ s^–1^. The low-energy emission contrasts with the reported luminescence of (CuXL)_4_ staircase clusters, step-cubane tetramers, and polymers, which are associated with dominant XLCT excited states and higher emission energies (λ_em_ = 532–560 nm).^38,55,89,95–98^ The TD-DFT calculations show a mixed MLCT and cluster-centered (CC) character of the lowest lying triplet (Figure 7C); hence, the MLCT/CC states are probably thermodynamically lower in energy than the XLCT in the case of **5**.^38,97,98^ At 77 K, **5** exhibits dual emission with a low energy (LoE) and a high energy (HiE) band peaking at 640 and 530 nm, respectively, resulting in a significant emission color change as reflected in a shift of the CIE 1931 coordinates from red-orange (0.60, 0.40) to yellow-greenish (0.38, 0.52) (Figure 7A, D). The LoE emission resembles the room temperature (MLCT/CC) profile, while the TD-DFT revealed T_2_ state of ILCT nature with a small admixture of MLCT, which likely corresponds to the HiE band. Since cooling reduces the intensity of the LoE band and increases HiE emission, leading to an overall blue shift of ca. 3200 cm^–1^, these two states are apparently separated by a certain activation barrier. Moreover, the HiE band starts to develop at 140 K, indicating a low activation barrier of only a few kcal/mol (Figure 7B).

**Figure 7:**
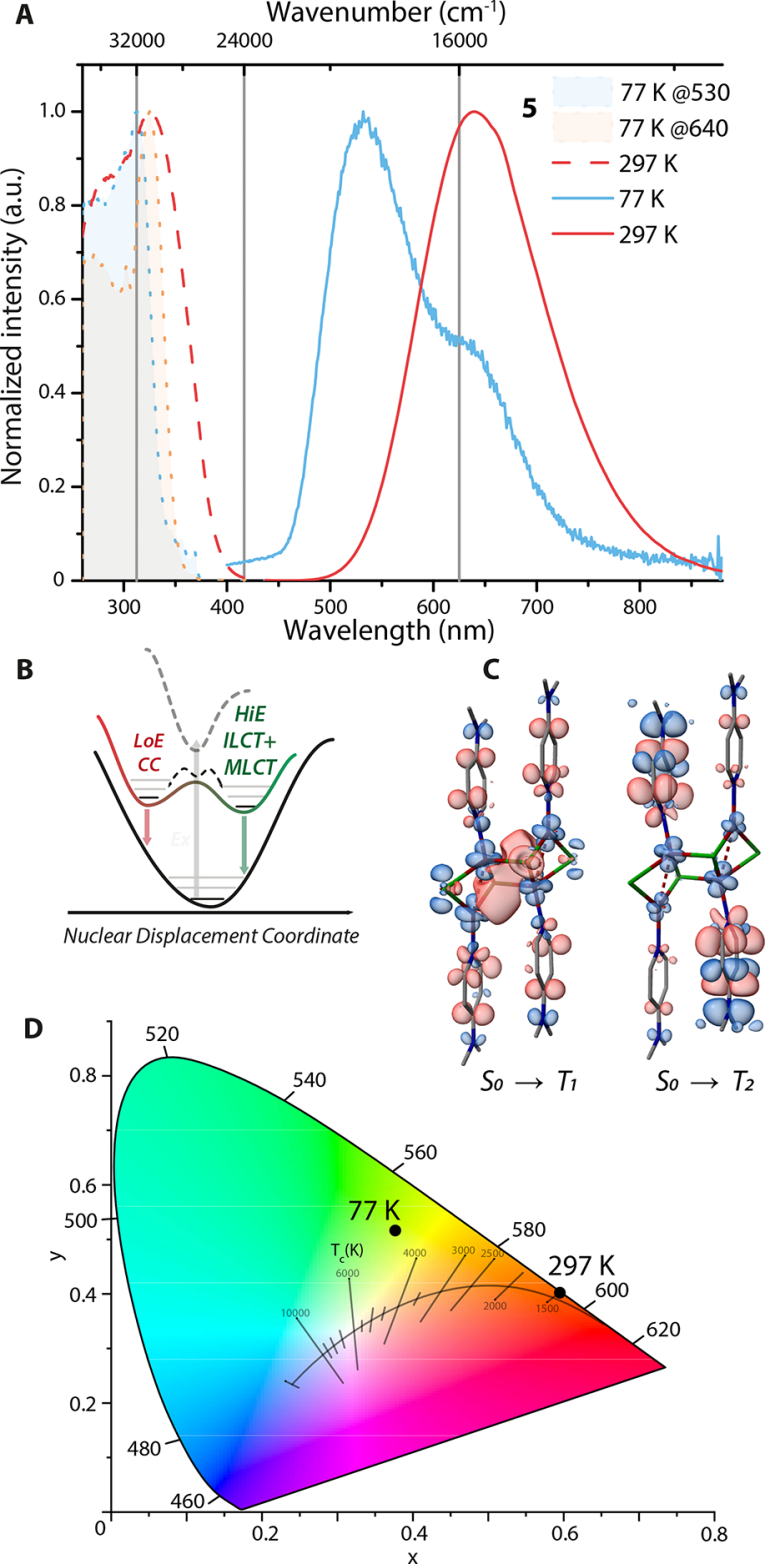
(A) Normalized solid state excitation and emission spectra (λ_exc_ = 365 nm) of **5** at 297 and 77 K. (B) Proposed relaxation mechanism for **5** at low temperature. (C) Plots of TD-DFT calculated electron density differences for the S_0_→T_1_ and S_0_→T_2_ transitions for fragment **5** (areas losing/gaining electron density are shown in blue/red). (D) CIE 1931 coordinates of emission **5** at 297 and 77 K.

Quantum yields at both temperatures remain nearly identical, i.e., 0.41 at 297 K and 0.43 at 77 K, while the average luminescence lifetime (λ_mon_ = 680 nm) is increased to 581.7 μs at 77 K. It is important to mention that the involvement of the (DMAP)_2_Cu^+^ stack dimers in the relaxation process may not be completely excluded, as the residual low-intensity emission observed at approximately 420 nm at 77 K could originate from ^3^π–π* transitions.

Compound **6** exhibits a structureless and broad room temperature emission spectrum centered at 572 nm (Figure 8), associated with a moderate quantum yield of 0.24 and the fastest radiative rate (*k*_r_ = 7.3 × 10^4^ s^–1^) within the studied series. This rapid radiative decay reflects a higher degree of SOC, facilitated by the heavy iodine atoms and rigid Cu_2_I_2_ core. The emission energy of **6** correlates well with the behavior of previously reported rhomboid Cu_2_I_2_ complexes.^99–101^ Notably, **6** contrasts with the very weak luminescence of related [Cu_2_I_2_L_4_] species.^102^ The asymmetry of the emission band at both 297 and 77 K (Figure 8) suggests a multiconfigurational emissive excited state, comprising XLCT, MLCT, and CC transitions. Excited state lifetimes recorded at different wavelengths (τ^570 nm^ = 3.2 μs/τ^750 nm^ = 12.5 μs) further corroborate this interpretation. Although it is difficult to describe **6** theoretically due to the very complex solid-state structure, molecular orbital analysis of the separated dimeric core provides additional insights, revealing low-lying molecular orbitals predominantly localized on the Cu_2_I_2_ core and DMAP ligand, respectively (Figure S17).

**Figure 8:**
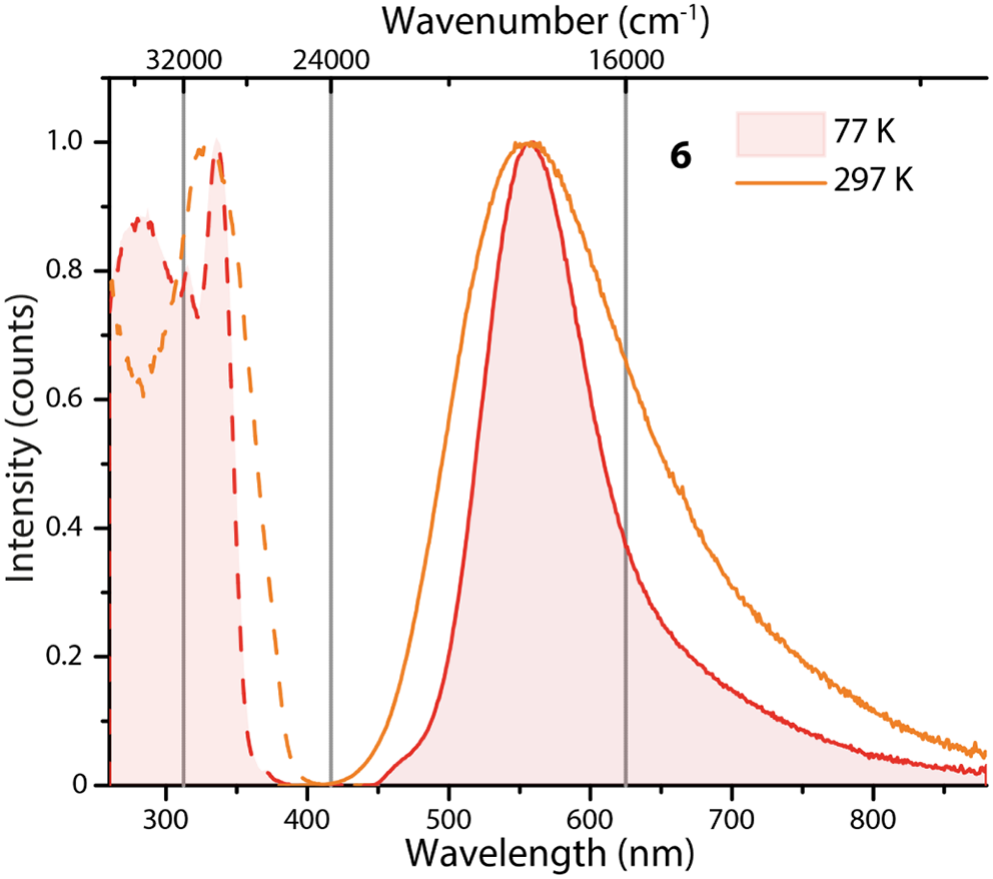
Normalized solid state excitation and emission spectra of **6** at 297 and 77 K (λ_exc_ = 365 nm).

## Conclusion

In summary, our study highlights the decisive role of crystallization conditions and the nature of halides in directing the structural and photophysical properties of copper­(I)-pyridine complexes. Through systematic variation of copper salts and stoichiometry, we isolated a family of compounds whose nuclearity and connectivity in the solid state range from mononuclear coordination cations to heptanuclear aggregates. Notable cases include previously unknown examples of species composed of cationic pyridine complexes and neutral halide clusters, expanding the known structural family of copper­(I)-pyridine halide chemistry. Single-crystal analyses revealed that subtle changes in halide bridging induce substantial alterations in packing and coordination. These topological variations are translated into wide-band phosphorescence across the visible spectrum, illustrating structural control over operative spin–orbit coupling constants and characters of low-lying excited states, as evidenced by TD-DFT calculations. Structural tuning via halide exchange appears to be a unique tool for suppressing nonradiative relaxation (*k*_nr_ = 16.0 × 10^4^ s^–1^ for **3** and 7.1 × 10^4^ s^–1^ for **4**). Achieving tunable emission, quantum yields up to 0.41, and radiative rates of *k*_r_ = 7.3 × 10^4^ s^–1^ at room temperature underscores the potential of these systems as efficient emitters derived from earth-abundant metals. Taken together, these findings establish crystallization-induced coordination diversity as a powerful design principle for developing next-generation copper­(I)-based photonic materials with controllable emission properties.

## Supplementary Material



## Notes

Additional experimental details, variable temperature NMR spectra, crystal data, structure refinement for **1**–**6**, selected molecular orbitals, additional spectroscopic data, and details of DFT/TD-DFT calculations (PDF)
