# Supplementary material for: Crystallization-Induced Coordination Diversity of Cu(I)-Pyridine Halide Complexes Resulting in Optical Tunability
Source: Inorg Chem. 2026 Mar 3;65(10):5507–21. doi: 10.1021/acs.inorgchem.5c05554 (PMC12997164; doi:10.1021/acs.inorgchem.5c05554)
Supplement: Supplementary file 1 [file ic5c05554_si_001.pdf]

Supporting Information for

Crystallization-Induced Coordination Diversity of Cu(I)-Pyridine Halide Complexes Resulting in Optical Tunability

Mariia Beliaeva,[a] Ondřej Mrózek,[b,c] Igor O. Koshevoy,[a] Andreas Steffen,[*b] Andrey Belyaev[*a, b]

[a] *Department of Chemistry and Sustainable Technology, University of Eastern Finland, Joensuu, 80101, Finland.*
[b] *Department of Chemistry and Chemical Biology, TU Dortmund University, 44227 Dortmund, Germany*
[c] *Institute of Inorganic Chemistry of the Czech Academy of Sciences, Husinec-Řež 250 68, Czech Republic*

E-mail: andreas.steffen@tu-dortmund.de; andrei.beliaev@uef.fi;

Table of Contents

Figures S1–6. Variable temperature ^{1}H and NMR spectra of **1–6**. S3

Table S1. Crystal data and structure refinement parameters for **1–6**. S10

Figures S7–11. Molecular views of the **1–6**. S11

Figure S12. Selected molecular orbitals of **2-6**. S14

Figures S13–17. Plots of TD-DFT calculated electron density differences for **1–6**. S14

Tables S2–19. Singlet/triplet excitations calculated for **1–6** in the ground (triplet) state geometry. S16

Table S20-21. Total values of SOCME between T_{1-5} and S_{0-5} states. S23

Tables S22-30. XYZ coordinates of optimized ground/triplet states of **1–6**. S25

NMR appendix S36

FT-IR appendix S45

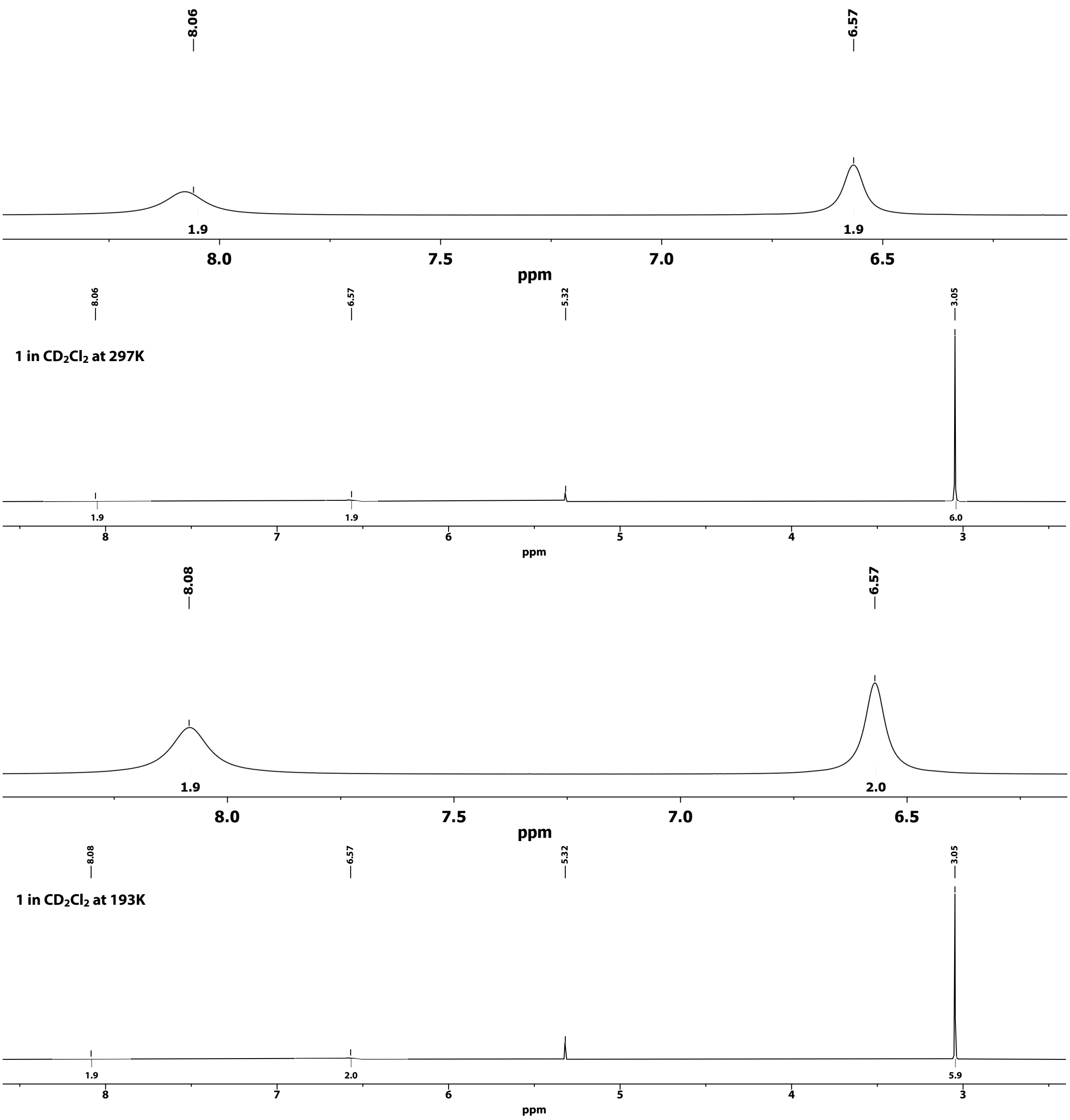

Figure S1. ^{1}H NMR spectra of the **1** in CD_2Cl_2 at 297K and 193K (magnified areas of aromatic protons are shown above the spectrum).

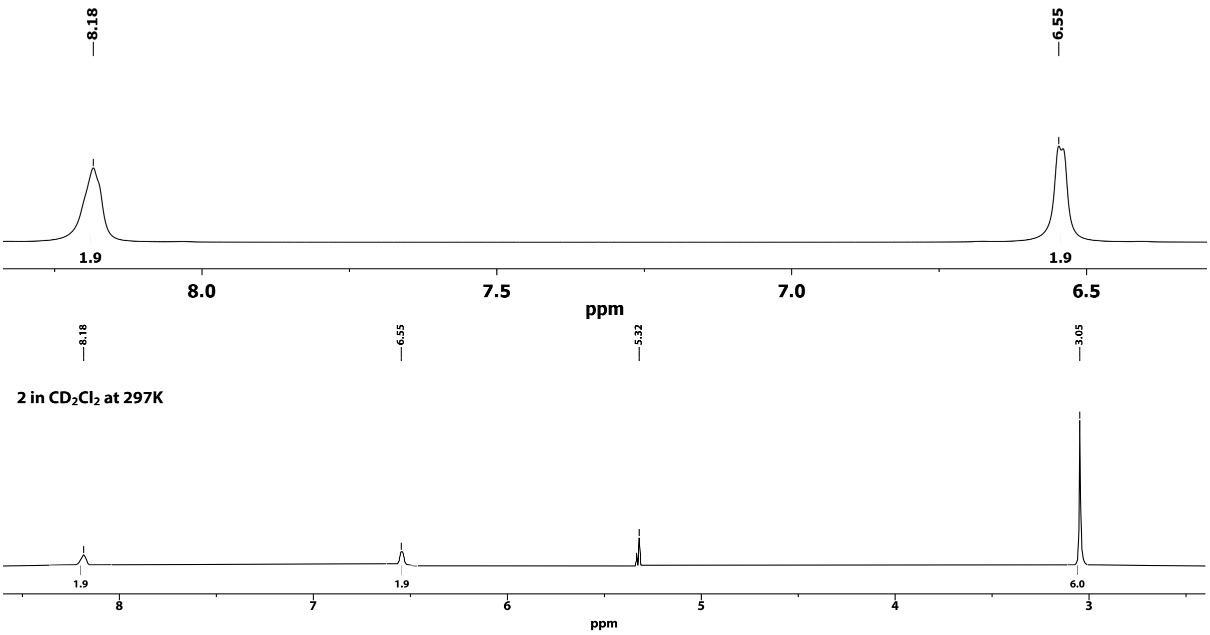

Figure S2. ^{1}H NMR spectra of the **2** in CD_2Cl_2 at 297K (Note: upon cooling to 193 K, precipitation occurs, magnified areas of aromatic protons are shown above the spectrum).

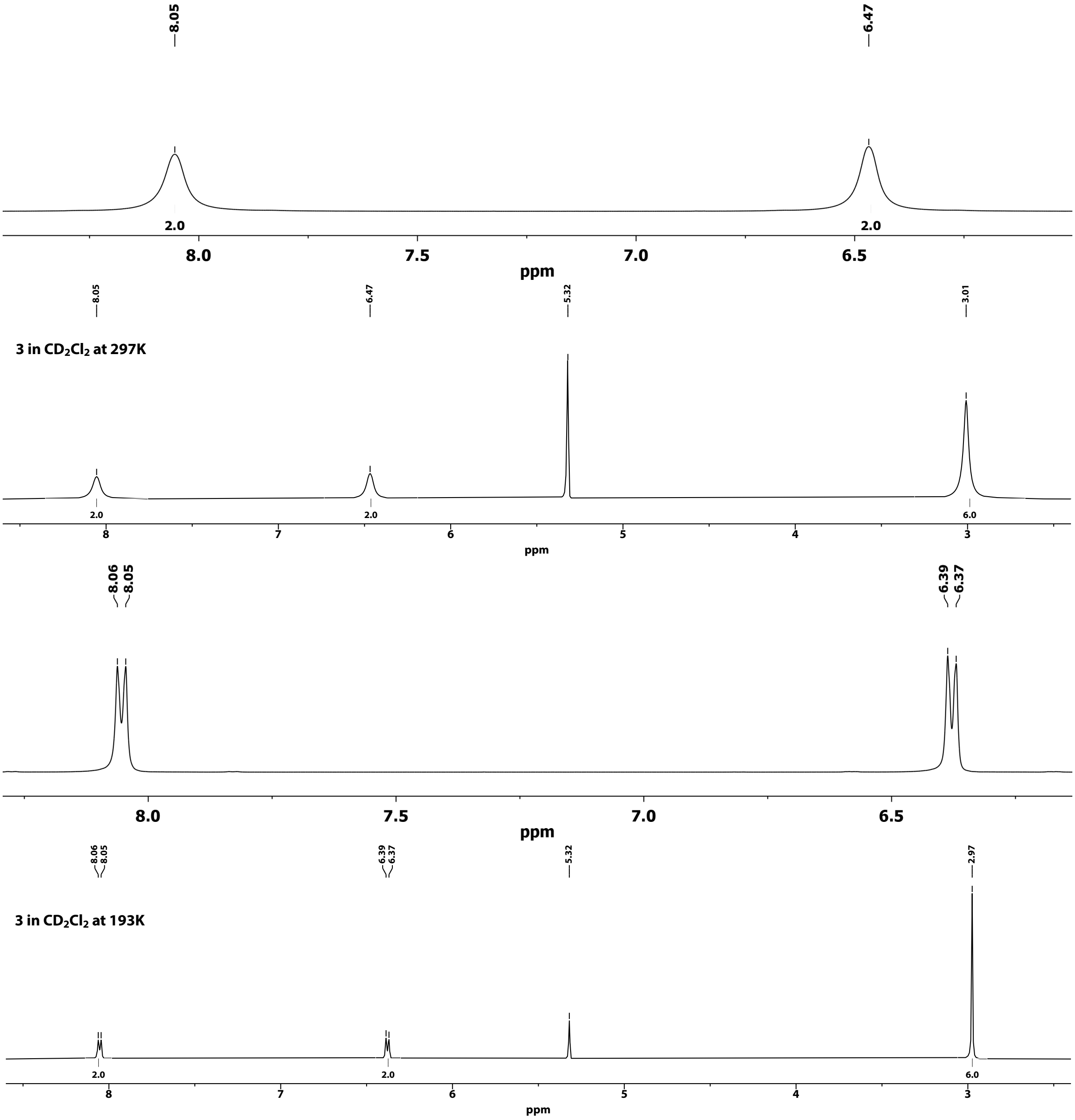

Figure S3. ^{1}H NMR spectra of the **3** in CD_2Cl_2 at 297K and 193K (magnified areas of aromatic protons are shown above the spectrum)..

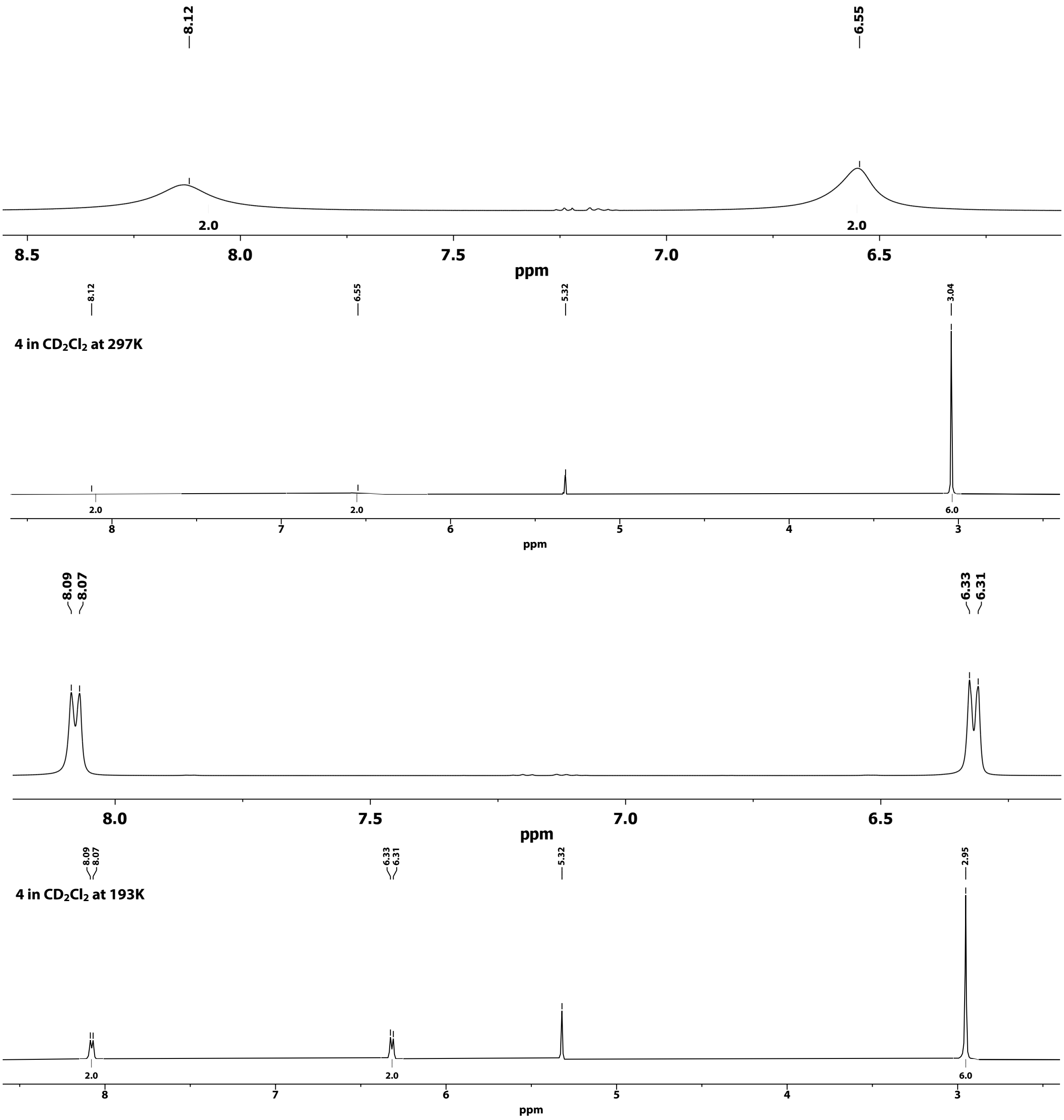

Figure S4. ^{1}H NMR spectra of the **4** in CD_2Cl_2 at 297K and 193K (magnified areas of aromatic protons are shown above the spectrum).

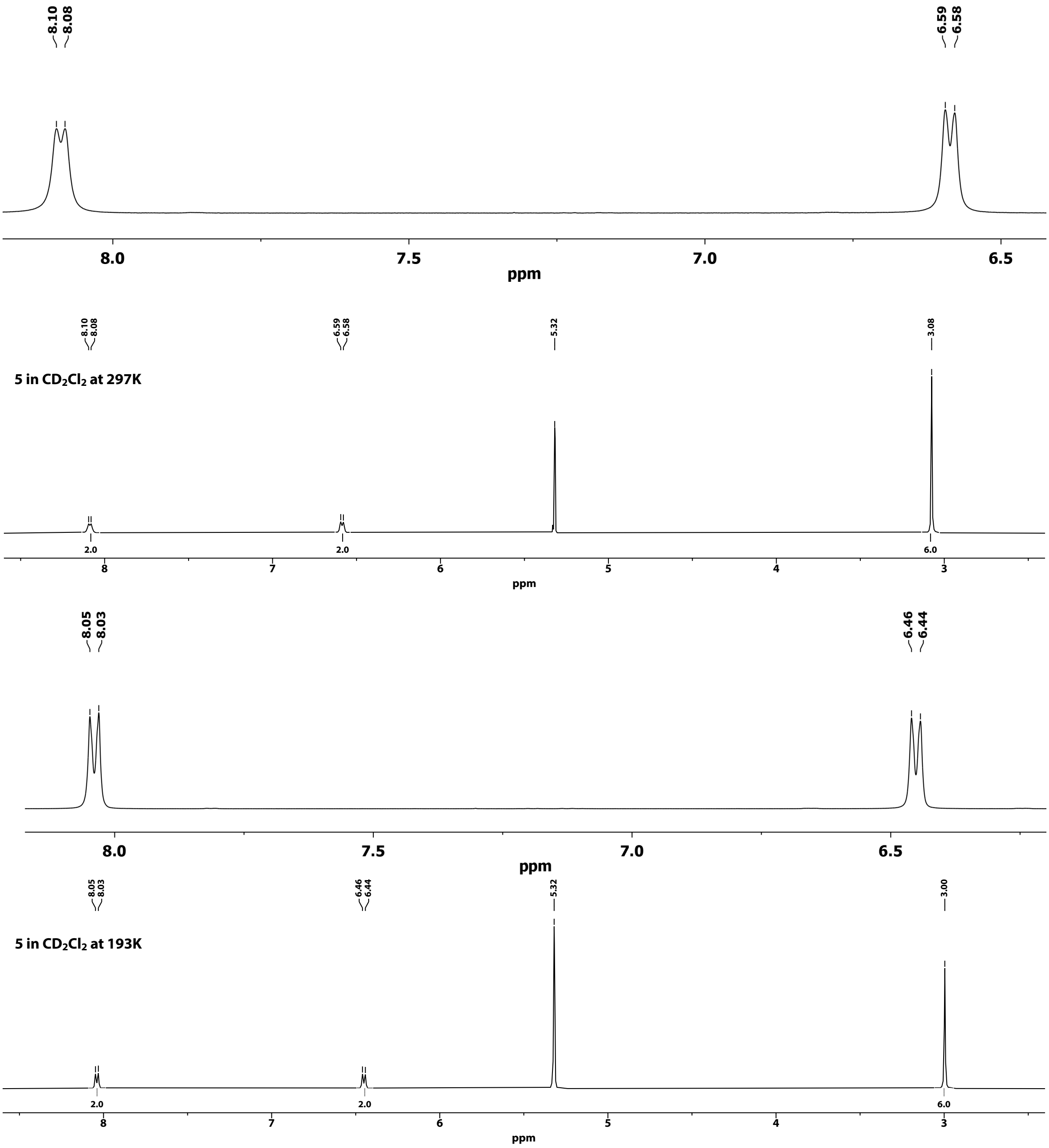

Figure S5. ^{1}H NMR spectra of the **5** in CD_2Cl_2 at 297K and 193K (magnified areas of aromatic protons are shown above the spectrum).

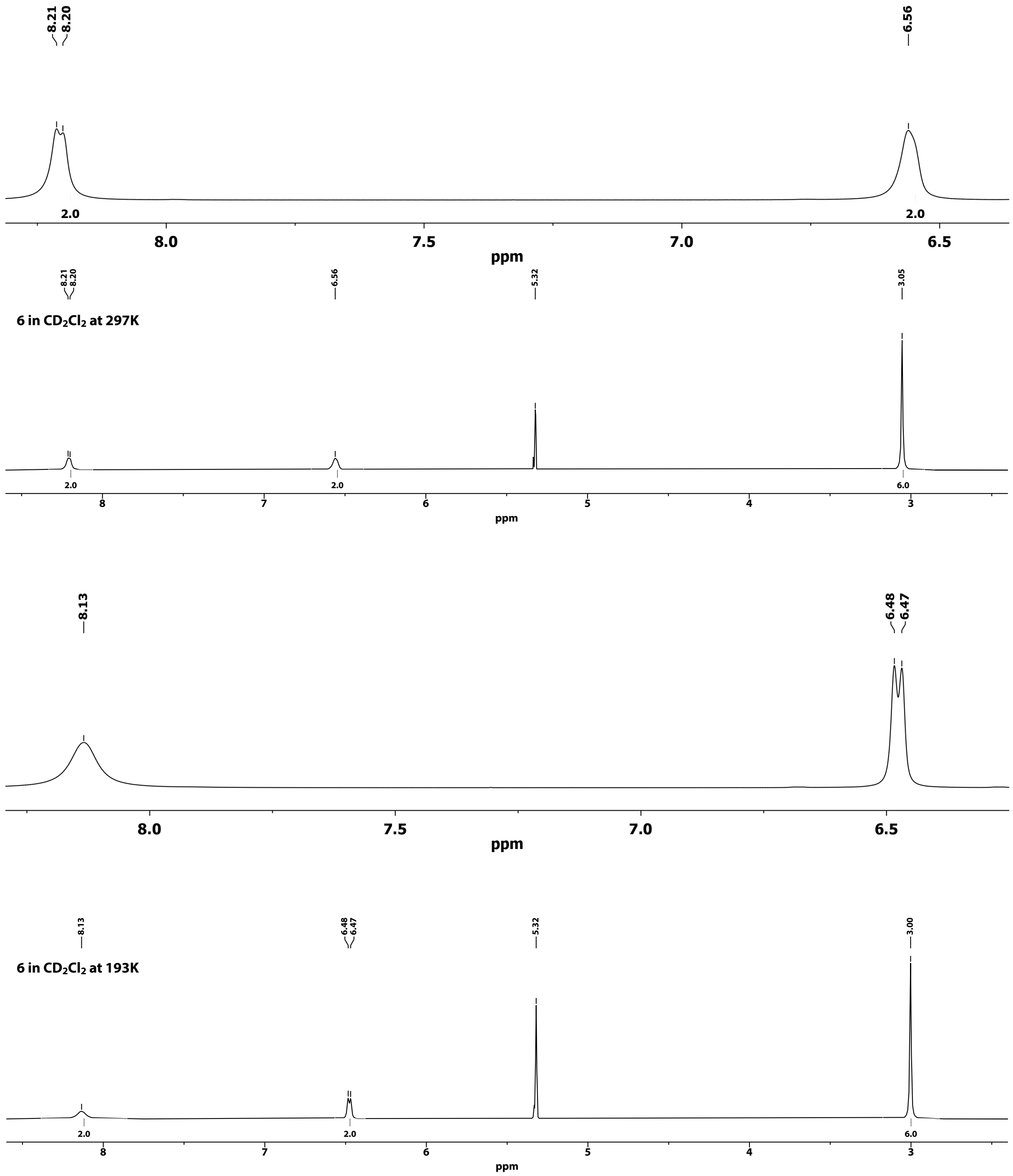

Figure S6. ^{1}H NMR spectra of the **6** in CD_2Cl_2 at 297K and 193K (magnified areas of aromatic protons are shown above the spectrum).

Table S1. Crystal data and structure refinement parameters for **1**–**6**.

Identification code	**1**	**2**	**3**	**4**	**5**	**6**
CCDC number	2416137	2416138	2416139	2416140	2416141	2416142
Empirical formula	$C_{14}H_{20}BCuF_4N_4$	$C_{28}H_{40}Cl_4Cu_4N_8$	$C_{28}H_{40}BClCu_2F_4N_8$	$C_{28}H_{40}BBrCu_2F_4N_8$	$C_{56}H_{80}B_2Br_4Cu_6F_8N_{16}$	$C_{70}H_{100}B_3Cu_7F_{12}I_4N_{20}$
Formula weight	394.69	884.64	738.02	782.48	1851.86	2434.50
Temperature/K	120.0	120	100.0	100.00	100.0	100.00
Crystal system	monoclinic	monoclinic	triclinic	monoclinic	triclinic	monoclinic
Space group	*C*2/*c*	*C*2/*c*	$P\bar{1}$	*P*2₁/*c*	$P\bar{1}$	*Pn*
a/Å	17.9185(7)	17.9428(9)	9.2543(2)	9.6175(10)	8.2688(4)	14.6023(7)
b/Å	11.4719(6)	7.4366(3)	13.3645(3)	19.6468(19)	12.9729(6)	14.5390(6)
c/Å	33.1007(18)	15.4421(8)	13.4546(3)	17.2691(16)	16.1383(8)	21.6069(11)
α/°	90	90	77.1970(10)	90	92.302(2)	90
β/°	103.043(2)	122.409(2)	88.9060(10)	93.003(4)	93.589(2)	102.904(2)
γ/°	90	90	81.2600(10)	90	99.043(2)	90
Volume/Å^3	6628.6(6)	1739.56(15)	1603.65(6)	3258.6(6)	1704.09(14)	4471.4(4)
Z	16	2	2	4	1	2
ρ_{calc}g/cm^3	1.582	1.689	1.528	1.595	1.805	1.808
μ/mm^{-1}	1.362	2.752	1.466	2.587	4.255	3.090
F(000)	3232.0	896.0	760.0	1592.0	924.0	2396.0
Crystal size/mm^3	0.091 × 0.085 × 0.032	0.35 × 0.12 × 0.05	0.23 × 0.12 × 0.08	0.12 × 0.11 × 0.07	0.12 × 0.06 × 0.051	0.14 × 0.11 × 0.08
Radiation	MoKα (λ = 0.71073)					
2Θ range for data collection/°	4.248 to 51.998	5.378 to 60.182	4.898 to 54.998	4.24 to 54.998	5.446 to 65.236	5.104 to 54
Index ranges	-22 ≤ h ≤ 21, -13 ≤ k ≤ 14, -40 ≤ l ≤ 40	-25 ≤ h ≤ 25, -10 ≤ k ≤ 10, -21 ≤ l ≤ 21	-12 ≤ h ≤ 12, -17 ≤ k ≤ 17, -17 ≤ l ≤ 15	-12 ≤ h ≤ 12, 0 ≤ k ≤ 25, 0 ≤ l ≤ 22	-12 ≤ h ≤ 11, -18 ≤ k ≤ 19, -24 ≤ l ≤ 24	-18 ≤ h ≤ 18, -18 ≤ k ≤ 18, -27 ≤ l ≤ 27
Reflections collected	33465	14319	47427	7473	42568	101746
Independent reflections	6494 [R_{int} = 0.0503, R_{sigma} = 0.0368]	2546 [R_{int} = 0.0311, R_{sigma} = 0.0243]	7353 [R_{int} = 0.0522, R_{sigma} = 0.0276]	7473 [R_{int} = 0.0654, R_{sigma} = 0.0510]	12358 [R_{int} = 0.0285, R_{sigma} = 0.0345]	18743 [R_{int} = 0.0803, R_{sigma} = 0.0637]
Data/restraints/parameters	6494/220/515	2546/0/102	7353/0/405	7473/97/406	12358/0/423	18743/117/1059
Goodness-of-fit on F^2	1.024	1.047	1.037	1.113	1.010	1.046
Final R indexes [I>=2σ (I)]	R_1 = 0.0624, wR_2 = 0.1585	R_1 = 0.0253, wR_2 = 0.0657	R_1 = 0.0242, wR_2 = 0.0602	R1 = 0.0884, wR2 = 0.2672	R_1 = 0.0272, wR_2 = 0.0525	R_1 = 0.0610, wR_2 = 0.1531
Final R indexes [all data]	R_1 = 0.0799, wR_2 = 0.1700	R_1 = 0.0334, wR_2 = 0.0690	R_1 = 0.0319, wR_2 = 0.0624	R1 = 0.0986, wR2 = 0.2822	R_1 = 0.0409, wR_2 = 0.0563	R_1 = 0.0762, wR_2 = 0.1617
Largest diff. peak/hole / e Å^{-3}	2.12/-1.37	0.34/-0.38	0.31/-0.29	2.15/-1.40	0.57/-0.66	2.64/-2.80

[(a)] $R_1 = \sum||F_o|-|F_c||/\sum|F_o|$; $wR_2 = [\sum[w(F_o^2-F_c^2)^2/\sum[(wF_o^2)^2]]^{1/2}$; $w = 1/[\sigma^2(F_o^2)+(aP)^2+bP]$, where $P = (F_o^2+2F_c^2)/3$ [(b)] GooF = S = $[[\sum w(F_o^2-F_c^2)^2]/(m-n)]^{1/2}$, where m = number of reflexes and n = number of parameters

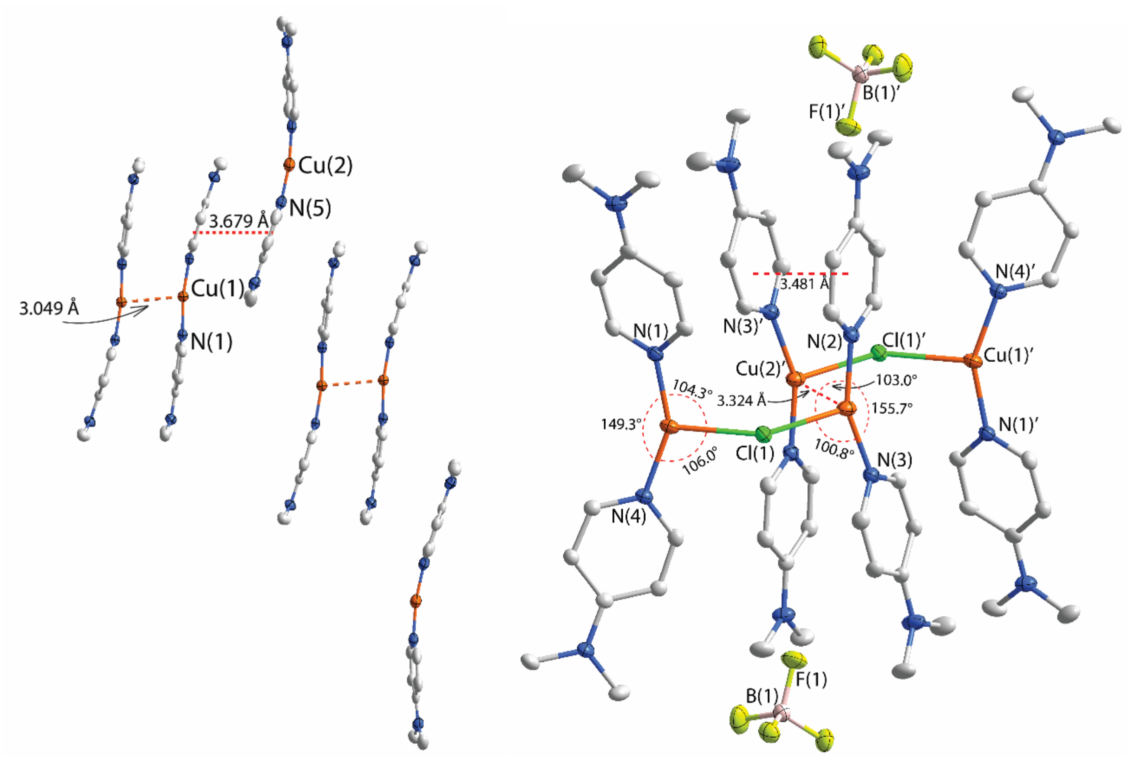

Figure S7. Molecular views of the dimeric molecules of the **1** (left) and **3** (right).

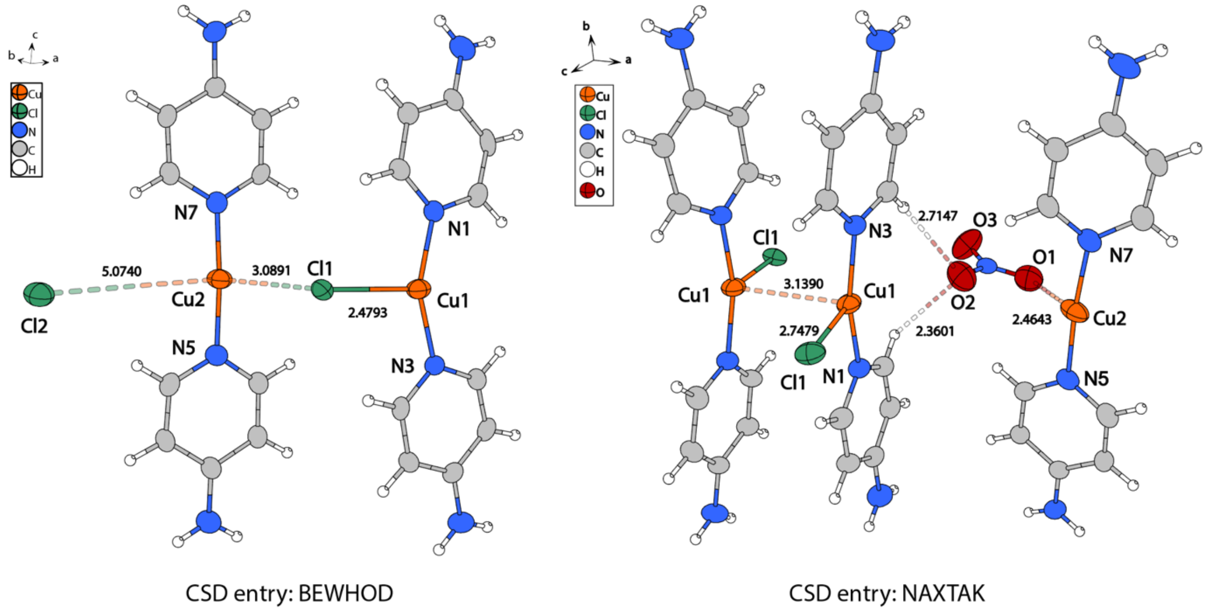

Figure S8. Molecular views of the [$^{p\text{-amino}}$py$_2$Cu$^+$][$^{p\text{-amino}}$py$_2$CuCl](Cl)(left) and [$^{p\text{-amino}}$py$_2$CuCl][$^{p\text{-amino}}$py$_2$NO$_3$] (right) molecules.

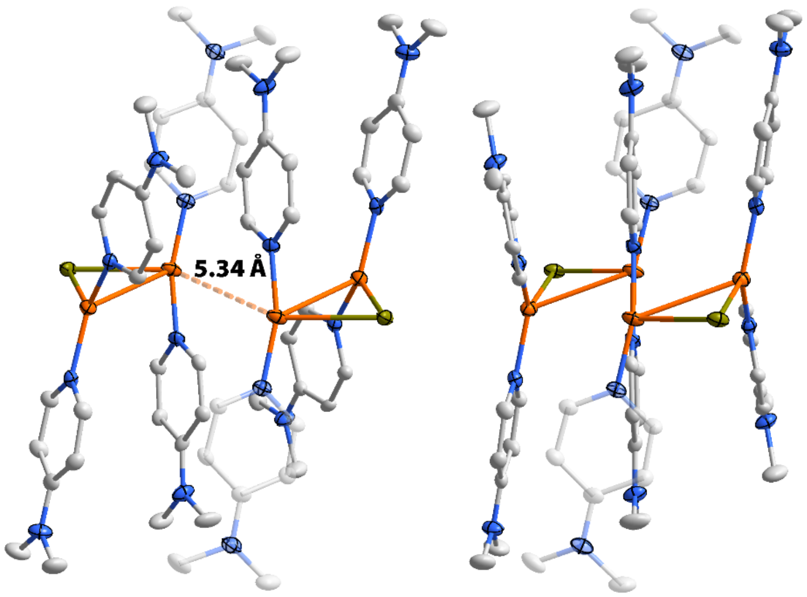

Figure S9. Molecular view of dimeric molecules of **4**. Thermal ellipsoids are shown at the 50% probability level, hydrogen atoms are omitted for clarity.

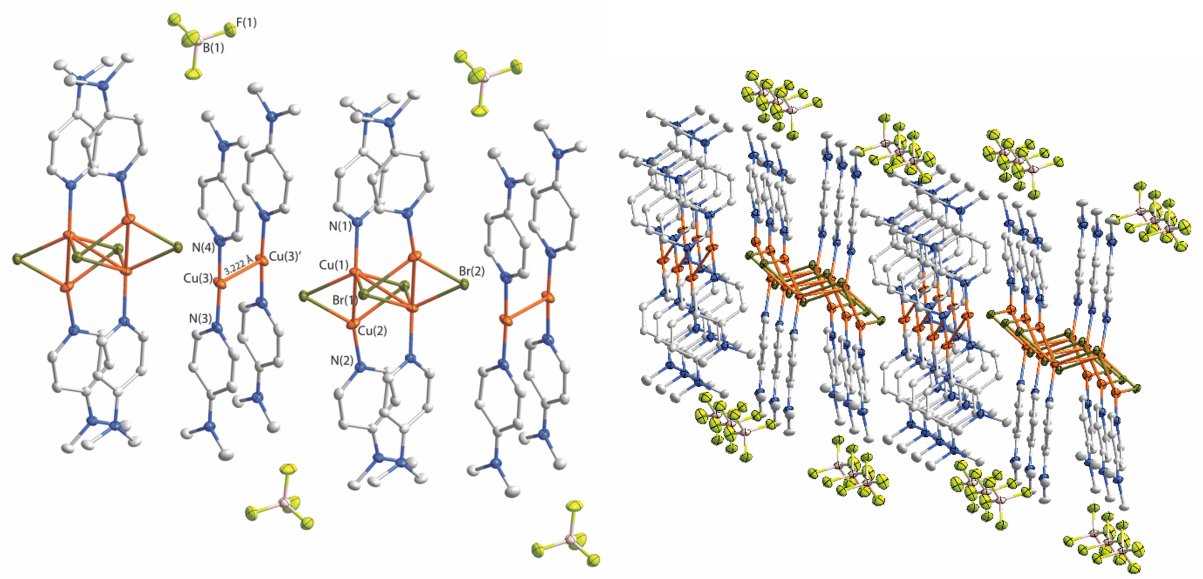

Figure S10. Fragments of crystal packing of **5** showing intermolecular interactions.

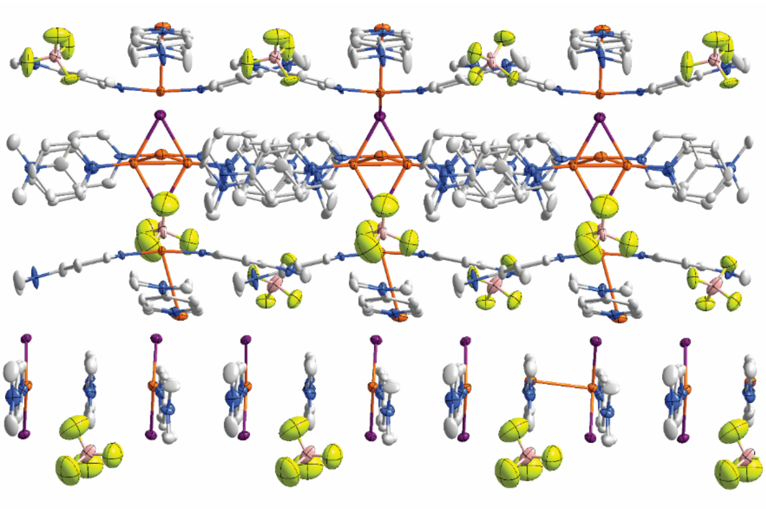

Figure S11. Fragment of crystal packing of **6**.

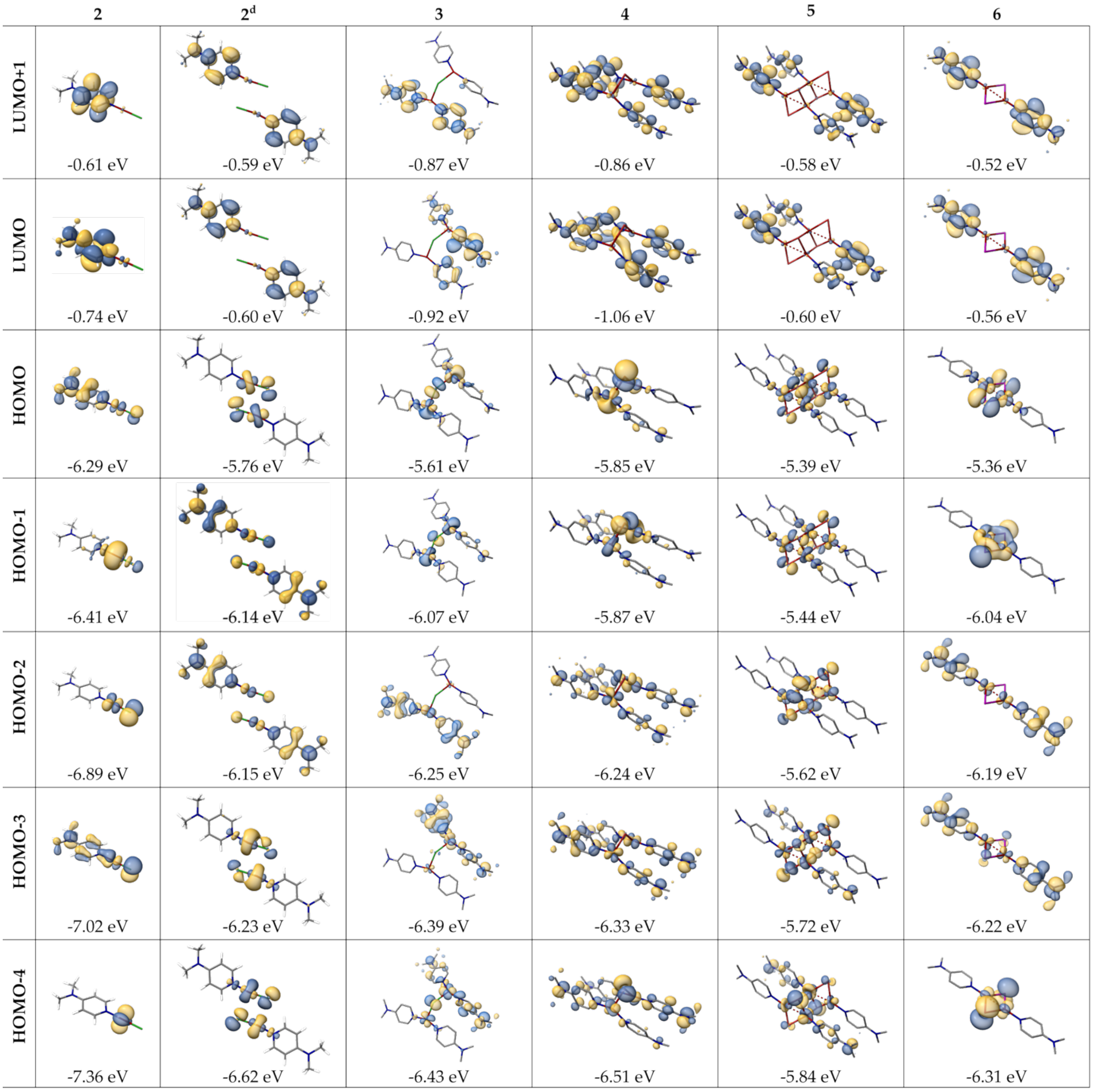

Figure S12. Selected molecular orbitals of **2-6** (isovalue ±0.03).

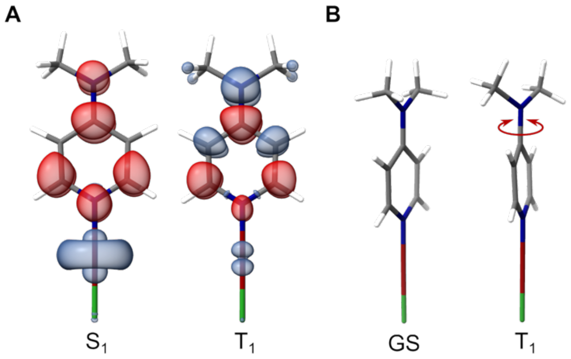

Figure S13. Plots of TD-DFT calculated electron density differences (isovalue ±0.003, areas losing/gaining electron density are shown in green/red) for the $S_0 \rightarrow S_1$ and $S_0 \rightarrow T_1$ transitions of linear form of **2** (**A**), Ground state a T_1-state geometries of **2** (**B**).

3

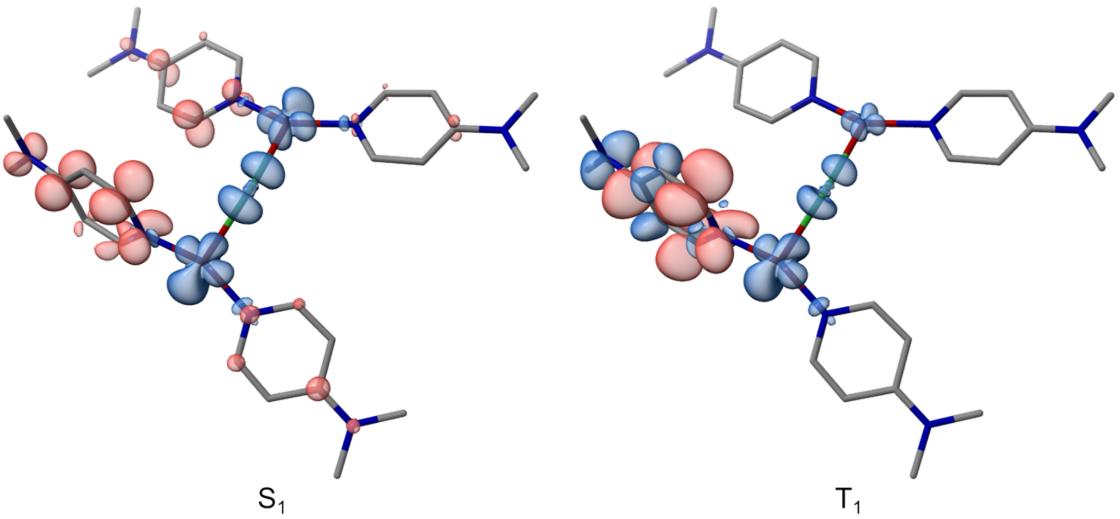

Figure S14. Plots of TD-DFT calculated electron density differences (isovalue ±0.003, areas losing/gaining electron density are shown in green/red) for the $S_0 \rightarrow S_1$ and $S_0 \rightarrow T_1$ transitions of linear form of **3**.

4

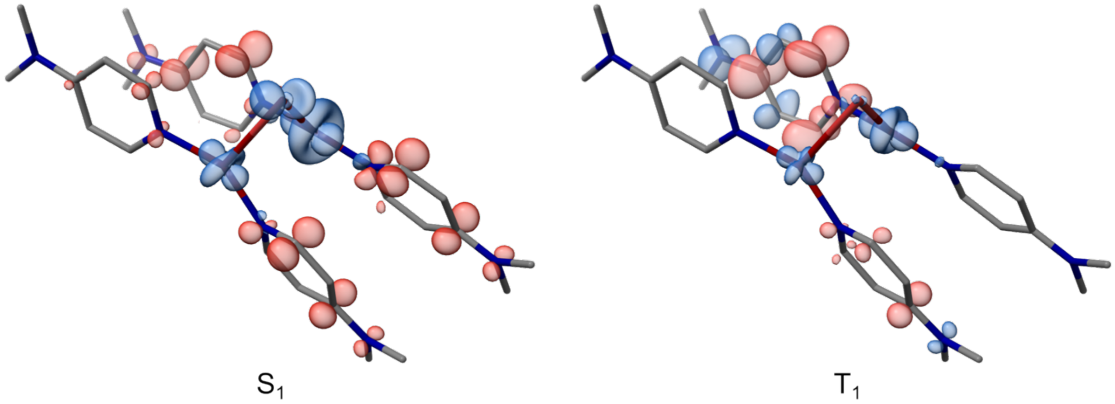

Figure S15. Plots of TD-DFT calculated electron density differences (isovalue ±0.003, areas losing/gaining electron density are shown in green/red) for the $S_0 \rightarrow S_1$ and $S_0 \rightarrow T_1$ transitions of linear form of **4**.

5

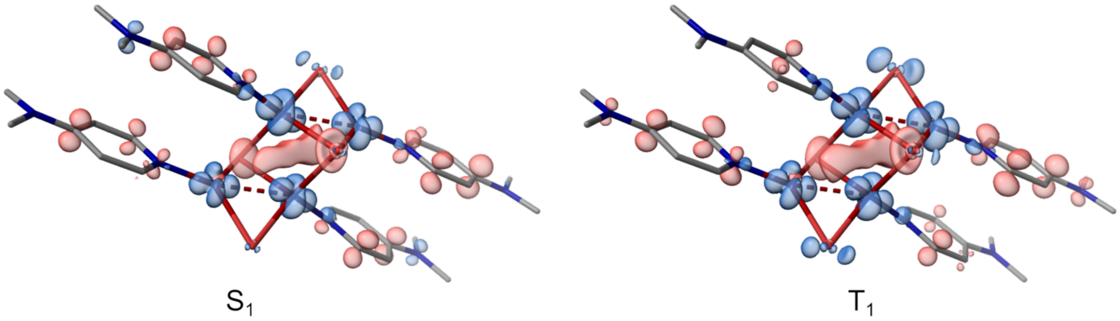

Figure S16. Plots of TD-DFT calculated electron density differences (isovalue ±0.003, areas losing/gaining electron density are shown in green/red) for the $S_0 \rightarrow S_1$ and $S_0 \rightarrow T_1$ transitions of linear form of **5**.

6

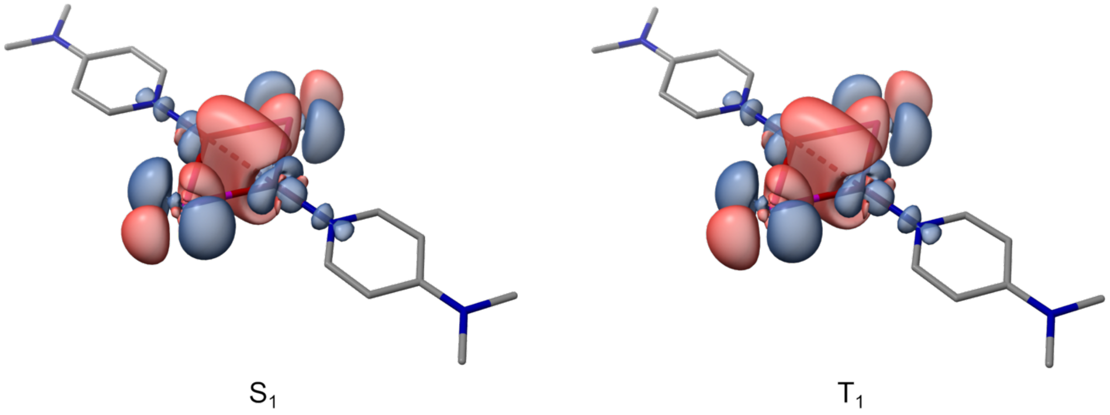

Figure S17. Plots of TD-DFT calculated electron density differences (isovalue ±0.001, areas losing/gaining electron density are shown in green/red) for the $S_0 \rightarrow S_1$ and $S_0 \rightarrow T_1$ transitions of the linear form of **6**.

Table S2. Singlet excitations calculated for **2** in the ground state geometry.

State	Energy [eV]	Energy [cm^{-1}]	λ [nm]	f_{osc}
1	4.53	36503.3	273.9	3.425E-03
2	4.70	37903.3	263.8	3.127E-02
3	4.73	38161.1	262.0	3.398E-06
4	4.83	38984.7	256.5	4.613E-01
5	5.10	41124.7	243.2	8.670E-07
6	5.12	41302.5	242.1	5.371E-03
7	5.33	42981.6	232.7	1.102E-02
8	5.34	43045.5	232.3	1.286E-05
9	5.44	43907.3	227.8	1.175E-04
10	5.46	44006.9	227.2	2.972E-04
11	5.46	44076.5	226.9	3.658E-03
12	5.51	44476.5	224.8	2.326E-01
13	5.65	45562.1	219.5	3.583E-03
14	5.71	46023.1	217.3	1.460E-07
15	5.71	46084.3	217.0	3.336E-06
16	5.72	46116.1	216.8	2.833E-03
17	5.89	47502.8	210.5	3.571E-02
18	6.16	49711.4	201.2	6.335E-04
19	6.37	51353.8	194.7	3.309E-02
20	6.53	52657.9	189.9	2.662E-01

Table S3. Triplet excitations calculated for **2** in the ground state geometry.

State	Energy [eV]	Energy [cm^{-1}]	λ [nm]
1	3.64	29398.0	340.2
2	4.01	32374.4	308.9
3	4.35	35090.8	285.0
4	4.42	35635.9	280.6

5	4.62	37263.4	268.4
6	4.71	37975.7	263.3
7	4.88	39360.6	254.1
8	5.04	40660.4	245.9
9	5.05	40738.1	245.5
10	5.07	40889.0	244.6
11	5.07	40922.5	244.4
12	5.25	42343.1	236.2
13	5.25	42345.0	236.2
14	5.33	42955.9	232.8
15	5.35	43138.0	231.8
16	5.41	43627.6	229.2
17	5.44	43885.1	227.9
18	5.70	45993.6	217.4
19	5.71	46033.0	217.2
20	6.08	49041.1	203.9

Table S4. Singlet excitations calculated for **2** in the T_1 geometry.

State	Energy [eV]	Energy [cm^{-1}]	λ [nm]	f_{osc}
1	3.97	32005.7	312.4	4.377E-03
2	4.15	33501.7	298.5	5.888E-01
3	4.57	36847.7	271.4	2.312E-04
4	4.60	37134.6	269.3	9.449E-02
5	4.78	38532.4	259.5	9.272E-04

Table S5. Triplet excitations calculated for **2** in the T_1 geometry.

State	Energy [eV]	Energy [cm^{-1}]	λ [nm]
1	3.03	24461.8	408.8
2	3.88	31260.1	319.9
3	4.02	32430.2	308.4
4	4.38	35341.7	283.0
5	4.46	35983.6	277.9

Table S6. Singlet excitations calculated for **2**^{d} in the ground state geometry.

State	Energy [eV]	Energy [cm^{-1}]	λ [nm]	f_{osc}
1	4.20	33873.6	295.2	1.010E-07
2	4.21	33930.9	294.7	3.917E-03
3	4.39	35382.5	282.6	1.000E-09
4	4.39	35407.7	282.4	1.405E-04

5	4.70	37885.1	264.0	1.046E-01
6	4.70	37891.7	263.9	4.175E-06
7	4.74	38203.2	261.8	7.395E-01
8	4.82	38868.1	257.3	1.249E-06
9	4.83	38987.4	256.5	1.397E-02
10	4.84	39045.3	256.1	9.330E-07
11	5.00	40329.8	248.0	1.345E-03
12	5.00	40350.9	247.8	1.210E-07
13	5.08	40989.1	244.0	4.205E-03
14	5.08	40993.0	243.9	1.357E-05
15	5.10	41122.9	243.2	1.107E-02
16	5.10	41135.0	243.1	9.074E-06
17	5.12	41332.9	241.9	1.259E-01
18	5.18	41794.9	239.3	4.140E-07
19	5.19	41888.8	238.7	1.462E-02
20	5.23	42146.8	237.3	1.718E-06

Table S7. Triplet excitations calculated for **2**[d] in the ground state geometry.

State	Energy [eV]	Energy [cm^{-1}]	λ [nm]
1	3.67	29579.2	338.1
2	3.67	29579.5	338.1
3	4.04	32561.0	307.1
4	4.04	32561.1	307.1
5	4.12	33191.6	301.3
6	4.12	33234.6	300.9
7	4.24	34165.2	292.7
8	4.37	35230.9	283.8
9	4.37	35275.1	283.5
10	4.43	35703.4	280.1
11	4.61	37205.1	268.8
12	4.61	37205.6	268.8
13	4.81	38784.0	257.8
14	4.81	38812.6	257.6
15	4.97	40094.1	249.4
16	4.98	40136.1	249.2
17	4.98	40182.8	248.9
18	4.98	40189.9	248.8
19	4.99	40267.2	248.3
20	5.00	40304.4	248.1

Table S8. Singlet excitations calculated for **2**[d] in the T_1 geometry.

State	Energy [eV]	Energy [cm^{-1}]	λ [nm]	f_{osc}
1	3.62	29219.6	342.2	2.344E-03
2	4.12	33261.2	300.7	5.837E-01

3	4.20	33844.2	295.5	3.249E-03
4	4.27	34444.2	290.3	9.767E-04
5	4.39	35384.3	282.6	4.191E-05

Table S9. Triplet excitations calculated for **2^d** in the T_1 geometry.

State	Energy [eV]	Energy [cm^{-1}]	λ [nm]
1	3.04	24486.1	408.4
2	3.55	28613.1	349.5
3	3.67	29578.8	338.1
4	4.04	32607.3	306.7
5	4.12	33209.9	301.1

Table S10. Singlet excitations calculated for **3** in the ground state geometry.

State	Energy [eV]	Energy [cm^{-1}]	λ [nm]	f_{osc}
1	3.76	30330.1	329.7	3.292E-02
2	3.80	30652.1	326.2	1.747E-02
3	3.89	31338.1	319.1	2.583E-03
4	3.92	31654.3	315.9	2.197E-02
5	3.97	32007.3	312.4	5.220E-03
6	4.04	32575.9	307.0	4.674E-04
7	4.05	32640.3	306.4	1.493E-04
8	4.10	33072.2	302.4	1.411E-03
9	4.39	35370.8	282.7	4.725E-03
10	4.43	35742.6	279.8	7.770E-03
11	4.50	36277.8	275.7	1.571E-02
12	4.52	36460.5	274.3	2.546E-02
13	4.56	36776.1	271.9	1.751E-01
14	4.58	36906.3	271.0	5.060E-03
15	4.59	37008.5	270.2	7.675E-03
16	4.60	37114.1	269.4	2.538E-02
17	4.62	37233.1	268.6	5.537E-02
18	4.65	37485.6	266.8	6.413E-02
19	4.65	37541.9	266.4	1.731E-01
20	4.66	37619.7	265.8	5.343E-02

Table S11. Triplet excitations calculated for **3** in the ground state geometry.

State	Energy [eV]	Energy [cm^{-1}]	λ [nm]
1	3.52	28380.9	352.3
2	3.65	29420.5	339.9
3	3.65	29436.3	339.7
4	3.67	29611.4	337.7
5	3.75	30231.5	330.8

6	3.78	30508.3	327.8
7	3.81	30763.9	325.1
8	3.88	31263.6	319.9
9	3.89	31408.7	318.4
10	3.97	32029.9	312.2
11	3.98	32074.9	311.8
12	4.02	32398.2	308.7
13	4.04	32544.5	307.3
14	4.04	32582.5	306.9
15	4.07	32822.1	304.7
16	4.10	33045.7	302.6
17	4.16	33519.3	298.3
18	4.21	33968.2	294.4
19	4.37	35214.7	284.0
20	4.41	35557.2	281.2

Table S12. Singlet excitations calculated for **3** in the T_1 geometry.

State	Energy [eV]	Energy [cm^{-1}]	λ [nm]	f_{osc}
1	2.94	23733.6	421.3	1.619E-01
2	3.62	29226.1	342.2	5.225E-03
3	3.66	29487.2	339.1	5.364E-02
4	3.70	29836.7	335.2	7.809E-02
5	3.74	30161.3	331.6	1.017E-02

Table S13. Triplet excitations calculated for **3** in the T_1 geometry.

State	Energy [eV]	Energy [cm^{-1}]	λ [nm]
1	2.48	19978.0	500.5
2	3.30	26589.7	376.1
3	3.50	28239.7	354.1
4	3.52	28430.1	351.7
5	3.63	29287.4	341.4

Table S14. Singlet excitations calculated for **4** in the ground state geometry.

State	Energy [eV]	Energy [cm^{-1}]	λ [nm]	f_{osc}
1	3.81	30769.6	325.0	3.993E-02
2	3.82	30817.8	324.5	1.227E-02
3	4.03	32495.5	307.7	4.725E-02
4	4.05	32658.0	306.2	2.004E-02
5	4.09	32949.1	303.5	6.349E-03
6	4.09	33021.4	302.8	8.594E-03
7	4.15	33452.5	298.9	9.135E-03

8	4.17	33594.1	297.7	1.196E-03
9	4.19	33809.1	295.8	8.996E-03
10	4.20	33871.6	295.2	1.116E-03
11	4.30	34662.2	288.5	2.536E-01
12	4.40	35498.5	281.7	1.057E-02
13	4.41	35580.8	281.1	7.425E-03
14	4.42	35662.0	280.4	6.561E-03
15	4.43	35742.7	279.8	1.462E-03
16	4.44	35829.3	279.1	2.218E-02
17	4.51	36367.6	275.0	1.116E-02
18	4.52	36453.1	274.3	1.533E-02
19	4.53	36572.5	273.4	6.971E-04
20	4.56	36782.5	271.9	2.012E-02

Table S15. Triplet excitations calculated for **4** in the ground state geometry.

State	Energy [eV]	Energy [cm^{-1}]	λ [nm]
1	3.53	28476.3	351.2
2	3.55	28664.3	348.9
3	3.64	29389.8	340.3
4	3.66	29502.9	338.9
5	3.80	30652.7	326.2
6	3.81	30746.4	325.2
7	3.88	31291.6	319.6
8	3.89	31334.9	319.1
9	3.98	32069.9	311.8
10	3.99	32170.7	310.8
11	4.00	32266.6	309.9
12	4.01	32349.3	309.1
13	4.11	33123.9	301.9
14	4.12	33193.9	301.3
15	4.16	33560.7	298.0
16	4.17	33660.3	297.1
17	4.19	33827.3	295.6
18	4.22	34035.6	293.8
19	4.31	34735.6	287.9
20	4.32	34821.1	287.2

Table S16. Singlet excitations calculated for **5** in the ground state geometry.

State	Energy [eV]	Energy [cm^{-1}]	λ [nm]	f_{osc}
1	3.81	30738.3	325.3	2.738E-05
2	3.86	31111.5	321.4	7.962E-04
3	3.99	32182.2	310.7	9.807E-04
4	3.99	32200.9	310.6	5.090E-02
5	4.10	33038.4	302.7	4.510E-03
6	4.10	33062.7	302.5	1.554E-03

7	4.12	33248.3	300.8	2.919E-02
8	4.13	33296.3	300.3	3.602E-03
9	4.14	33359.7	299.8	6.557E-03
10	4.15	33501.7	298.5	1.473E-04
11	4.17	33643.4	297.2	7.171E-03
12	4.23	34126.5	293.0	2.191E-04
13	4.27	34459.0	290.2	7.019E-04
14	4.31	34788.0	287.5	3.854E-04
15	4.32	34832.8	287.1	5.208E-02
16	4.32	34861.8	286.8	3.639E-04
17	4.33	34936.2	286.2	3.761E-04
18	4.37	35258.2	283.6	2.586E-02
19	4.39	35402.2	282.5	3.218E-04
20	4.40	35525.6	281.5	8.272E-03

Table S17. Triplet excitations calculated for **5** in the ground state geometry.

State	Energy [eV]	Energy [cm^{-1}]	λ [nm]
1	3.66	29510.1	338.9
2	3.68	29708.6	336.6
3	3.69	29752.7	336.1
4	3.69	29759.8	336.0
5	3.72	30017.2	333.1
6	3.75	30226.3	330.8
7	3.87	31183.4	320.7
8	3.89	31356.4	318.9
9	3.94	31776.8	314.7
10	3.94	31805.5	314.4
11	3.96	31928.0	313.2
12	4.00	32252.4	310.1
13	4.01	32351.3	309.1
14	4.04	32592.4	306.8
15	4.04	32601.5	306.7
16	4.08	32930.2	303.7
17	4.11	33113.2	302.0
18	4.11	33152.8	301.6
19	4.13	33315.1	300.2
20	4.13	33334.7	300.0

Table S18. Singlet excitations calculated for **6** in the ground state geometry.

State	Energy [eV]	Energy [cm^{-1}]	λ [nm]	f_{osc}
1	3.74	30203.8	331.1	3.98E-02
2	3.83	30929.0	323.3	6.83E-07
3	3.89	31351.8	319.0	7.67E-04
4	3.99	32214.2	310.4	2.57E-06
5	4.00	32286.3	309.7	3.21E-04

6	4.55	36708.8	272.4	1.84E-04
7	4.56	36811.5	271.7	2.52E-01
8	4.60	37115.5	269.4	7.09E-03
9	4.70	37930.6	263.6	1.93E-06
10	4.72	38066.6	262.7	1.94E-03
11	4.73	38116.8	262.4	2.58E-02
12	4.76	38375.5	260.6	4.17E-03
13	4.76	38409.8	260.4	2.81E-02
14	4.83	38933.1	256.9	2.76E-05
15	4.89	39457.1	253.4	3.66E-02
16	4.91	39615.6	252.4	1.10E+00
17	4.93	39728.9	251.7	2.00E-04
18	4.95	39917.4	250.5	1.35E-05
19	4.97	40082.6	249.5	5.50E-06
20	5.01	40412.0	247.5	2.76E-02

Table S19. Triplet excitations calculated for **6** in the ground state geometry.

**State**	**Energy [eV]**	**Energy [cm^{-1}]**	**λ [nm]**
1	3.56	28719.8	348.2
2	3.71	29920.5	334.2
3	3.71	29923.6	334.2
4	3.77	30417.4	328.8
5	3.82	30787.9	324.8
6	3.98	32126.3	311.3
7	3.99	32208.4	310.5
8	4.05	32667.9	306.1
9	4.05	32670.8	306.1
10	4.23	34081.1	293.4
11	4.54	36585.4	273.3
12	4.54	36629.4	273.0
13	4.59	36991.4	270.3
14	4.61	37200.1	268.8
15	4.61	37205.2	268.8
16	4.67	37696.6	265.3
17	4.71	38011.5	263.1
18	4.73	38112.8	262.4
19	4.73	38187.5	261.9
20	4.81	38777.5	257.9

Table S20. Total SOCME values between T_{1-5} and S_{0-5} states at the ground-state geometries.

Root		SOCME^{TOTAL} (cm^{-1})					
**T**	**S**	**2**	**2^{d}**	**3**	**4**	**5**	**6**
1	0	0.1	0.7	26.5	39.7	131.8	0.6
1	1	150.2	93.9	70.6	75.6	55.0	0.2

1	2	4.9	95.0	37.4	62.7	6.3	0.3
1	3	2.8	2.3	83.3	87.2	12.7	4.7
1	4	0.5	3.2	56.7	19.7	2.5	0.8
1	5	111.7	5.0	31.4	17.9	19.5	60.9
2	0	8.4	0.7	30.2	30.4	2.3	2.5
2	1	0.4	94.9	41.8	84.9	5.1	0.1
2	2	1.5	93.9	50.4	59.2	4.4	85.7
2	3	145.6	2.3	50.2	24.9	9.1	140.3
2	4	5.2	3.2	72.6	80.6	48.7	0.1
2	5	2.7	4.8	21.4	35.8	41.0	2.3
3	0	0.7	10.7	13.6	15.4	23.9	3.2
3	1	12.3	0.3	103.7	103.8	14.3	0.8
3	2	10.1	2.8	51.5	48.7	38.4	143.7
3	3	1.5	110.1	43.5	48.3	35.7	83.7
3	4	3.8	65.3	18.8	24.7	32.1	0.1
3	5	10.9	1.3	40.7	21.9	22.3	1.4
4	0	68.4	6.4	19.9	15.1	28.8	227.9
4	1	0.5	0.2	43.7	36.4	10.0	0.3
4	2	3.4	4.8	85.6	87.1	44.9	1.9
4	3	9.2	65.3	29.2	19.3	47.7	0.5
4	4	370.0	110.1	56.7	64.6	25.1	3.7
4	5	449.4	2.2	23.5	32.0	48.6	0.2
5	0	0.0	94.8	17.9	7.8	71.6	1.2
5	1	79.0	1.7	35.7	99.9	75.2	11.2
5	2	15.3	0.0	10.9	40.6	2.2	0.4
5	3	2.7	6.9	50.9	32.9	71.9	0.9
5	4	0.1	0.1	34.0	48.7	15.3	0.0
5	5	62.1	0.8	29.6	39.4	17.9	1.6

Table S21. Total SOCME values between $T_{1\text{-}5}$ and $S_{0\text{-}5}$ states at the optimized T_1 geometries.

Root		SOCME^{TOTAL} (cm^{-1})		
**T**	**S**	**2**	**2^{d}**	**3**
1	0	2.7	3.7	71.7
1	1	159.5	143.1	125.5
1	2	5.8	5.8	106.4
1	3	117.5	2.8	145.1
1	4	10.2	101.8	279.5
1	5	8.5	3.7	52.8

2	0	91.4	79.9	134.7
2	1	4.0	2.0	285.5
2	2	293.7	272.9	23.5
2	3	445.7	4.7	49.0
2	4	138.2	234.5	120.3
2	5	27.0	12.1	24.6
3	0	9.1	0.8	27.6
3	1	27.1	0.7	17.0
3	2	5.4	0.1	28.6
3	3	21.9	134.1	5.1
3	4	8.3	3.0	18.9
3	5	151.5	2.2	3.5
4	0	7.6	9.3	52.3
4	1	347.7	29.6	17.2
4	2	4.7	4.8	3.8
4	3	282.5	3.1	40.9
4	4	20.3	23.6	25.6
4	5	29.0	6.6	4.5
5	0	5.2	66.4	6.9
5	1	120.4	2.6	7.5
5	2	5.5	10.4	10.1
5	3	83.3	0.5	1.4
5	4	13.9	1.7	11.4
5	5	5.1	6.1	78.4

Table S22. XYZ coordinates of optimized ground state of compound **2**.

Cu	-0.38864437658911	2.71729468323341	11.91186701651776
Cl	-0.09478332505181	2.55245924480934	14.00791459637760
N	-0.63884340107563	2.87565014956587	10.03222630426289
N	-1.16799943965332	3.23020376954275	5.91083888250134
C	0.31605525786567	3.39083671667369	9.23872320580214
H	1.22771270274830	3.70735357742480	9.73127919843952
C	0.19021061674731	3.52809250657765	7.88079844189028
H	1.01356198588489	3.95372470690319	7.32569735334733
C	-0.99766828874793	3.11527396250885	7.23875989513285
C	-1.99478323783162	2.57457726532346	8.07985578800556
H	-2.93966827666460	2.22980401755455	7.68527627214375
C	-1.77309490819690	2.47974472701058	9.42930398682071
H	-2.53857695356861	2.06558142556081	10.07456309920365
C	-2.40030486669221	2.78938830882130	5.29504043724242
H	-2.57027118500160	1.72131017684999	5.46240312366441
H	-2.34069550392434	2.96213248347488	4.22350970798263

H	-3.26150115219212	3.34069891032851	5.68516920367564
C	-0.11956802251122	3.79583688111487	5.09054725994101
H	0.11232627235581	4.82226954549316	5.39117357316026
H	-0.45032970697424	3.80983492607573	4.05528080308120
H	0.79877180907331	3.20339801515249	5.15044185080672

Table S23. XYZ coordinates of optimized T_1 state of compound **2**.

Cu	-0.42550189414211	2.72060139997879	11.91365189416443
Cl	-0.14675034994221	2.48431429068858	14.00505965005618
N	-0.67631850657870	2.92667237894644	10.08041179060382
N	-1.23015863402335	3.29419204013480	5.91347062525596
C	0.40327635308354	3.19542959551098	9.21821996253787
H	1.36733381283296	3.30696076505610	9.69329242892488
C	0.25658826263776	3.29794208355616	7.87428975253216
H	1.13576813008699	3.48416641683255	7.26926809618439
C	-1.01672098614088	3.12449277396280	7.26876191684478
C	-2.13302399821323	2.90926122207863	8.16248913044516
H	-3.14858058325429	2.86453460690674	7.79175148642928
C	-1.91967397905765	2.83047396742235	9.49827848444517
H	-2.74837455347994	2.67975239839610	10.17881838924899
C	-2.16568944009343	2.46102890533181	5.20467410843574
H	-1.63706505541559	1.59923215864517	4.77366569759852
H	-2.62612459569519	3.02614170036899	4.39364887506430
H	-2.93130562240584	2.08360921563704	5.87787688888578
C	-0.27838235699501	4.00824903279975	5.10776964997290
H	0.09623337341436	4.87509396705801	5.64929918368103
H	-0.76282458039790	4.33021389363523	4.18592580538456
H	0.56920120377981	3.36310318705266	4.84004618330336

Table S24. XYZ coordinates of optimized ground state of dimeric compound $\mathbf{2}^{\mathbf{d}}$.

Cu	-0.39381104548279	2.83118802338084	11.92941197570092
Cl	-0.52393996502240	2.62281396848316	14.03030800439273
N	-0.54988041256935	2.77107169451275	10.01937265237046
N	-0.92302164727535	2.53531840543542	5.87072820492323
C	0.33748877583824	3.35408745281132	9.19683371118834
H	1.14832773756763	3.88698838682661	9.67910613006723
C	0.25797479165252	3.30492910185141	7.82839654681089
H	1.02055044260806	3.80437073227549	7.24826888621630
C	-0.80363016326461	2.61222264918141	7.20827921523104
C	-1.73262690230438	2.00455494633459	8.08015690089843
H	-2.58180879303354	1.45194946235710	7.70476154269336
C	-1.56462454132016	2.10959172074647	9.43709700645138
H	-2.27722645939548	1.64152731938797	10.10586517768928
C	-2.02552076292132	1.80953191135702	5.28053004912618
H	-2.01463243694082	0.75552901569548	5.57572818563832
H	-1.94323131251953	1.86164006433430	4.19782734063505
H	-2.98919463443820	2.23851889080518	5.57283246761894
C	0.05566365031136	3.17124732735993	5.01708534446611

H	0.09262150478437	4.25173763866485	5.18754271680105
H	-0.21605652083399	2.99822366658580	3.97884809325329
H	1.05741301504542	2.76268598394588	5.18346943983609
Cu	1.78393398415063	4.60541204638754	14.14438103062317
Cl	1.91406302635456	4.81378596174845	12.04348498928318
N	1.94000259350008	4.66566164789232	16.05449160425615
N	2.31330482238172	4.90217830777301	20.20305500817739
C	1.05363811547379	4.08131638065818	16.87715310366027
H	0.24353265640594	3.54719352214525	16.39499325025302
C	1.13330730677593	4.13057297818269	18.24558238266848
H	0.37160806959514	3.62991737399723	18.82581848927404
C	2.19389374757907	4.82498241092137	18.86552158344053
C	3.12188921986929	5.43397001598121	17.99349998417237
H	3.97040247454294	5.98768046046173	18.36876814207950
C	2.95387065960522	5.32863696192683	16.63658958625714
H	3.66572784662028	5.79766817542617	15.96770971592457
C	3.41474236875103	5.62970993873895	20.79306564180349
H	3.40316073407872	6.68319206959722	20.49611384722256
H	3.33146085497568	5.57939055122664	21.87577605940693
H	4.37908329247942	5.20110525867622	20.50233885233711
C	1.33649553623726	4.26359349673528	21.05686149100342
H	1.30179983023310	3.18312690696004	20.88576290326935
H	1.60843806418996	4.43657541012745	22.09504893075453
H	0.33376324323309	4.67006890587737	20.89129295108273

Table S25. XYZ coordinates of optimized T_1 state of dimeric compound **2**[d].

Cu	-0.39381108268281	2.83118794274092	11.92941191228215
Cl	-0.52393997907588	2.62281400672701	14.03030802963438
N	-0.53289988837444	2.71303047965862	10.06471583991264
N	-0.76187733513541	2.17508268512312	5.88760138045171
C	0.23841713340582	3.43796654046675	9.19502032046370
H	0.94547329896537	4.11650251944225	9.65591023776135
C	0.15353852317151	3.33767995473561	7.84503373307819
H	0.83440648710437	3.91721814418582	7.23573677530596
C	-0.78799232269488	2.40627729768730	7.24918446460652
C	-1.54723151314882	1.61708438533415	8.14903055615500
H	-2.26337188868681	0.89279678593203	7.77959488922707
C	-1.42884370026312	1.78450585596004	9.49023261413727
H	-2.02450998393944	1.21424490531735	10.18811675003351
C	-1.27017180887351	0.94469089746241	5.34681671799530
H	-0.97336987845558	0.10677651422376	5.97538071337802
H	-0.87390736665491	0.80824374039626	4.34057107139174
H	-2.36653016955430	0.96758795091026	5.28131560030585
C	-0.62462221108955	3.26338845552188	4.95540175416235
H	-0.11573668664172	4.10518993722716	5.41765344871887
H	-1.61937208246693	3.60242072070133	4.63374955443671
H	-0.07108587727595	2.93092787446091	4.07641787774382
Cu	1.78393403239744	4.60541203392349	14.14438108549196
Cl	1.91406302936125	4.81378601660856	12.04348497259151

N	1.94341680678076	4.67023056295348	16.05383663506019
N	2.36211483018917	4.88222778556853	20.19921166860783
C	1.14022306027253	3.98058887812966	16.88029509684335
H	0.38280386705408	3.37098370203425	16.40234750899176
C	1.23818366388525	4.01700375453382	18.24792550408726
H	0.54631314713172	3.42687338716012	18.83150259131731
C	2.22753417358106	4.81354524103736	18.86275629189979
C	3.06763112329811	5.53431618618318	17.98683677954652
H	3.85694785487661	6.17190887646494	18.35800778053571
C	2.88906968794044	5.43110106721304	16.63115553292565
H	3.53418049613427	5.98466614549672	15.95924025334988
C	3.39570930685115	5.70736298081558	20.78331326738422
H	3.27559876706649	6.75780010626056	20.50067894460748
H	3.33355874584400	5.63641126171752	21.86623628442284
H	4.39323010460257	5.37965495003533	20.47350227186955
C	1.48344800755204	4.11917189323136	21.05728538666198
H	1.57287630436559	3.04483128770108	20.86772976167225
H	1.75181208231447	4.30702379624664	22.09387425848437
H	0.43713650672201	4.40728453041519	20.91543564065224

Table S26. XYZ coordinates of optimized ground state of compound **3**.

Cu	5.87509685558224	7.38092940151197	10.81238582909400
Cu	5.42998879243928	9.01536126029000	8.54752857485216
Cl	4.50144708317854	9.46660910395886	10.82229475010031
N	7.67327251500941	8.06825391833833	10.48286192930384
N	4.48941576351722	7.38283092398976	8.03626246572496
N	11.12057005533628	10.06865260987141	9.25312605411973
N	4.65677258514213	5.90511650409554	11.18857316891236
N	6.68966465252485	10.45189511702272	8.16152073804302
N	2.59662165110000	3.67062153592074	7.87204320136336
N	1.93510289851816	2.78568104832798	11.71865553777094
N	9.60757568031277	13.37826115347959	7.58078861622426
C	10.03149549855818	9.40140889178497	9.67733301507152
C	8.07777503180386	9.20617243465751	11.06826723006432
H	7.42535451434103	9.60128311054347	11.83785130147942
C	4.55915671135003	5.03684754908082	7.54980601601505
H	5.16777956703264	4.20020980693570	7.23825149270696
C	3.18229476011920	7.24315187795152	8.30694145080902
H	2.66493466761332	8.14004123607484	8.62630147166546
C	7.62854493828809	10.33512524995935	7.20915117900612
H	7.59158269122660	9.43393360877982	6.60871079547922
C	8.47108700654508	7.57418125905659	9.52346624731908
H	8.15176932130157	6.64391690619119	9.06801394917282
C	3.19742470094441	4.87495543325070	7.88126484505860
C	5.13960951412932	6.27675925745555	7.64217149553999
H	6.18953758575697	6.39892040305641	7.40210857866387
C	9.62428816064987	8.18207299925030	9.09538133697837
H	10.19453606043750	7.71635306846401	8.30447853817836
C	9.22024527173719	9.88170876691194	10.72614505928616

H	9.46097308655543	10.79323503387151	11.25346442684756
C	11.93017422475438	9.53022164102275	8.18427047548160
H	12.37095298008304	8.56562792872530	8.45696074441561
H	12.73682090265651	10.22624191659121	7.96771709490711
H	11.34017825704660	9.39378032335704	7.27263760042135
C	6.72663884393232	11.56859255951889	8.90356130442306
H	5.97134680211336	11.64302071156767	9.67790473774876
C	2.50634292178005	6.05331174588061	8.23203272744759
H	1.45419168534609	6.03840660406740	8.47631399054421
C	4.99534554328673	4.62526776176741	10.96556460496688
H	6.01128306972324	4.45031279094305	10.63185225518150
C	3.40204970565603	6.12628860779590	11.60753863452641
H	3.13892596919903	7.16526187184503	11.77176644619275
C	7.65392796114342	12.56725221383001	8.74167616545169
H	7.60596396743779	13.42999686843725	9.39048104114106
C	8.60359844086316	11.27079533301669	6.97238596866978
H	9.32127026287970	11.08509902344895	6.18637035691671
C	11.51558415308882	11.29987923464086	9.89892926587732
H	10.69428184471711	12.02105268541985	9.90538603741493
H	12.34541781244473	11.73722688461150	9.34913889971955
H	11.83426007275565	11.13186363633110	10.93337722178567
C	2.47456192007308	5.13548466376079	11.81188494052383
H	1.48601936774236	5.41142850105327	12.14994684587585
C	8.65588412858604	12.44126438970200	7.75714197843774
C	3.34706597433912	2.49060117885856	7.50958342491648
H	3.70750356319905	2.54161666347782	6.47686579069504
H	2.70442179853137	1.61886863515855	7.60448819151114
H	4.20959346508384	2.35499174611750	8.16949342210404
C	2.81783137167550	3.79071223087868	11.56153473288998
C	4.14207602991808	3.56397218612997	11.13176460874087
H	4.50528161122680	2.56785569767521	10.92349531742621
C	1.19621213173042	3.55619808103015	8.20941882744404
H	0.99267306410053	3.98775356151862	9.19316329297579
H	0.92278726636186	2.50423569928875	8.23706859833919
H	0.55977964767478	4.06038061389928	7.47442585396975
C	2.33217996926687	1.42272444337514	11.44797945972548
H	2.66915922813056	1.30455330978060	10.41367091781115
H	1.47850147327105	0.76801900419721	11.60407006610862
H	3.14053234931418	1.09829205689604	12.11156018687374
C	9.60175027636055	14.57782157353218	8.38717099939471
H	8.67708039586875	15.14769150572023	8.25133222574635
H	10.43766990133281	15.20676959889003	8.09151950769250
H	9.70772339114604	14.34488498287159	9.45174889994357
C	0.60006681480457	3.06144668301808	12.19949232421963
H	0.61810759552452	3.51644431041955	13.19505677658286
H	0.04600957430616	2.12806696470878	12.25957804611876
H	0.06273179374876	3.73567700689717	11.52482998805864
C	10.62050095510960	13.20099484820681	6.56539511572235
H	11.18515111360610	12.27728491573798	6.72459638551055
H	11.31570287214730	14.03576941270148	6.60932406892805

H	10.18459691385894	13.16813172159478	5.56141833762657

Table S27. XYZ coordinates of optimized T_1 state of compound **3**.

Cu	7.22749890293775	6.78016743672932	11.04593113444457
Cu	5.01932122474194	9.03097235734731	7.64760223813458
Cl	6.17273617264987	8.49286474696904	9.99643250741369
N	8.92118636313127	7.62293450407491	11.51377664053110
N	3.62326291821112	7.70829615354342	7.63971970707616
N	12.56470056140965	9.30702111150325	12.59938250789913
N	6.01484060859958	5.36673881341915	11.01796670396099
N	6.13239654011301	10.49484880293686	7.08283548732189
N	0.58874093698120	4.84875786177726	7.47469750218031
N	2.99326226111623	2.39411329256784	10.67768190881099
N	8.46810924625377	13.67548954081724	5.72467902649426
C	11.39108871579750	8.76109972682147	12.24849171106268
C	9.42520044649165	8.67955800729526	10.85908735695420
H	8.82098787801341	9.06482004583118	10.04697084355304
C	1.69880439945827	6.78092280169179	6.55004846279616
H	1.01672943084825	6.85249336579475	5.71516294821335
C	3.49154725430685	6.76139906444278	8.58450853083580
H	4.22409835055449	6.77973576090158	9.38163553941750
C	5.99114103754834	11.00028607568223	5.84439199212980
H	5.23759867369816	10.53498045468111	5.22006515243953
C	9.64400142228322	7.12997026825266	12.53272027433865
H	9.22310836907063	6.27494759713013	13.05068574759679
C	1.56051648223506	5.77657927699992	7.53345267542593
C	2.71734752256814	7.69351018764914	6.64577091509505
H	2.82259580509090	8.46201466091708	5.88902164129407
C	10.84802151463800	7.64770132717543	12.92965008473447
H	11.36088003848596	7.18971553764979	13.76276663592972
C	10.62136809774392	9.26558582307772	11.17568101663557
H	10.95296955684541	10.11305241599791	10.59360240729616
C	13.31695587388682	8.76447955007828	13.71057071814023
H	12.74533701588155	8.81810174910205	14.64196085551290
H	14.22877161133081	9.34269175339742	13.83484045735287
H	13.59238519236025	7.72060154675692	13.53286143415165
C	7.06443233516869	11.07066375346656	7.86025857679813
H	7.17158689216252	10.65608022886092	8.85519146681444
C	2.50628663473781	5.80836476477694	8.57989334540544
H	2.49097280642472	5.08681452973570	9.38415819045711
C	5.98392592810686	4.44506851265976	9.98639312312555
H	6.77731037983709	4.53175748702241	9.25192451436175
C	4.87624977983103	5.38464174024681	11.84436115492540
H	4.81299121179135	6.20185957359173	12.55004155892659
C	7.85533673385745	12.11715807142979	7.46108855840977
H	8.57692130501299	12.51492789757721	8.16015008620264
C	6.73255263253610	12.04273770732737	5.35137680403940
H	6.54598840230853	12.37831512553155	4.34150765079971
C	13.08157768663915	10.44759118699373	11.87368623632676

H	13.23944033065072	10.20894946617261	10.81768877296130
H	14.03593379051288	10.73725766178078	12.30539717441678
H	12.40099210278529	11.30204329384906	11.93749164330415
C	3.91840266750668	4.41106542231687	11.78808467762089
H	3.09886498070898	4.46992393634252	12.49389949995348
C	7.71522501328512	12.65209239202619	6.16203166899780
C	-0.40084008822622	4.90014207859966	6.42179885208418
H	-1.00297664503348	5.81391012341189	6.47396548938411
H	-1.06479837990126	4.04491138303199	6.52030961359935
H	0.06992174181005	4.85382515313997	5.43600710227775
C	3.97887519688567	3.37480352377697	10.83760270969427
C	5.05156393317494	3.47397336983622	9.87839072097822
H	5.10232537354370	2.79785308749907	9.03405092514937
C	0.42883178815849	3.89625000766207	8.55164461213188
H	1.35597907601563	3.34886996508272	8.74076227404907
H	-0.34074088361541	3.17915557224438	8.27618492508528
H	0.12755398029572	4.38800518717588	9.48333460300303
C	3.45695816248384	1.03224885634428	10.50156828855940
H	4.26771987829072	0.99273915022512	9.77601123896405
H	2.63642705017270	0.41357237812302	10.13276395927416
H	3.82249601407640	0.59919504138820	11.44654945830106
C	9.45824439774875	14.27174009850425	6.59422780835311
H	8.99814110385955	14.69872713136961	7.49097941237800
H	9.96515719000411	15.06923602471414	6.05710996593059
H	10.20736309701829	13.53794865294273	6.90716780552441
C	1.84876035970039	2.47623858058497	11.54768514303835
H	2.10013331134449	2.27598608202521	12.60221468451315
H	1.10979556498620	1.73858561043330	11.23096093442581
H	1.39424139744408	3.46618747481838	11.48786460031135
C	8.28174761752125	14.19603485976207	4.38786594773711
H	8.46936806687524	13.42816958510483	3.63107094035121
H	8.98056005164517	15.01281636848484	4.22656086851950
H	7.26632660457133	14.57903028299050	4.24610364735765

Table S28. XYZ coordinates of optimized ground state of compound **4**.

Br	8.66499525945964	8.06647702372016	6.29008852966503
Cu	7.09713607481091	9.46750113622237	7.84683471823789
Cu	6.20681563723510	7.16116085462895	6.53599556371094
N	5.13992975450228	8.54823359682215	5.66656640912043
N	6.31160345125234	5.44095305866486	7.45226574007996
N	6.73382165799312	8.27762538489143	9.35730653194992
N	6.86586781897001	11.22834027370584	7.02774387710050
N	3.42020389355465	12.07182264232196	4.24702302560168
N	6.13990621576851	14.84893362031213	5.08175812218894
N	5.85423388149870	5.24849546054563	12.08702974228754
N	6.58726516841551	1.99016179426514	9.78224796018065
C	3.44727259119732	10.23829815080912	5.81510407229557
H	2.59372012352779	10.61162983313978	6.36226117914336
C	5.49924570725173	8.11637849247820	9.85865640443732

H	4.74186086681274	8.80140909962598	9.49530679681580
C	4.06385275017943	9.09422277098737	6.25449668568763
H	3.68982161834714	8.58508842504455	7.13527578654558
C	7.67983978415888	7.44618664055828	9.82099471263423
H	8.67067976575897	7.57544045246960	9.40150278765549
C	6.38348713018084	13.68781926991998	5.71961323487159
C	3.94582921556753	10.91080831184939	4.67914979620120
C	7.41731379307283	12.81582868150560	5.31954968367148
H	8.06388889482120	13.05151676458372	4.48646230523196
C	5.03498160550782	10.29391579208333	4.02939101394639
H	5.46976148196757	10.71418714251682	3.13421494087152
C	7.60558157135532	11.62968098240222	5.98364323180257
H	8.38188898397736	10.94263057466143	5.66543300423095
C	5.58821492267965	9.15668754562500	4.55742378663608
H	6.45734072671955	8.70961027921949	4.08960559813328
C	6.14400852856648	6.24807513600838	11.23387968240596
C	5.61741317001071	13.26591002669229	6.82622610040587
H	4.80853580030728	13.86705075849800	7.21583075125715
C	5.23917382649826	4.91118664494756	8.06156884989846
H	4.30216693440171	5.43781009647002	7.92471616407577
C	5.89164611498638	12.06239801142296	7.42504334919826
H	5.29947326751816	11.73733867497955	8.27234482198930
C	7.47208309470711	4.78726396714544	7.61052991923702
H	8.33217463105331	5.23007495097356	7.12082869222079
C	2.29626158397015	12.66022998115590	4.93814440257723
H	1.40763535367677	12.02226445570169	4.88529227245294
H	2.05963840879515	13.61620148816220	4.47767586640272
H	2.53162638752275	12.83678800412140	5.99197948255385
C	5.16047901077995	7.14633578658370	10.76783270167463
H	4.13552955890128	7.08552972405460	11.10441130917394
C	7.44878335826291	6.45700565574897	10.74130300562084
H	8.27396190531106	5.83042927860656	11.04695788682435
C	6.97686976031452	15.25910797875227	3.97697887199249
H	8.02099495480964	15.37505449354907	4.28489875904063
H	6.62282682414891	16.21588602218679	3.60130334787012
H	6.93973344502262	14.53507592456863	3.15672245926007
C	7.61235088161480	3.64064418202897	8.35084588943841
H	8.58966668220178	3.18519520801730	8.42147670290595
C	6.49462740703936	3.09293608174519	9.01432164181668
C	4.49449662247410	5.04716006210253	12.53249766926584
H	4.13296758192955	5.89147803332335	13.12887556133449
H	4.45267604571282	4.15109791790720	13.14682912046949
H	3.81853987735696	4.91138997478684	11.68271258760340
C	5.27437153293552	3.77458152468836	8.82917720531633
H	4.35932653819264	3.42819525501160	9.28751029300965
C	6.90336519331164	4.37894790292807	12.56999690236001
H	7.39750941590005	3.85486213646778	11.74668002069608
H	6.46845738039629	3.63590687671381	13.23380011131795
H	7.66201834018670	4.93710656153078	13.12779429434310
C	5.06950079421811	15.71136804095990	5.52794082885402

H	4.10653292384242	15.19257699277830	5.50651680919244
H	5.00696033899512	16.57015345917767	4.86427107883473
H	5.24108062817325	16.07466665595915	6.54664935095799
C	3.94372000019066	12.70732923663184	3.05927167931326
H	5.02004459078985	12.87754681802871	3.14673079257549
H	3.45811344670561	13.67143088550282	2.92875600818221
H	3.76298998838871	12.10399744248765	2.16319599540520
C	5.40970288703844	1.45382155039403	10.42636218306084
H	4.95550413546009	2.18631914217845	11.10082581537274
H	5.69297208662473	0.58276960823969	11.01186980191884
H	4.65499228101127	1.14742954763483	9.69469079607509
C	7.85523032778039	1.31107976195112	9.92626623740169
H	8.22506194950829	0.93921660218928	8.96521509580496
H	7.72788399179763	0.46407522007431	10.59566177985389
H	8.61486986411219	1.97336120264826	10.35341581024948

Table S29. XYZ coordinates of optimized ground state of compound **5**.

Br	-4.97282460355213	11.39594382218259	15.21122719555300
Br	-2.30872639936517	8.70970993611804	16.84292681822460
Cu	-2.43861956055323	10.90389788424747	15.56728181560440
Cu	-4.33597083331402	9.98106406709799	17.12840999604673
N	-1.61779004556576	10.87869015882475	13.71790898230851
N	-5.68628797251304	9.70280023485156	18.55562721651307
N	0.41938131419020	11.60831371936623	10.15396479909060
N	-8.50452533069318	9.14477530481891	21.58616253470962
C	-2.22062448682674	11.45214024893789	12.66940749031756
H	-3.25388795089036	11.74643479995157	12.82085557276349
C	-1.60580371025840	11.68810822501707	11.46442528494856
H	-2.16988684642747	12.16600125549608	10.67639418495373
C	-0.25202882450332	11.33605623820234	11.29157624679811
C	0.36039776186462	10.68683836765226	12.38323670942676
H	1.38619879254003	10.35076022486644	12.33744878359450
C	-0.34720094798872	10.49524350937220	13.54375680855866
H	0.12755262818550	10.01738905398531	14.39427447975902
C	-0.26061149291668	12.22958986631878	9.04219015283610
H	-1.06454898158589	11.59575026623608	8.65228743292233
H	0.45557422320946	12.40571365332979	8.24301211827132
H	-0.69284382103400	13.19217400586006	9.33185459808085
C	1.80211883567631	11.21712457833608	10.01949865167479
H	2.40348907627795	11.61986390016892	10.83917356915200
H	2.19453617153869	11.61240620588574	9.08545993735230
H	1.92048906813500	10.12769954422279	10.01154244581755
C	-6.95761017382180	10.09953950128683	18.41715737763322
H	-7.20381807534290	10.58682653273376	17.47986848669752
C	-7.92721228712986	9.92417477609171	19.37441970060789
H	-8.92887333751299	10.27250773498489	19.16733671827409
C	-7.59713086468630	9.31266274672085	20.60041155110460
C	-6.25954267864154	8.88607923098463	20.73375843950793
H	-5.91285695985780	8.38506054338414	21.62604969667487

C	-5.36990031426694	9.10203105694517	19.71145245825200
H	-4.34082702437166	8.77524005195125	19.81235629816390
C	-9.89693827074054	9.44997129242447	21.34690070765143
H	-10.02819763971046	10.49357564934565	21.04988506817088
H	-10.45547046901467	9.29037661901890	22.26621475600413
H	-10.32598028284943	8.81415839714750	20.56393182140321
C	-8.14303603487089	8.42577503108950	22.78669208390161
H	-7.92430678545002	7.37060658656573	22.58456709843488
H	-8.97041862029988	8.47745069253144	23.49072648082476
H	-7.26691695214455	8.87284137418695	23.26210703896805
Br	-1.08086151092215	12.50650172210351	17.04180484272487
Br	-3.72727340498557	15.19017139867068	15.35927816342873
Cu	-3.61625113778283	13.02251600555643	16.66842876672792
Cu	-1.69849226023705	13.90477186736636	15.11356418734286
N	-4.44008066560996	13.04475649800295	18.51577588837832
N	-0.34894441569245	14.19808556238400	13.68719799845365
N	-6.48003623686643	12.30754986592268	22.07693725896557
N	2.45473863547829	14.76923460431712	10.64566900857840
C	-3.83747201966216	12.47160862992622	19.56458498850448
H	-2.80333815801669	12.17989117448963	19.41430332654065
C	-4.45332823514392	12.23311845702336	20.76853630715108
H	-3.88906510908613	11.75590006869382	21.55685607474263
C	-5.80815659877177	12.58159119588362	20.94000312756599
C	-6.42097505320681	13.22933613023729	19.84769154011639
H	-7.44781951198955	13.56238483426491	19.89215824850288
C	-5.71215465057576	13.42404059657790	18.68839865445135
H	-6.18721019839441	13.90110165859103	17.83762074193620
C	-5.79852984514478	11.69159055471614	23.19073543241725
H	-4.99721524647971	12.32938649827958	23.57959790812777
H	-6.51463658476520	11.51472603050485	23.98982974265071
H	-5.36261078050645	10.72981960556515	22.90394837794971
C	-7.86378067150136	12.69542103720262	22.21022271407357
H	-8.46288079145053	12.29312018049247	21.38873439958139
H	-8.25678221288706	12.29703590521920	23.14271262355786
H	-7.98476049147311	13.78458228193825	22.22044434232929
C	0.92308878625502	13.80109538572888	13.81807190292976
H	1.17389076101586	13.30948142285078	14.75186214914245
C	1.88831503964363	13.98118372730593	12.85729591502295
H	2.89100891702883	13.63217757401920	13.05812172302834
C	1.55237474942783	14.59796277548023	11.63550693241735
C	0.21441588502996	15.02586578801139	11.51071470986487
H	-0.13632818909250	15.53122234950209	10.62245486587284
C	-0.67054279133447	14.80484731111814	12.53603418955180
H	-1.70008748132188	15.13221784544493	12.44163358853164
C	3.84816833378239	14.46207755079627	10.87628492867362
H	3.97951200611001	13.41840733511539	11.17285392118987
H	4.40116216006839	14.62035835235349	9.95337899731299
H	4.28322044964209	15.09749144885508	11.65631703940424
C	2.08817323228628	15.49502048523100	9.45079183302371
H	1.87206019438710	16.54943699764234	9.65952413608490

H	2.91192282911379	15.44568216216459	8.74232947736854
H	1.20906641071441	15.05167839470793	8.97740840322457

Table S30. XYZ coordinates of optimized ground state of compound **6**.

Br	-5.93642328214798	10.31287261221263	14.44214109666866
Br	-2.66158059601967	10.43379158771242	17.04770312177362
Cu	-3.52793266107775	10.57378461474010	14.74898726492224
Cu	-5.07981597692262	10.16615927263771	16.72730588802689
N	-2.26487866092659	10.90640054827377	13.26394217624833
N	-6.24619289606520	9.80823101734946	18.28482333258368
N	0.42364321273033	11.61805545577388	10.15375131696182
N	-8.67598069112060	8.98328280480917	21.57444385962088
C	-2.67975860807517	11.09483572630785	12.00264108861056
H	-3.75160148481545	11.05024617269047	11.84208274582975
C	-1.83825074155751	11.33363692069459	10.94479894634343
H	-2.26552242096941	11.47609078205143	9.96252266594075
C	-0.44390320757169	11.38882700231908	11.15723318802054
C	-0.01552753032608	11.18919037727082	12.48706515382861
H	1.03163945140176	11.21360298289938	12.75256015185907
C	-0.94140852942067	10.95947435675228	13.47380371260165
H	-0.61638764467182	10.80842026616502	14.49776952561011
C	-0.06133454207350	11.82341796921568	8.80756934570509
H	-0.62240356453769	10.95517163952397	8.44815073163479
H	0.78757911294046	11.97800014638409	8.14640003079200
H	-0.71117727619870	12.70229782619107	8.74441483627678
C	1.84352108614842	11.67400334650091	10.42036370359261
H	2.08706338503575	12.47317722615163	11.12769234018606
H	2.37033085644237	11.86946887640680	9.48973546551869
H	2.21117457519480	10.72832485035709	10.83123425486458
C	-7.57783792505934	9.70092237610067	18.16842256122288
H	-7.98110305987922	9.83785195398088	17.17078655275099
C	-8.42140829416896	9.43431029153332	19.21784216649234
H	-9.48296930440141	9.36753032610579	19.02758233508061
C	-7.89216833669484	9.25257135495176	20.51363328996070
C	-6.49040476167312	9.36987736904628	20.62820420063268
H	-5.98970928796170	9.25024725938989	21.57827888660193
C	-5.73634017982626	9.64135035315380	19.51394060933725
H	-4.65852050315395	9.73239765325016	19.59752524044120
C	-10.10792961929576	8.87326221606103	21.40996608563652
H	-10.54287441726502	9.80836251983634	21.04250167691640
H	-10.55710060636722	8.64329955157866	22.37285806130106
H	-10.36967978377183	8.07421487648288	20.70923635205522
C	-8.08901126650532	8.80663610197468	22.88373906356960
H	-7.38049492675629	7.97237236763517	22.89506866437439
H	-8.87921589511005	8.59329739234956	23.59924841589552
H	-7.56239919750518	9.70842465517702	23.21198089370841

NMR appendix:

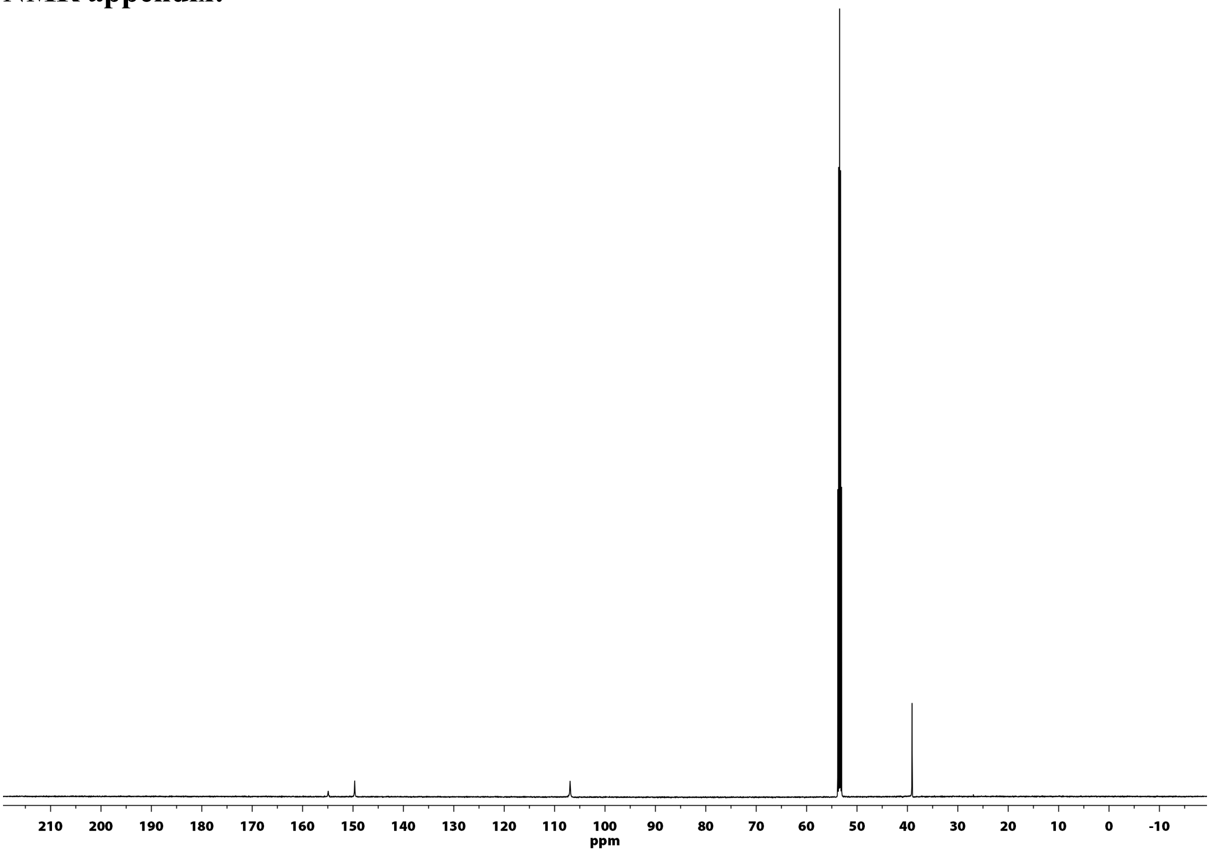

$^{13}C\{^1H\}$ NMR spectrum of **1** (150 MHz, CD_2Cl_2, 298 K).

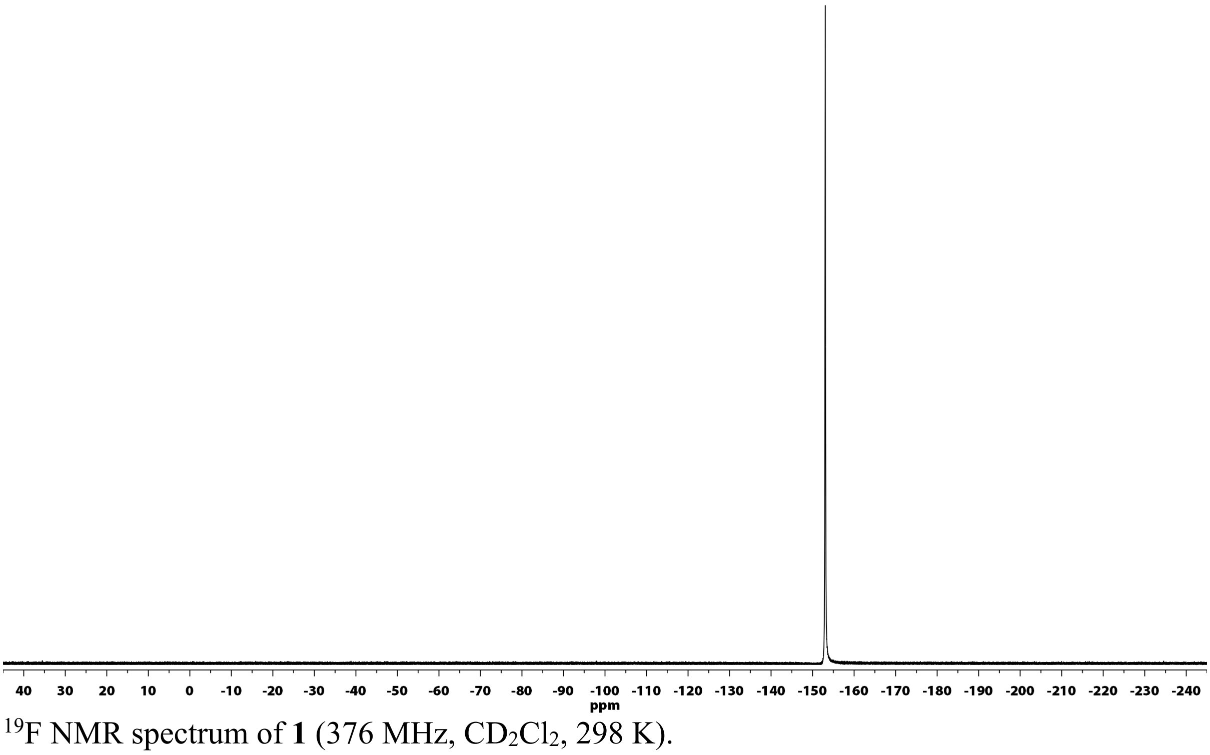

^{19}F NMR spectrum of **1** (376 MHz, CD_2Cl_2, 298 K).

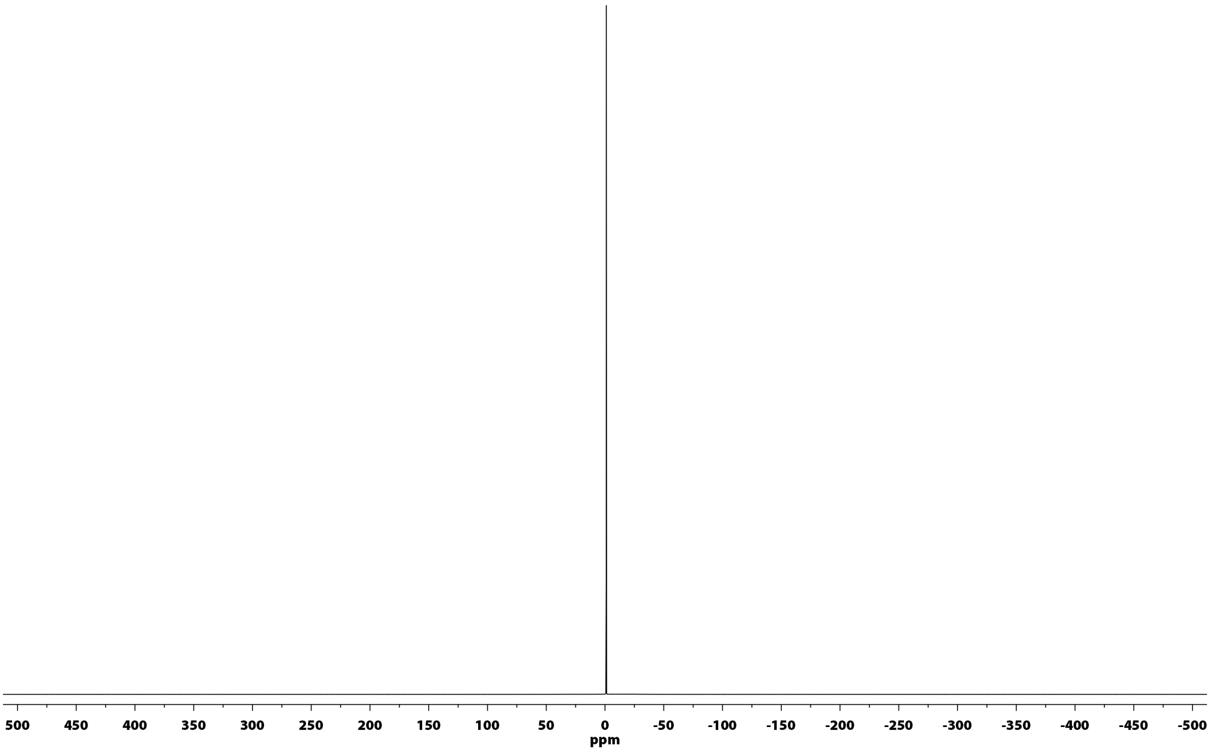

$^{11}B\{^1H\}$ NMR spectrum of **1** (128 MHz, CD_2Cl_2, 298 K).

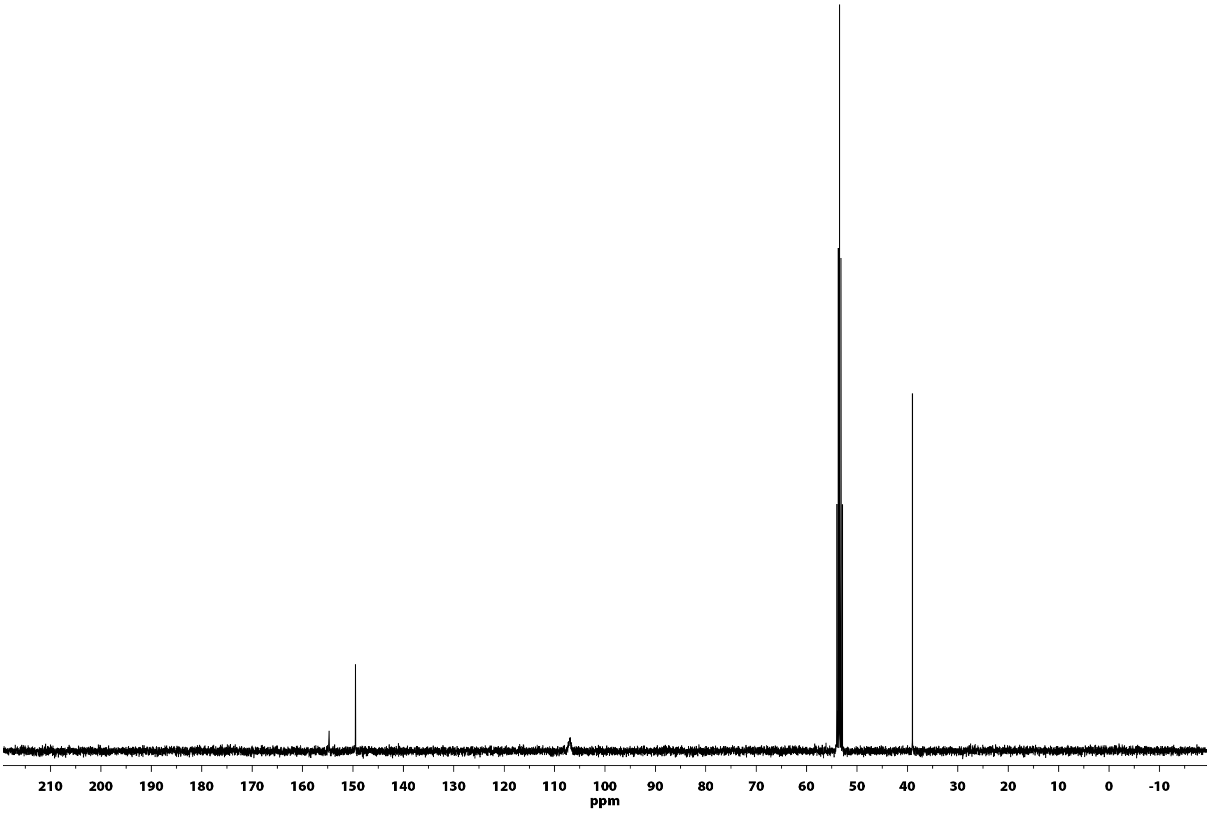

$^{13}C\{^1H\}$ NMR spectrum of **2** (150 MHz, CD_2Cl_2, 298 K).

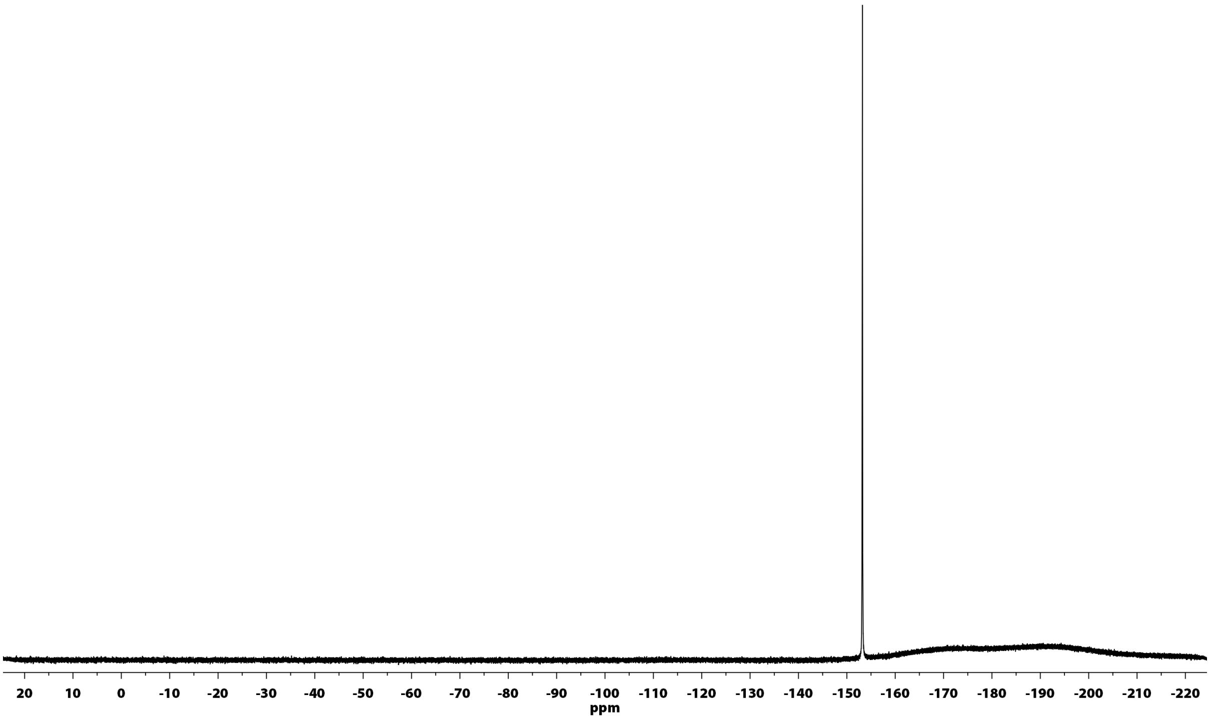

^{19}F NMR spectrum of **2** (376 MHz, CD_2Cl_2, 298 K).

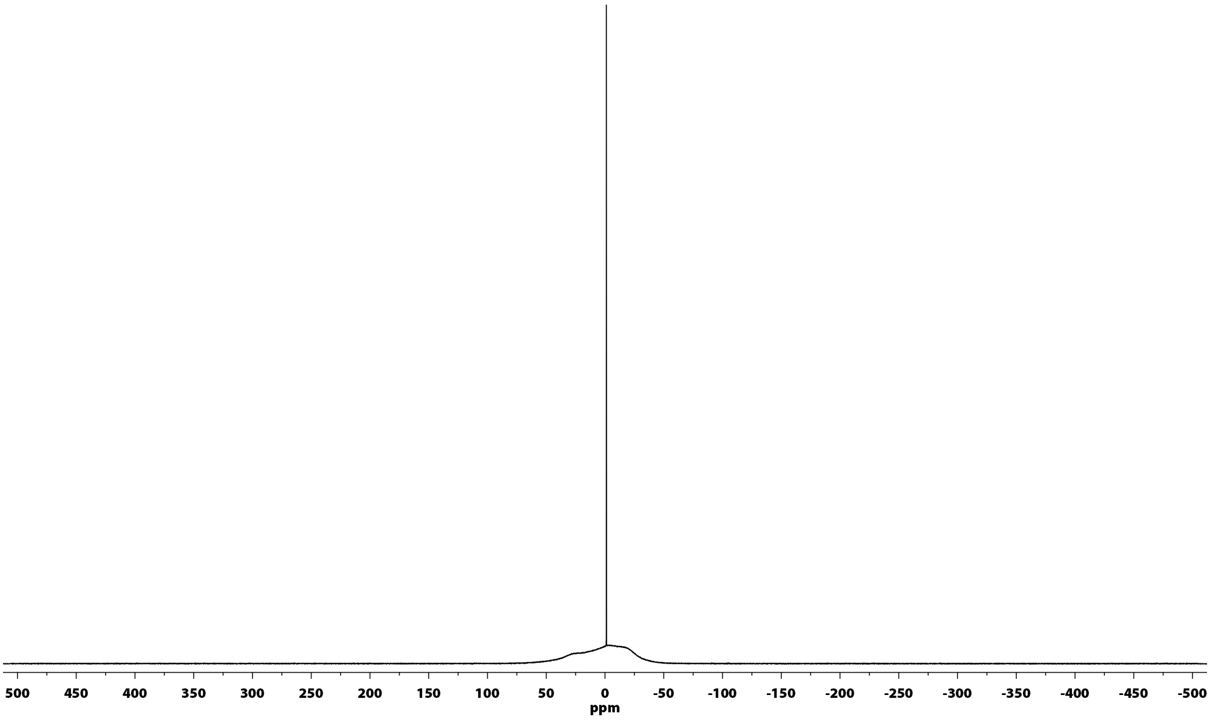

$^{11}B\{^1H\}$ NMR spectrum of **2** (128 MHz, CD_2Cl_2, 298 K).

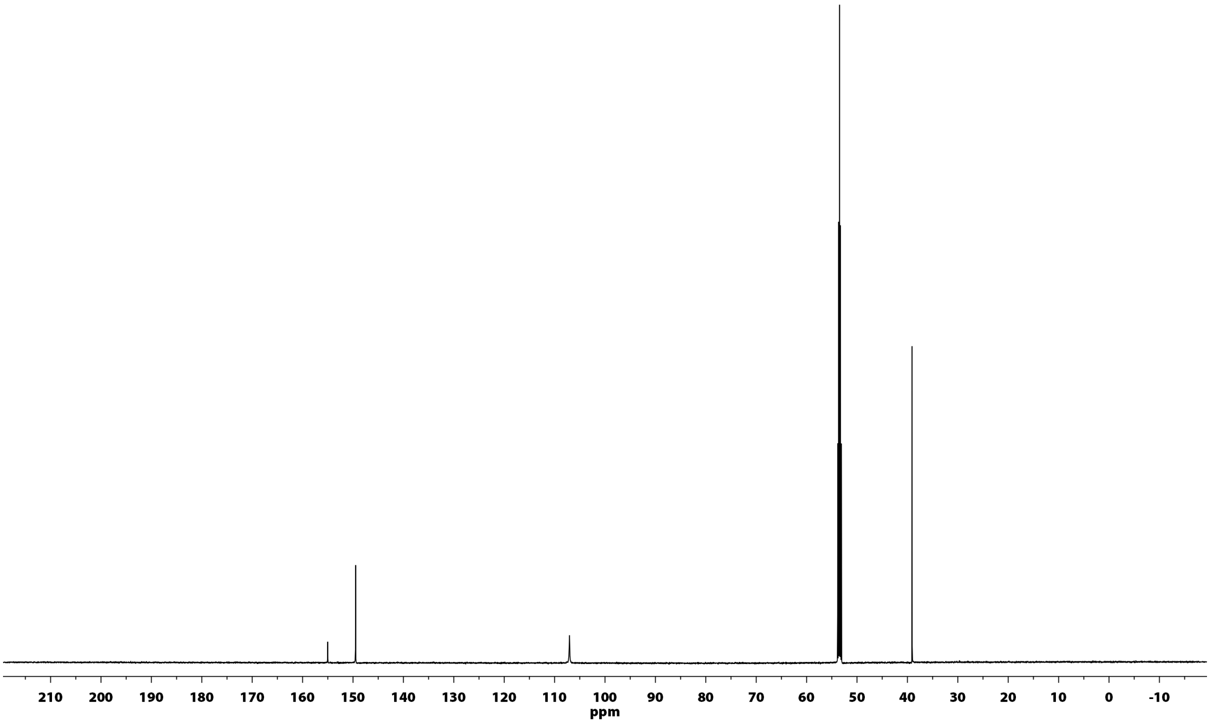

$^{13}C\{^{1}H\}$ NMR spectrum of **3** (150 MHz, CD_2Cl_2, 298 K).

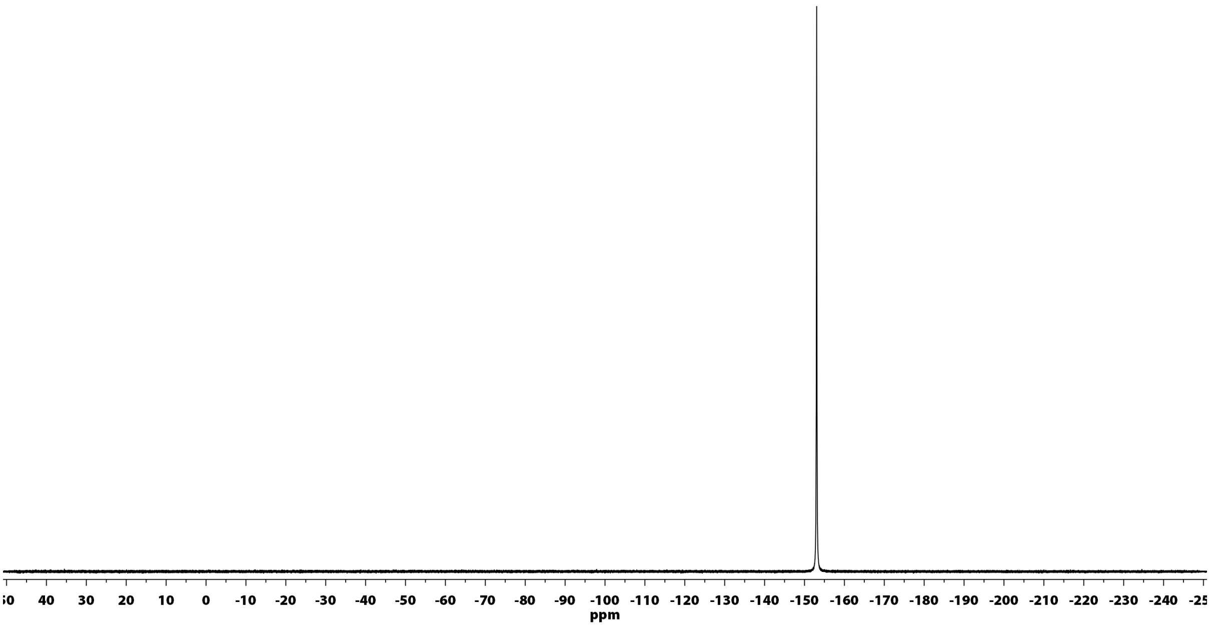

^{19}F NMR spectrum of **3** (376 MHz, CD_2Cl_2, 298 K).

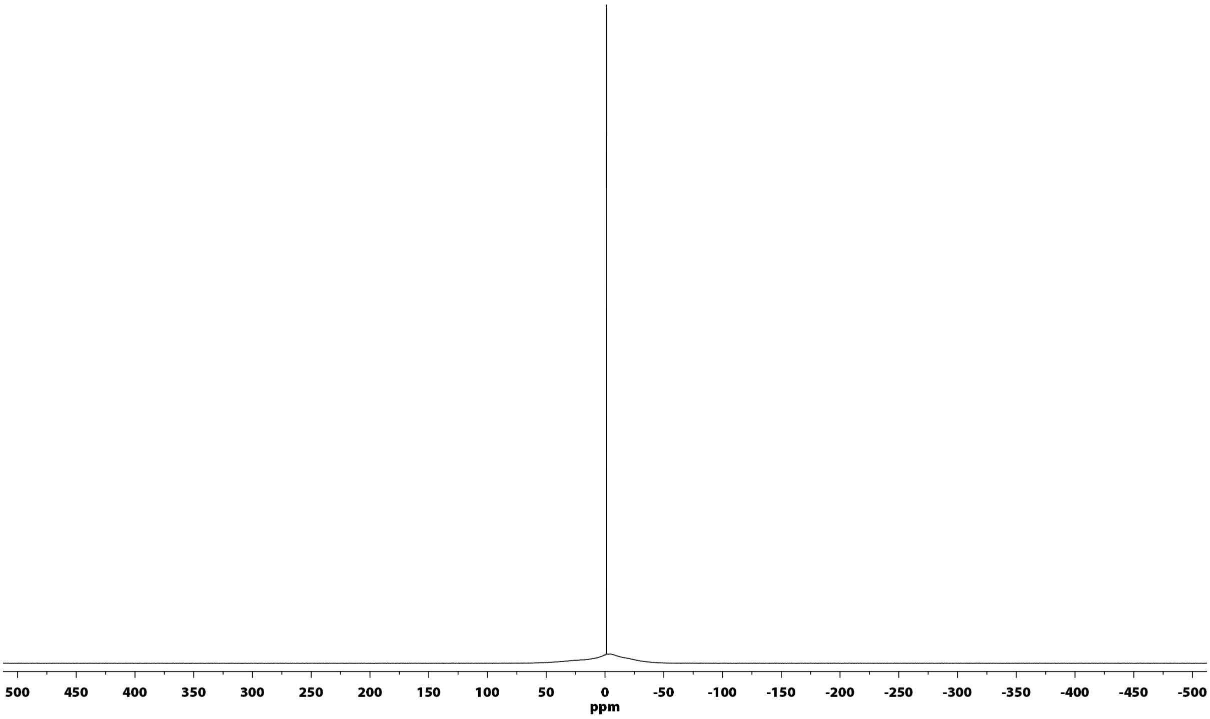

$^{11}B\{^{1}H\}$ NMR spectrum of **3** (128 MHz, CD_2Cl_2, 298 K).

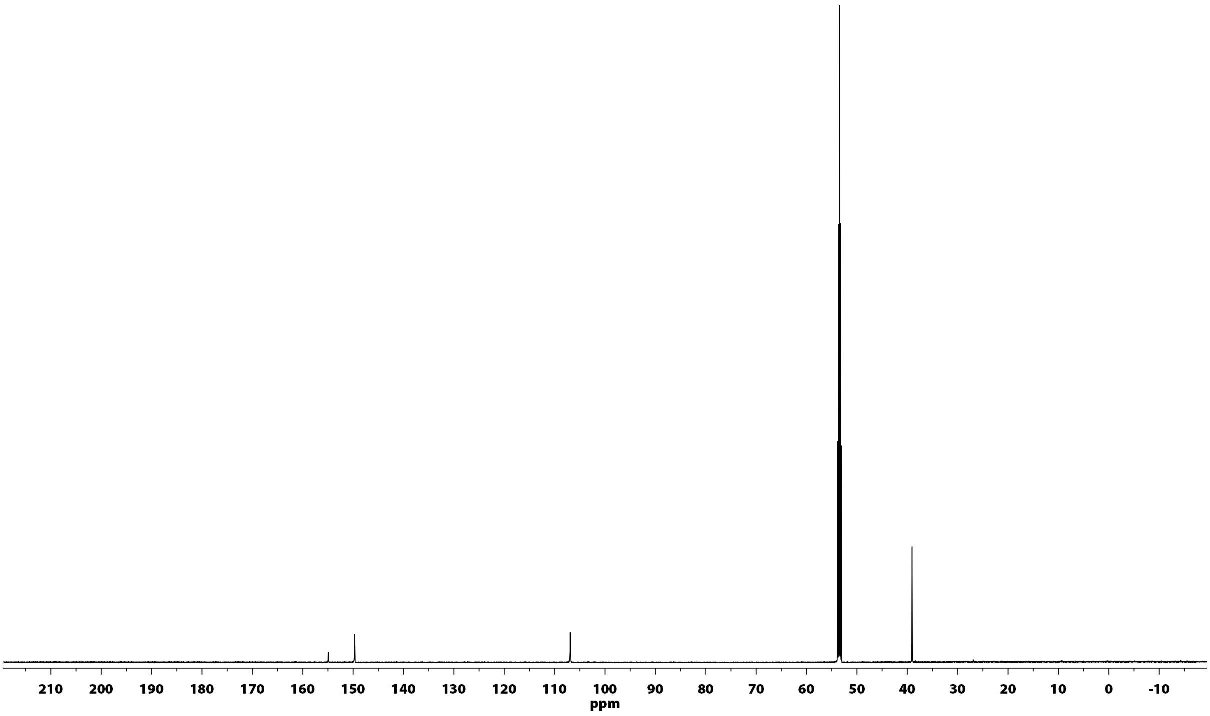

$^{13}C\{^{1}H\}$ NMR spectrum of **4** (150 MHz, CD_2Cl_2, 298 K).

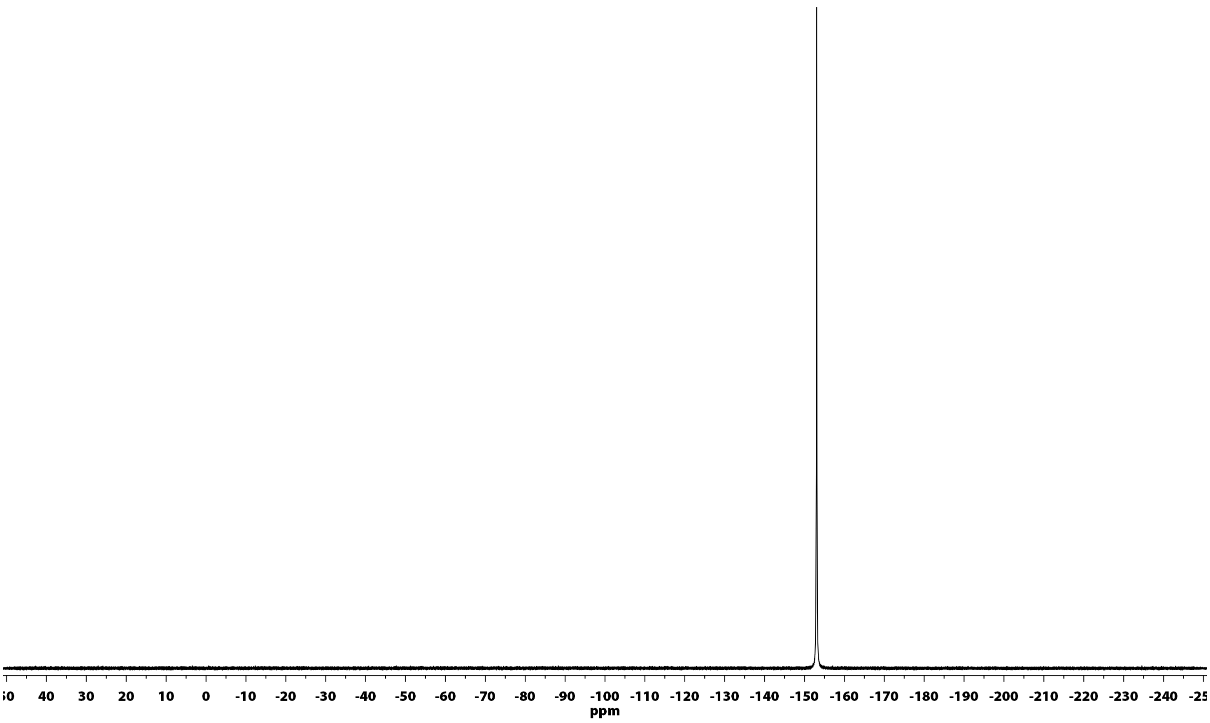

^{19}F NMR spectrum of **4** (376 MHz, CD_2Cl_2, 298 K).

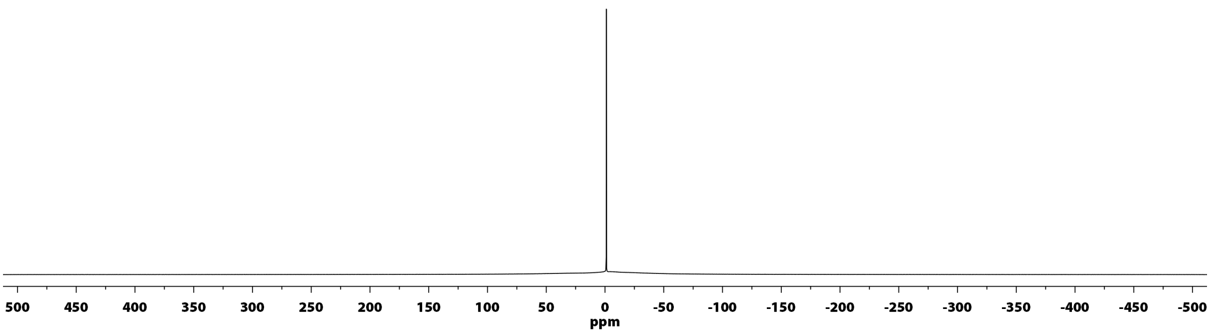

^{11}B{^{1}H} NMR spectrum of **4** (128 MHz, CD_2Cl_2, 298 K).

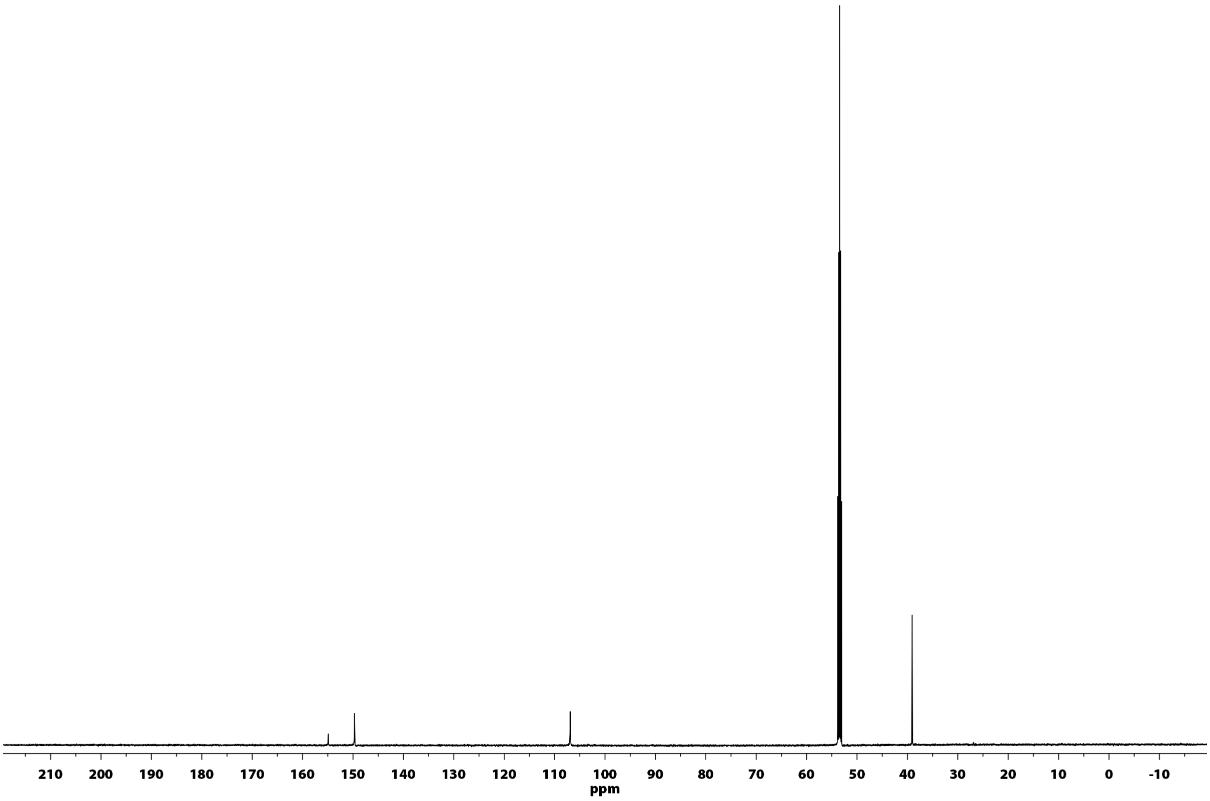

$^{13}C\{^{1}H\}$ NMR spectrum of **5** (150 MHz, CD_2Cl_2, 298 K).

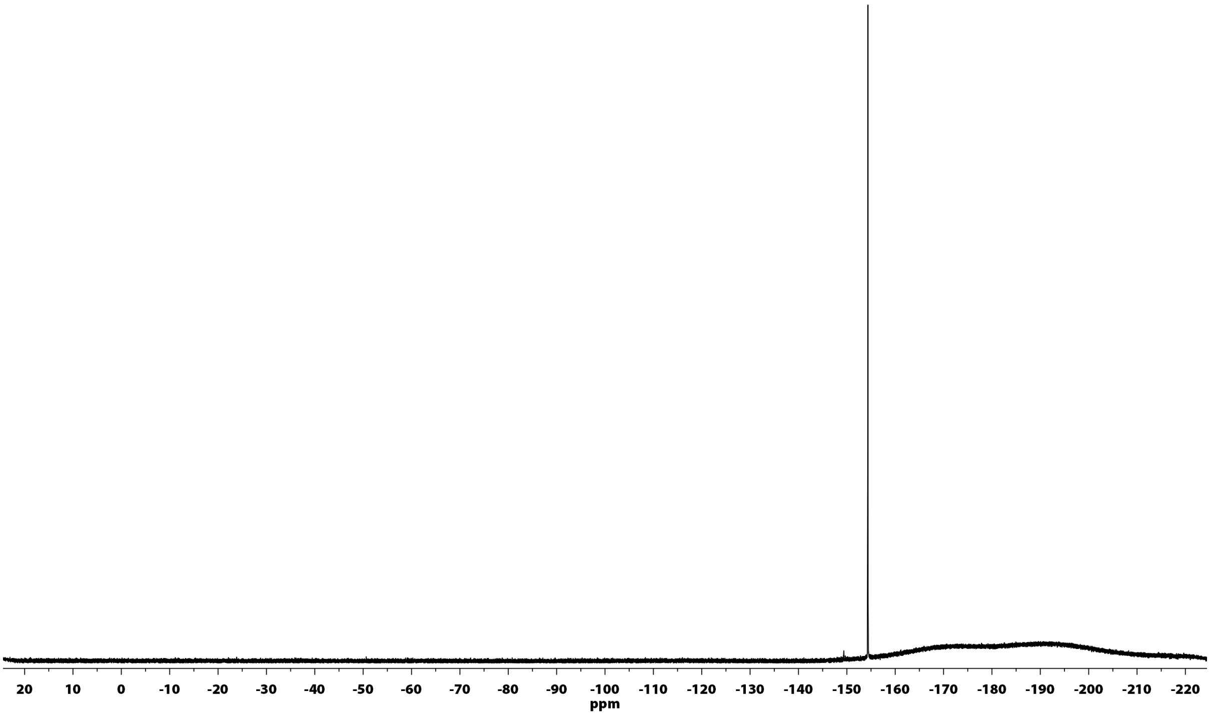

^{19}F NMR spectrum of **5** (376 MHz, CD_2Cl_2, 298 K).

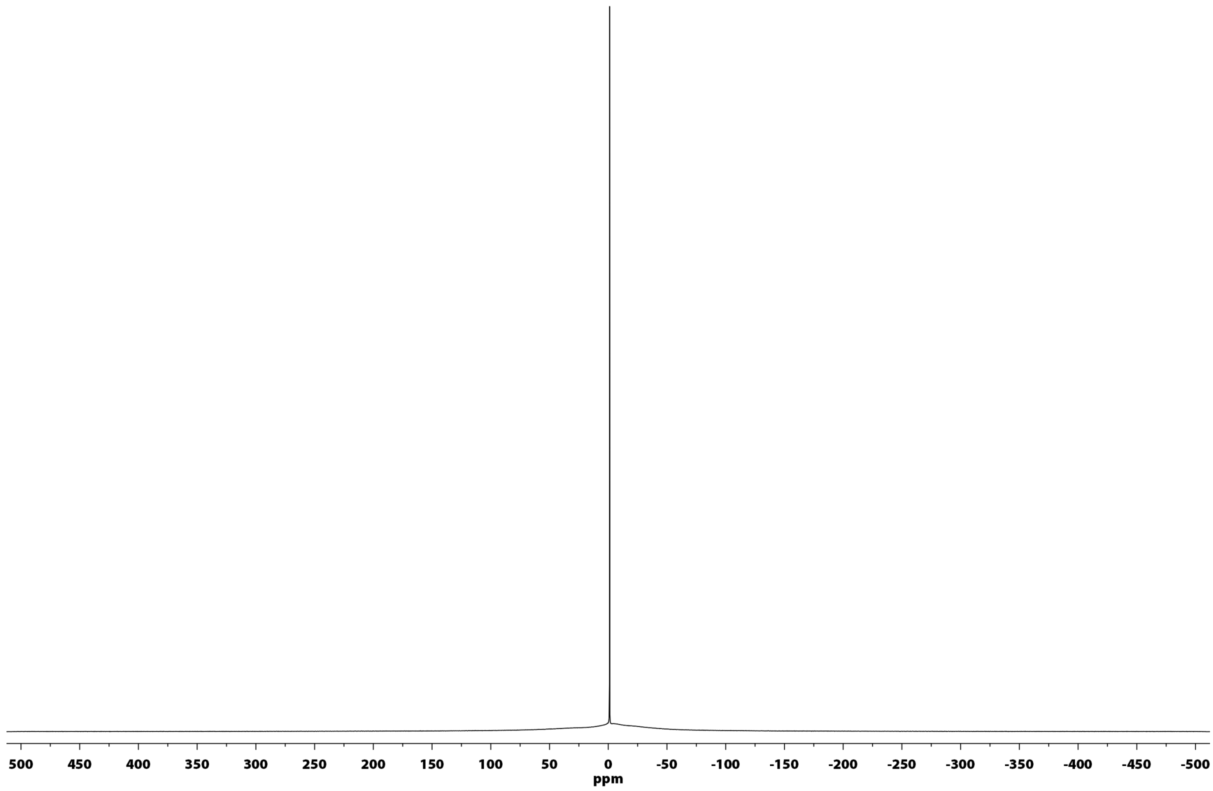

$^{11}B\{^1H\}$ NMR spectrum of **5** (128 MHz, CD_2Cl_2, 298 K).

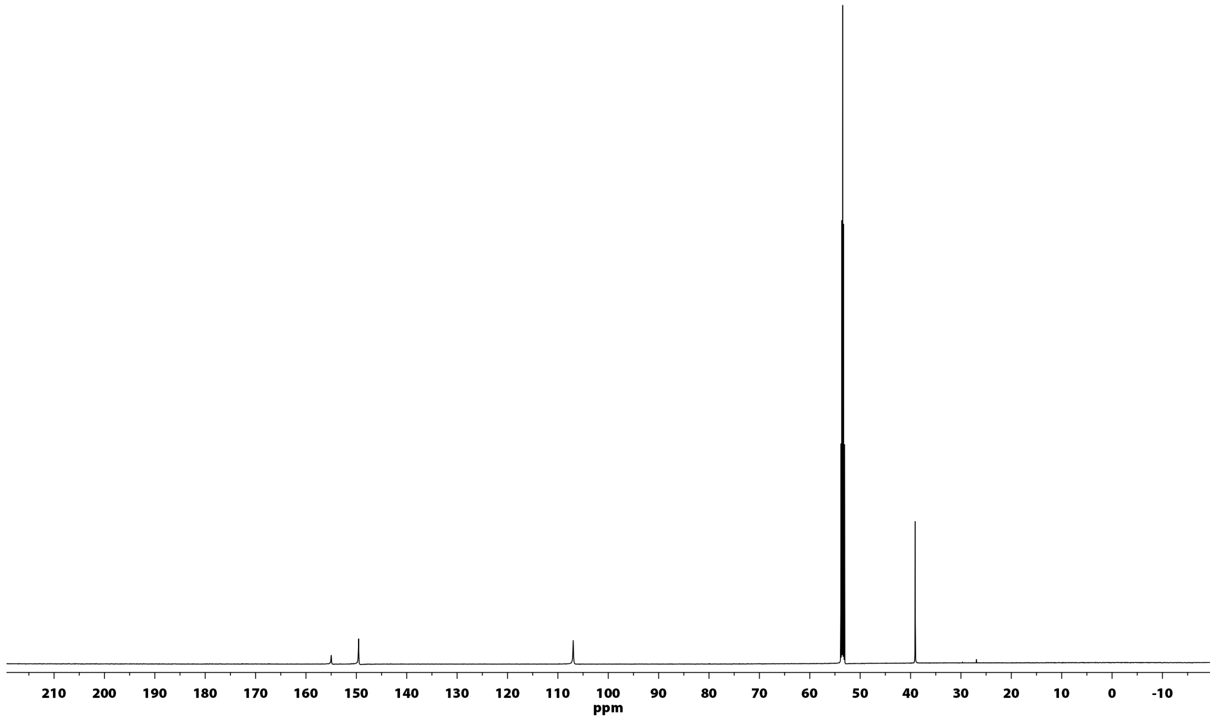

$^{13}C\{^1H\}$ NMR spectrum of **6** (150 MHz, CD_2Cl_2, 298 K).

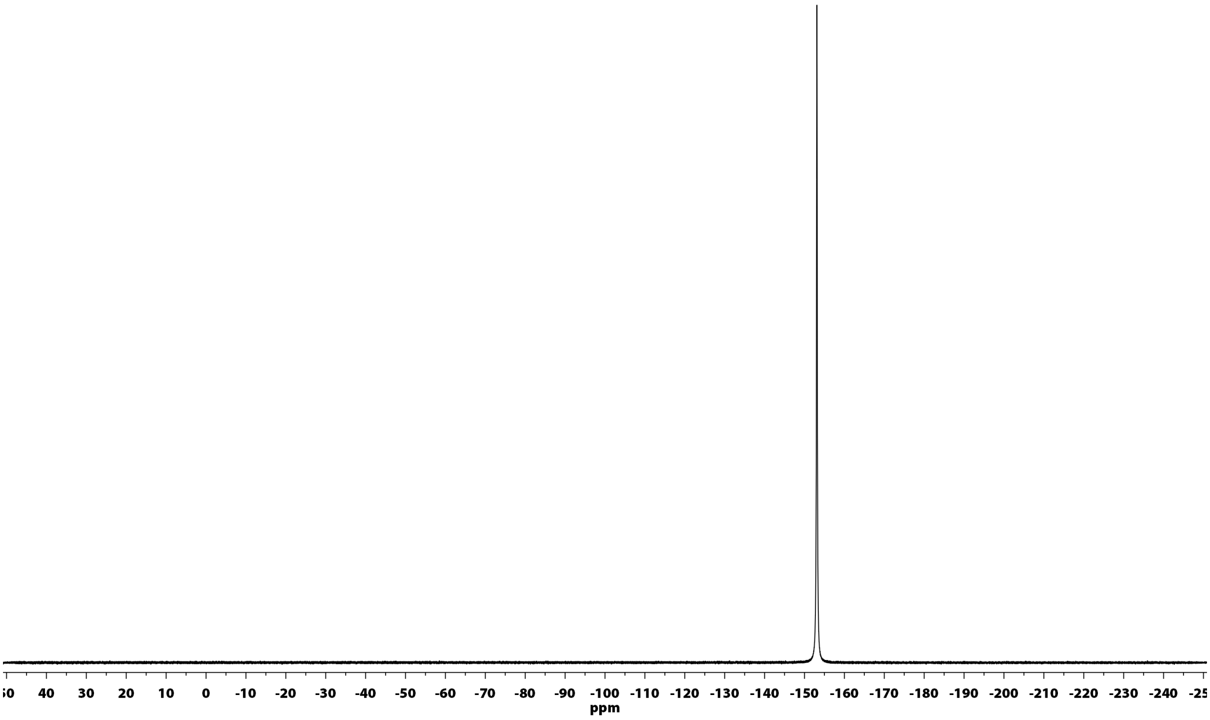

^{19}F NMR spectrum of **6** (376 MHz, CD_2Cl_2, 298 K).

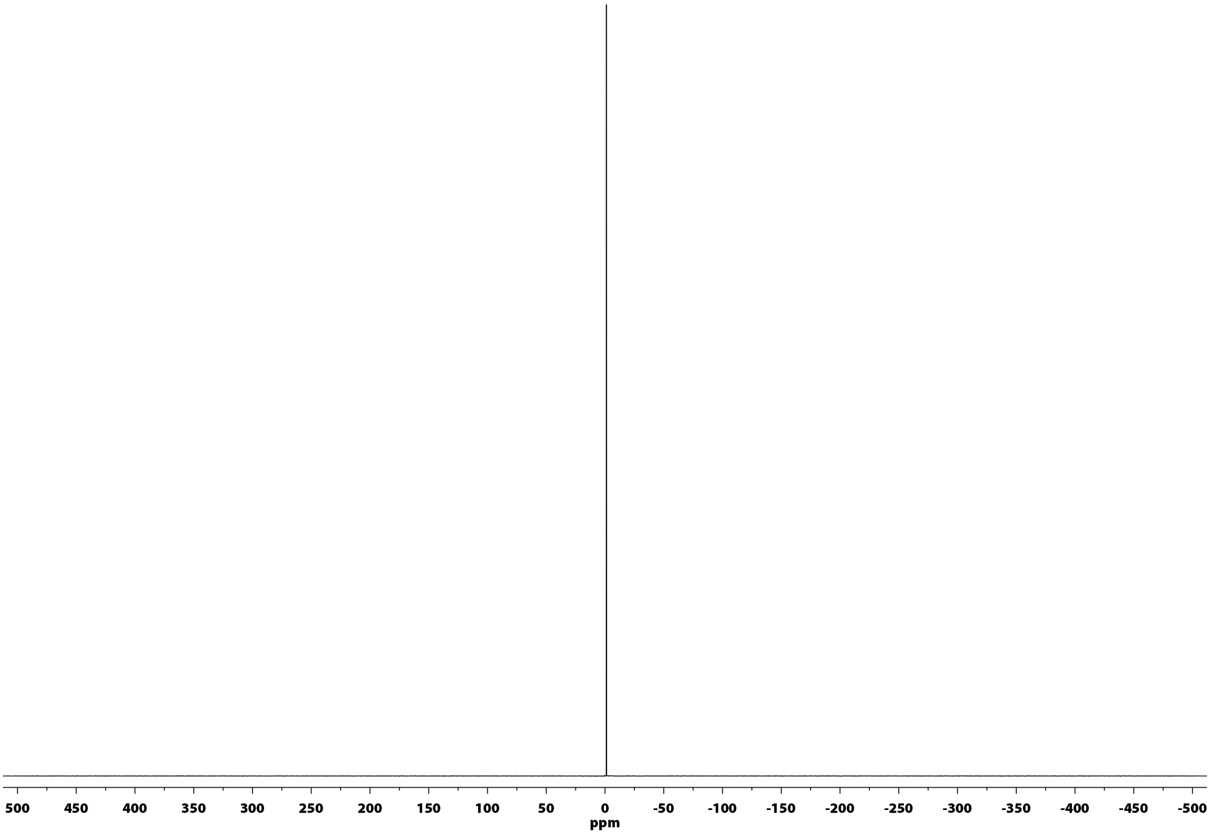

$^{11}B\{^1H\}$ NMR spectrum of **6** (128 MHz, CD_2Cl_2, 298 K).

FT-IR appendix:

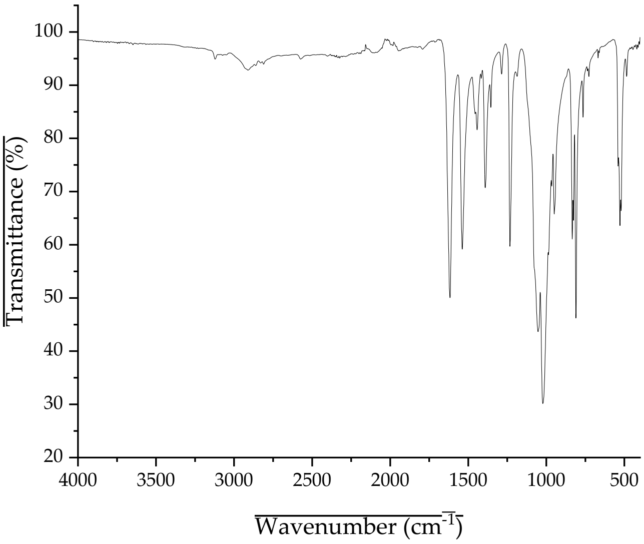

FT-IR spectrum of **1** (neat, inert atmosphere, 298 K).

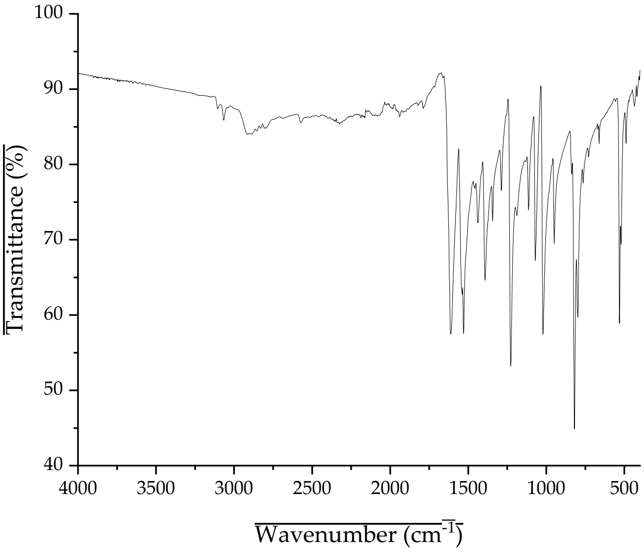

FT-IR spectrum of **2** (neat, inert atmosphere, 298 K).

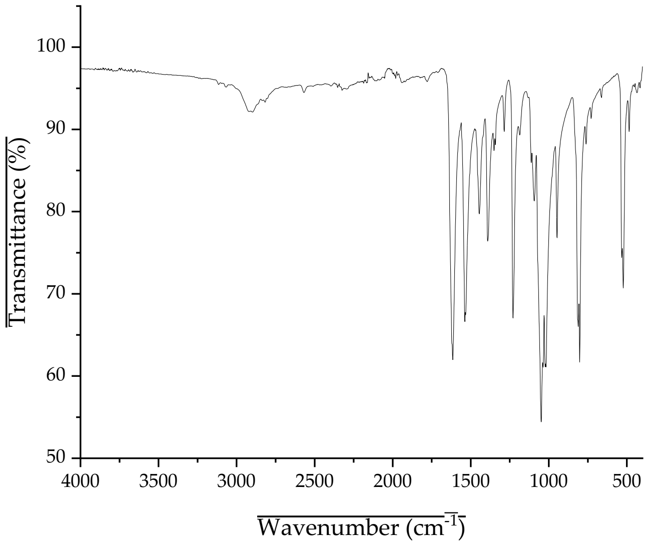

FT-IR spectrum of **3** (neat, inert atmosphere, 298 K).

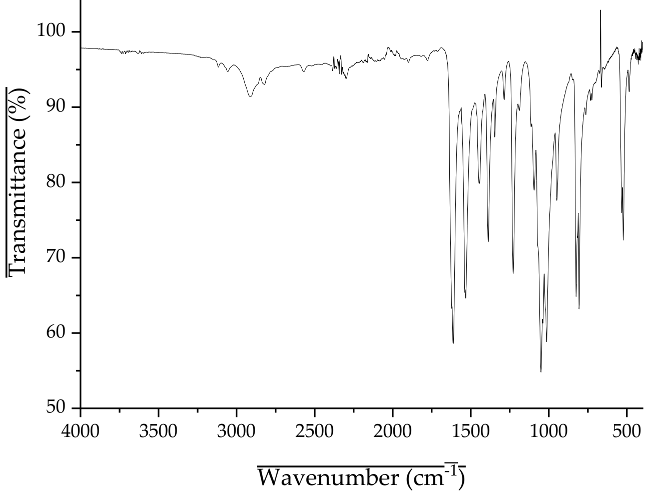

FT-IR spectrum of **4** (neat, inert atmosphere, 298 K).

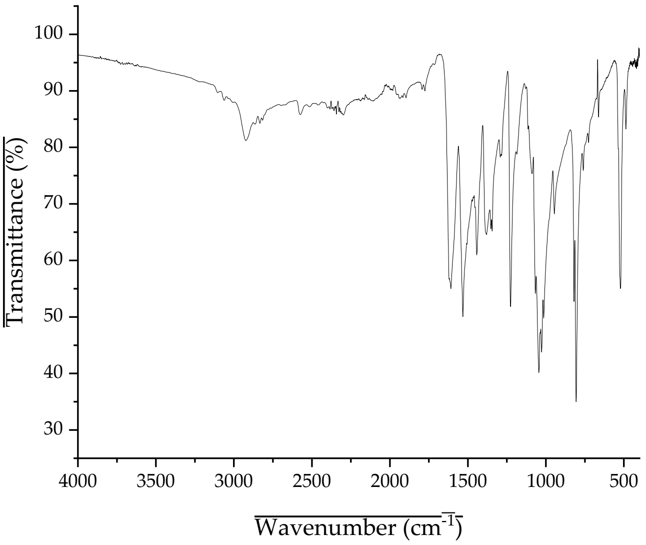

FT-IR spectrum of **5** (neat, inert atmosphere, 298 K).

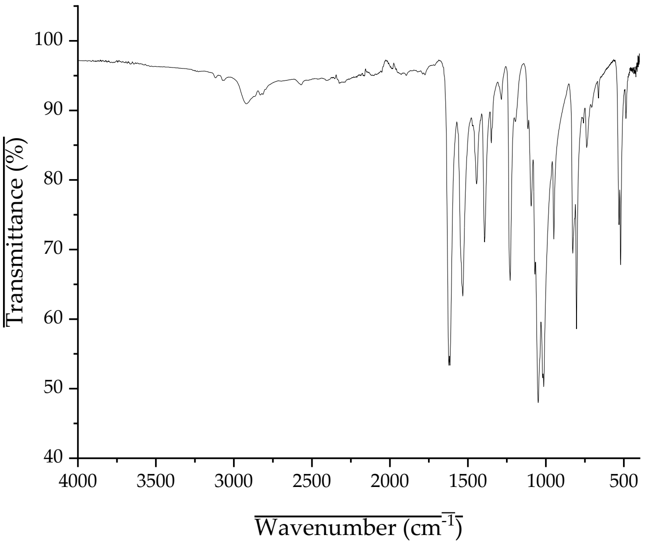

FT-IR spectrum of **6** (neat, inert atmosphere, 298 K).
